# A neural mechanism for conserved value computations integrating information and rewards

**DOI:** 10.1038/s41593-023-01511-4

**Published:** 2024-01-04

**Authors:** Ethan S. Bromberg-Martin, Yang-Yang Feng, Takaya Ogasawara, J. Kael White, Kaining Zhang, Ilya E. Monosov

**Affiliations:** 1 Department of Neuroscience, Washington University School of Medicine, St. Louis, MO USA; 2 Department of Biomedical Engineering, Washington University, St. Louis, MO USA; 3 Department of Neurosurgery, Washington University School of Medicine, St. Louis, MO USA; 4 Department of Electrical Engineering, Washington University, St. Louis, MO USA; 5 Pain Center, Washington University School of Medicine, St. Louis, MO USA

**Keywords:** Decision, Motivation, Reward

## Abstract

Behavioral and economic theory dictate that we decide between options based on their values. However, humans and animals eagerly seek information about uncertain future rewards, even when this does not provide any objective value. This implies that decisions are made by endowing information with subjective value and integrating it with the value of extrinsic rewards, but the mechanism is unknown. Here, we show that human and monkey value judgements obey strikingly conserved computational principles during multi-attribute decisions trading off information and extrinsic reward. We then identify a neural substrate in a highly conserved ancient structure, the lateral habenula (LHb). LHb neurons signal subjective value, integrating information’s value with extrinsic rewards, and the LHb predicts and causally influences ongoing decisions. Neurons in key input areas to the LHb largely signal components of these computations, not integrated value signals. Thus, our data uncover neural mechanisms of conserved computations underlying decisions to seek information about the future.

## Abstract (web summary)

Bromberg-Martin, Feng and colleagues uncover conserved value computations underlying human and monkey information-seeking behavior and show that the lateral habenula sends value signals integrating information with reward and guides online decisions.

## Main

How much would you be willing to pay to learn what your future holds? Every day we make decisions that balance our desires for concrete physical rewards, like food, water or money, with more abstract desires, like curiosity for knowledge about the future. How the brain makes these multi-attribute decisions remains unclear.

Behavioral science, psychology and economic theory propose that we choose through a process of ‘value-based decision-making’, in which we integrate the many attributes of each option together to compute its total subjective value, which then guides our choices^1^. However, our everyday decisions often require evaluating options with two quite different types of attributes. Some attributes provide extrinsic outcomes, such as food, water or money, or indicate their properties, such as their timing or variability. These are straightforward to study. We can measure them, estimate their objective value to the organism and compare this to a direct measurement of their subjective value to the organism, defined as the organism’s willingness to pay for them^2,3^. Other attributes provide intrinsic outcomes, often called abstract, cognitive or non-instrumental because they provide no apparent objective benefit to the organism and may not be physically measurable. Yet, organisms are still willing to pay for them, indicating that they have subjective value^4–6^. Thus, a critical question is how brains compute the subjective value of non-instrumental choice attributes and integrate it with the value of extrinsic rewards to guide multi-attribute decisions where both must be weighed and traded off against each other.

An especially striking form of non-instrumental preference is our desire for information about uncertain future events, a phenomenon called ‘temporal resolution of uncertainty’ in economics and ‘observing behavior’ or ‘curiosity’ in psychology^7,8^. This information seeking is not unique to humans; it occurs in animals, including monkeys, rats and pigeons^6,7,9,10^. Remarkably, humans and animals can persistently seek information about future rewards, and even pay for it, when this information has no objective value because there is no way to use it to influence the outcome^11–15^. This suggests that organisms assign information a subjective value of its own, effectively treating information itself as a form of reward. However, despite a recent explosion of research on information seeking in diverse fields, including economics, artificial intelligence, psychology, cognitive science and neuroscience, we are still only beginning to understand how the brain values information to guide decisions^6,16–22^.

Here, we address two fundamental questions. First, what common principles, if any, do humans and animals use to compute the subjective value of information about future outcomes and integrate it with the value of extrinsic rewards? This question has been remarkably unexplored, as studies of information seeking have almost exclusively examined one species at a time^6^. We address this by developing a multi-attribute information choice paradigm for both humans and monkeys. We found that human and monkey value judgements are regulated by strikingly conserved computational principles, including how they scale the value of information with uncertainty and time.

Second, what neuronal systems in the brain implement these conserved principles to compute the value of information, the value of extrinsic reward and the total value of each choice alternative to drive decisions? Recent work has identified two interconnected networks with information-related activity that are prime candidates for these roles^6^: an information prediction network, which has neurons that predict information delivery and regulate information-seeking gaze shifts^15,23^, and the reward prediction error (RPE) network, whose signals reflect preferences for both information and primary reward and potently regulate reinforcement learning^10,24–26^. However, it is unknown whether the information-related activity in these networks actually tracks the subjective value of information and whether it has a causal role in computing the total value of options to guide decisions.

To address this, we targeted two brain areas that form a junction point between these networks: the lateral habenula (LHb), an ancient epithalamic structure in the RPE network, and the anterior/ventral pallidum (Pal), a basal ganglia output nucleus that projects to the LHb and is part of the information prediction network. The LHb has been heavily implicated in value-related computations due to its strong encoding of RPEs during Pavlovian conditioning and simple decision tasks^27–29^, mirroring of and influence over RPE signals in midbrain dopamine neurons^27,30^ and causal role in reinforcement learning^31–33^. Furthermore, some LHb neurons encode prediction errors for both juice reward and information (for example, excited by ‘less juice than predicted’ and also ‘less information than predicted’), suggesting that they could integrate multiple forms of reward into a common currency of value^26^. However, little is known about whether LHb neurons quantitatively track the subjective value of options in decision-making, especially complex decisions where multiple attributes must be evaluated and weighed against each other, and whether such value signals causally influence decisions.

Similarly, subsets of Pal neurons have been reported to encode motivational signals that could be used in value computations, such as primary and conditioned reinforcement, reward uncertainty, information anticipation and RPEs^23,34–38^. Thus, Pal neurons could integrate many attributes to compute the subjective value of choice options and then relay this value to the LHb. Alternately, Pal neurons might more commonly encode mixtures of diverse motivational signals, which would need to be further integrated to compute subjective value. Indeed, previous work tested for such integrated coding in a region of the prefrontal cortex connected to the information prediction network and was consistent with the latter alternative: most neurons did not integrate information and reward into total subjective value, instead encoding them with distinct orthogonal codes^11^.

Here, we identify a neural substrate of multi-attribute decisions in the LHb. Many LHb neurons tracked the full integration of information and reward into a common currency of economic value. This value signal directly regulated decisions: trial-to-trial fluctuations in LHb value signals predicted upcoming choices, whereas injecting weak electrical current into the LHb causally perturbed upcoming choices in a manner consistent with subtracting value from the offer. By contrast, Pal neurons tracked the necessary attributes to compute value but commonly encoded them in a partially integrated manner. Thus, our work identifies the LHb as a key substrate for conserved computations integrating information and reward into the subjective value that drives multi-attribute economic decisions.

## Results

### Conserved information value in humans, monkeys, Pal and LHb

**Fig. 1:**
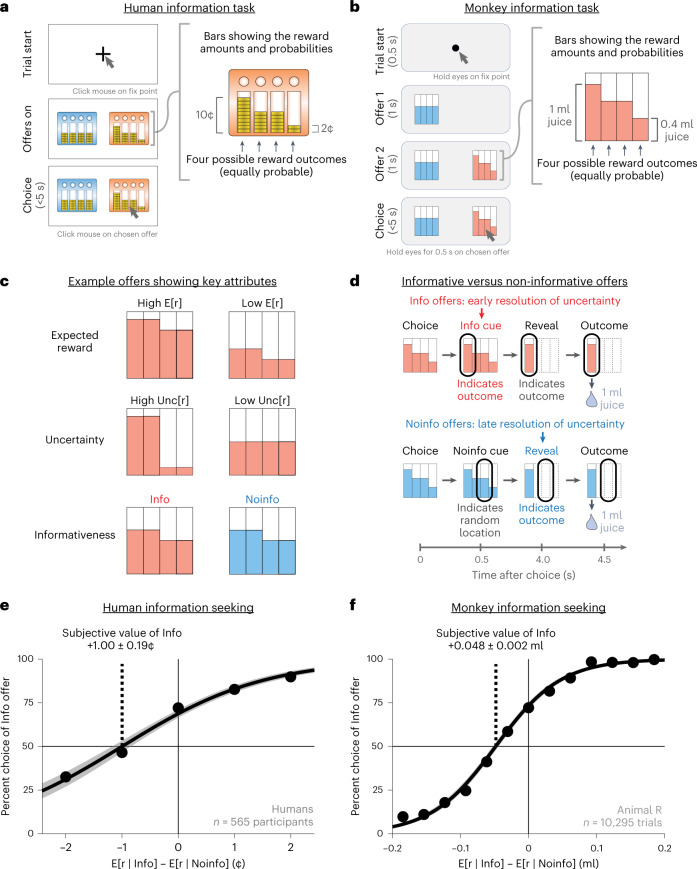
Multi-attribute information choice task for humans and monkeys. **a**,**b**, Choice procedure during the multi-attribute information choice tasks for humans (**a**) and monkeys (**b**). Each offer had four bars indicating the possible reward outcomes, with the height of each bar indicating the reward size and a color indicating whether it would provide an informative cue indicating the outcome. **c**, Examples showing offers that differ in several attributes, including expected reward (E[r]), uncertainty (Unc[r]) and informativeness (Info versus Noinfo). **d**, Info offers granted access to informative cues indicating the upcoming reward outcome (red), whereas Noinfo offers did not (blue). For the monkey task shown here, all offers also had a final reveal shortly before outcome delivery to allow animals to physically prepare to consume the juice rewards in all trials. **e**, The human population assigned positive subjective value to info. A psychometric curve measured the subjective value of information based on the choice of Info versus Noinfo offers (*y* axis) as a function of the difference in their expected reward (*x* axis) computed using all *n* = 565 human participants. Data are shown as mean ± s.e. (too small to see). The curve and shaded area are the best-fitting logistic function ± bootstrap s.e. (*n* = 200 bootstraps). The text indicates the subjective value of information implied by the curve’s indifference point and its bootstrap s.e. **f**, Same as in **e** for one example animal (animal R).

We assessed how humans value information in a multi-attribute information choice task (Fig. 1a). Participants (*n* = 565) chose between a pair of offers on each trial with a mouse click. When chosen, each offer provided a monetary reward that was randomly drawn from a set of four possible outcomes, depicted as four stacks of coins. Each coin was worth $0.01. On average, participants earned a total of $9.29 from their choices in the task. Different offers provided different probability distributions of rewards, which could have different levels of reward expectation (E[r]; the mean height of the stacks) and uncertainty (Unc[r]; the variability of the height of the stacks; Fig. 1c). Each offer also had a color that indicated whether it was an Info or Noinfo offer (Fig. 1a,c,d). When chosen, Info offers provided an informative cue indicating which of the four possible outcomes would be delivered into the participant’s winnings on that trial, whereas Noinfo offers did not. Importantly, this information was non-instrumental and hence had no objective value because there was no way to use it to influence the outcome^6^, and participants were clearly instructed on this. Thus, each offer had multiple attributes corresponding to multiple features of the reward distribution and cue informativeness. To dissociate information preferences from color preferences, the mapping between color and informativeness was randomized for each participant and reversed midway through the session.

The information choice task for monkeys (Fig. 1b; *n* = 4) had an analogous design. Animals freely viewed and then chose from a pair of offers on each trial. Each offer gave a reward randomly drawn from a set of four possible outcomes, depicted as four bars whose heights indicated their magnitudes. Offers had distinct textures indicating whether they were Info offers that gave an informative cue indicating the upcoming outcome or Noinfo offers that gave a non-informative cue. The main differences between tasks were that monkeys were rewarded with juice (not money), chose with eye movements (not mouse clicks), were shown offers sequentially (to allow measurement of neural responses to each offer) and received their juice reward at the end of each trial (unlike humans who got their money rewards as a lump sum after the experiment). Also, to ensure that animals had ample opportunity to physically prepare to drink their reward on every trial^10,39^, animals were always shown a ‘reveal’ stimulus revealing the outcome shortly before its delivery (Fig. 1d). We confirmed that humans had consistent behavior in a task version with a similar ‘reveal’ stimulus (Methods; see section ‘Information value scales with time’ and Supplementary Fig. 1). Also, we confirmed that monkeys had consistent behavior in a task version where offers had additional visual attributes indicating cue and reward delivery times (the version used for neuronal recording; see section ‘Information value scales with time’ and Supplementary Fig. 2).

Using these analogous tasks, we found analogous valuation of information by humans, monkeys and neurons. We first assessed the fundamentals of how individuals and neurons valued Info versus Noinfo offers and investigated the algorithms they used to compute these values. In humans, many individuals were strongly information seeking. On average, humans chose informative over non-informative offers 69% of the time, and this preference was significant in 69% of participants. Humans placed high subjective value on information, as measured by their willingness to pay for it (Fig. 1e), which, on average over all trials, was 1.00 ± 0.19 cents, fully 17% of the mean expected reward of offers in the task. In monkeys, all animals modulated their choices with information (*P* < 0.0001 in each animal; Supplementary Fig. 3). For example, on average over all trials, the animal shown in Fig. 1f was willing to pay 0.048 ± 0.002 ml of juice for information, fully 16% of the mean expected reward of offers in the task (Supplementary Table 1). As monkeys performed the task, we recorded from neurons in the LHb and Pal and examined their offer responses (*n* = 2 animals, *n* = 375 LHb, *n* = 294 Pal). Many neural offer responses were modulated by informativeness (LHb 19.1% and Pal 28.4%; Supplementary Fig. 4; main effect of Info, *P* < 0.05 in generalized linear model (GLM) fits to neuronal activity; Model 8 in the Supplementary Modeling Note). Consistent with previous work, LHb neurons predominantly encoded information and other attributes with negative signs (lower firing rate for preferred attributes), whereas Pal neurons had both negative and positive signs (Supplementary Fig. 5).

### Information value scales with uncertainty

We next sought to use this task to answer our key questions. First, what principles do individuals follow when they use attributes of instrumentally valuable extrinsic rewards, like money and juice, to compute the subjective value of non-instrumental choice attributes, like information about future outcomes? To do so, we tested how the value of information scales in our task with two major determinants of instrumental reward value: expected reward and reward uncertainty. Second, are these principles conserved between humans and monkeys and in the information-related signals in Pal and LHb?

**Fig. 2:**
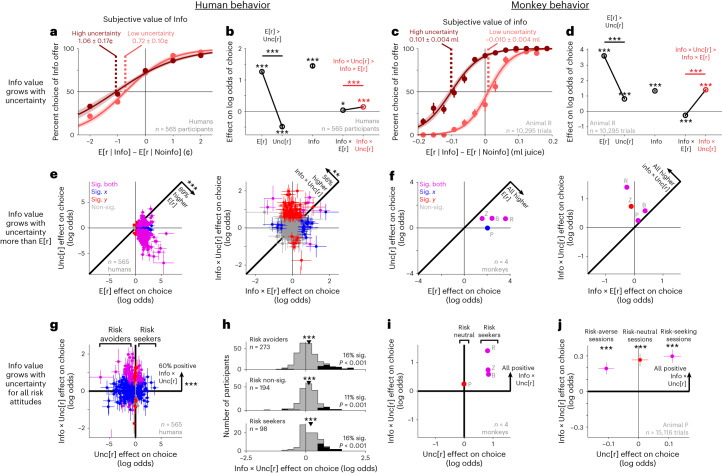
Information value grows with uncertainty in humans and monkeys. **a**, The human population assigned greater value to information about uncertain rewards. Data are the same as in Fig. 1e but analyzed separately for trials where both offers had high or low reward uncertainty (dark or light red). The shaded area represents ±bootstrap s.e. **b**, Mean fitted GLM weights of offer attributes. Error bars represent ±s.e.; *P < 0.05; **P < 0.01; ***P < 0.001; signed-rank tests. **c**,**d**, Same as **a** and **b**, respectively, for animal R. **e**, Humans generally placed higher value on expected reward (left, E[r] versus Unc[r]) but increased the value of information more with uncertainty (right, Info × E[r] versus Info × Unc[r]). Data are fitted parameters ± s.e. from each individual. Colors indicate that neither coordinate (gray), the *x* coordinate (blue), the *y* coordinate (red), or both (purple) are significant (*P* < 0.05; *t*-tests). The text indicates the fraction of individuals above or below the identity line and its significance (binomial tests). For visual clarity, axes exclude one extreme outlier (error bar visible at the bottom of **e** and **g**); Sig., significant; Non-sig., non-significant. **f**, Same as **e** for all animals. The gray text indicates which data point came from which animal. The error bars are too small to see (all s.e. < 0.08). **g**, The value of information grows with uncertainty, as indicated by positive weights of Info × Unc[r] (*y* axis) for humans with negative, non-significant or positive weights of Unc[r] (*x* axis). The text indicates the fraction above *y* = 0 and its significance (binomial test). **h**, Histograms of fitted Info × Unc[r] weights for individuals classified as risk avoiders, non-significanters or seekers. The black histogram shows significant weights, and the text indicates the fraction of individuals with significant positive weights and the *P* value for whether this is greater than chance (one-tailed binomial tests). **i**, Same as **g** for all animals. Error bars are too small to see (all s.e. < 0.08). **j**, In animal P, the value of information grew with uncertainty (positive Info × Unc[r] weight; *y* axis), consistently in sessions when the animal tended to be risk averse, neutral or seeking (Unc[r] weight; *x* axis; *n* = 5,248, 5,102 and 4,766 trials, respectively). Data are shown as mean ± s.e. See Supplementary Table 6 for the details of all tests and *P* values.

To test this, the task included reward distributions that were safe, where the outcome was entirely certain, or risky, where the outcome was uncertain (Figs. 1c and 2a,c). We analyzed offers separately based on their uncertainty and found that the value of information scaled strongly with uncertainty in both species. Humans were willing to pay an average of 1.06 cents for information about uncertain outcomes but only 0.72 cents for information about certain outcomes (Fig. 2a). Similarly, all individual monkeys were willing to pay more for information about uncertain outcomes (Fig. 2c and Supplementary Fig. 3).

To quantify this, we fit each individual’s choices with a GLM with separate weights for each offer attribute and their interactions with information. This framework models the subjective value of each offer as a linear weighted combination of its attributes and interactions. For this initial analysis, we defined Uncertainty as the standard deviation (SD) of the reward distribution (Model 1). We found that the human population had a strong positive Info × Uncertainty weight, indicating that information was subjectively valued more highly when it would resolve a larger amount of reward uncertainty (Fig. 2b). The Info × Uncertainty weight was positive in 60% of individual humans; it was significant in 14% of individuals, which were predominantly positive (Fig. 2e; 12% positive, more than expected by chance, *P* < 0.0001, binomial test; 2% negative, not different from chance, *P* = 0.89). Similarly, all individual monkeys had significant positive Info × Uncertainty weights (Fig. 2d,f).

Crucially, the value of information predominantly scaled up with uncertainty, not simply with all attributes that individuals cared about. All monkeys and nearly all humans placed much greater weight on E[r] than Unc[r] (Fig. 2e,f), as expected from prior work. Yet the opposite held for the interactions between these attributes and information: all monkeys and many humans placed greater weight on Info × Unc[r] than on Info × E[r] (Fig. 2e,f). In both species, the Info × Unc[r] effects were overwhelmingly positive (humans 14.2% significant, 12.4% positive and 1.8% negative; monkeys 4/4 significant positive; Fig. 2e,f), whereas Info × E[r] effects were less commonly positive (humans 5.3% significant, 4.1% positive and 1.2% negative; monkeys 3/4 significant, 2 positive and 1 negative; Fig. 2e,f). Thus, both species placed more value on expected reward than uncertainty but increased the value of information more with uncertainty than expected reward. Remarkably, this Info × Unc[r] effect was still generally positive even for occasional individuals with negative main effects of Info (Supplementary Fig. 3). Thus, even individuals averse to information still generally valued it more positively when it resolved more uncertainty.

These data demonstrate that humans and monkeys doing similar tasks can value information in similar ways. Using similar tasks was important to reduce the possibility that differences in information seeking could simply arise from differences between tasks (as reported in humans^6^). One task difference was that humans learned the task from reading explicit instructions. This raises the possibility that humans might misunderstand instructions and mistakenly believe that information had instrumental value. However, control analysis of a postexperiment questionnaire indicated otherwise. The scaling of information value with uncertainty was driven by participants who reported that offer color indicated informativeness, not yield of coins (Supplementary Figs. 6 and 7). Also, some humans paid for information about certain outcomes (Fig. 2a). These humans may have sought an extra visual cue or confirmation that a monetary reward would be delivered to their accounts, unlike monkeys who could confirm their juice reward when it was delivered to their mouths. Regardless, control analysis showed that this occurred even in humans who reported that offer color indicated informativeness and not yield of coins, and humans with or without this phenomenon still scaled the value of information with uncertainty (Supplementary Fig. 8). Finally, monkeys tended to have higher Info × Uncertainty effects than humans (Fig. 2e,f; all monkey effects are higher than the mean human effect). Monkeys may have stronger or more consistent motivation due to working for appetitive reward in a controlled experimental environment with extensive training. Monkeys also tended to have higher E[r] effects (Fig. 2e,f; all monkey effects are higher than the mean human effect).

We next asked how these attitudes toward information about risky outcomes are related to attitudes toward risk itself. This topic has a long history in economic theory^8,40,41^ and psychological and computational models of information seeking^12,42–46^. In some theories, information to resolve uncertainty is valued independently as an incentive in its own right and hence can have positive subjective value regardless of whether an individual is risk seeking or risk averse^6,12,41,42^. In other theories, attitudes toward information and attitudes toward risk derive from closely analogous mechanisms, typically involving placing disproportionate weight on future events depending on their desirability^7,8,40,44,45^. If a single underlying mechanism is responsible, then information preferences should be related to risk preferences^10,40,44,45^.

Our data support the former theories in both across-species and within-species comparisons. Across species, humans and monkeys had predominantly opposite risk attitudes, consistent with previous work^47,48^. Humans were mostly risk averse, whereas monkeys were mostly risk seeking (negative versus positive weight of Uncertainty; Fig. 2g,i). Yet, both the human population and all monkeys were fit with positive weights of Info × Uncertainty, indicating that they placed higher value on information about risky, uncertain offers (Fig. 2g,i). Within humans, the Info × Uncertainty weight was positive in humans with each possible risk attitude (risk averse, risk seeking and risk non-significant; Fig. 2h and Supplementary Fig. 9). Similarly, within monkeys, it was positive in all animals regardless of whether they were risk seeking or risk neutral (Fig. 2i) and positive even in sessions when the one risk-neutral animal had trends for each possible risk attitude (animal P; Fig. 2j). Thus, our data are consistent with individuals treating information that resolves uncertainty about future outcomes as a separate incentive in its own right, remarkably distinct from their attitudes toward uncertainty about future outcomes itself.

**Fig. 3:**
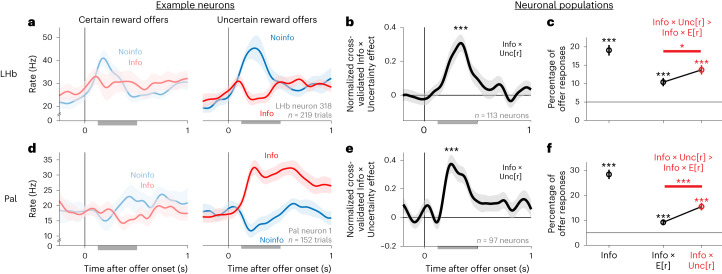
Information signals grow with uncertainty in LHb and Pal neurons. **a**, An example LHb neuron with a strongly information-related offer response that is much stronger for offers with reward uncertainty (right) than for offers with certain reward (left). Gray bars indicate the analysis window (0.125–0.5 s). Data are shown as mean ± s.e. **b**, Mean Info × Unc[r] effect, measured as the cross-validated difference in normalized activity between Info and Noinfo offers with uncertain rewards minus the analogous difference for certain rewards. This analysis uses all *n* = 113/303 attribute-responsive neurons selected for having a significant Info × Unc[r] effect by the cross-validation procedure (Methods). The shaded area shows ±1 s.e.; ***P < 0.001; signed-rank test. **c**, Percentage of all LHb offer responses (*n* = 375 total neurons, two offers) with significant GLM weights of Info, Info × E[r] and Info × Unc[r]. The horizontal line represents chance. Data are fitted parameters ± s.e.; *P < 0.05; ***P < 0.001; one-tailed binomial tests; comparisons were performed with signed-rank tests. **d**–**f**, Same as **a**–**c** for Pal neurons (*n* = 97/251 attribute-responsive neurons selected as significant Info × Unc[r] effect, *n* = 294 total). See Supplementary Table 6 for the details of all tests and *P* values.

This scaling of information value with uncertainty could be implemented by the Pal and LHb. The information-related activity of many neurons in these areas was significantly higher for uncertain rewards than for certain rewards (Fig. 3a,b,d,e and Supplementary Fig. 10). To quantify this, we fit each neuron’s activity with an analogous GLM to the behavioral model (Model 13). Many neural offer responses had Info × Unc[r] effects, and fewer had Info × E[r] (Fig. 3c,f; LHb *P* = 0.041, Pal *P* = 0.0007; signed-rank test).

### Information value scales with a specific form of uncertainty

**Fig. 4:**
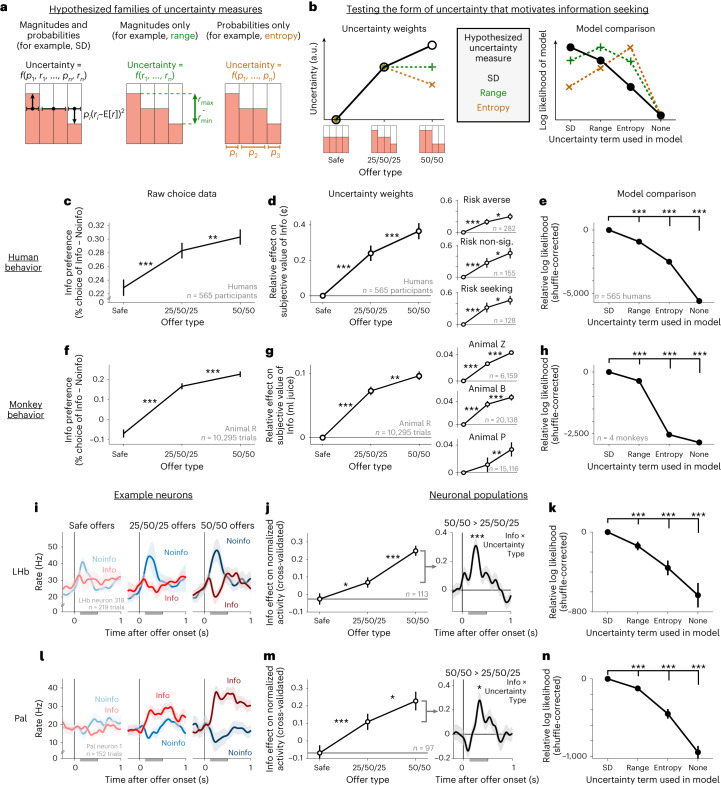
Information value grows with a conserved form of uncertainty. **a**, Families of uncertainty measures hypothesized to influence behavior using reward magnitudes and probabilities, magnitudes only or probabilities only (for example, SD, range and entropy). **b**, We presented individuals with three offer types (safe, 25/50/25 and 50/50) to dissociate these measures. Plots show their hypothesized effects on the subjective value of information (left) and fit quality (right); a.u., arbitrary units. **c**–**e**, Human information seeking is motivated by an SD-like form of uncertainty. **c**, Mean difference in percentage choice of Info versus Noinfo offers separately for each offer type. Error bars represent ± s.e.; *P *<* 0.05; **P *<* 0.01; ***P < 0.001; signed-rank tests. **d**, Mean fitted GLM weights for the effect of each uncertainty type on the subjective value of information. Insets show similar results for the subsets of participants classified as risk averse, risk non-significant or risk seeking. To ensure that this plot is not biased for any specific uncertainty measure, participants were classified as risk averse/seeking if they had a significant negative/positive Unc[r] effect according to any of the three models (SD, range or entropy; Supplementary Table 2). Also, to illustrate the willingness to pay for information, fitted parameters were scaled based on the fitted effect of E[r] to convert them from units of log odds to units of reward (money). **e**, Model comparison with shuffle-corrected log likelihoods relative to the SD model. Error bars represent ±bootstrap s.e. (*n* = 2,000 bootstraps); three asterisks (***) indicate the 99.9% bootstrap confidence interval excluding 0. **f**–**h**, Same as **c**–**e** but for animals, showing similar results. Data in **f** and **g** are from animal R; insets in **g** show each additional animal. **i**–**k**, LHb neuron information-related activity scales with an SD-like form of uncertainty. **i**, The same LHb neuron from Fig. 1 but separating its responses by offer type, revealing stronger information-related activity for 50/50 offers. The shaded area represents ±s.e. **j**, Left, SD-like form of uncertainty revealed by mean cross-validated GLM weights from fits to attribute-responsive LHb neurons with significant Info × Unc[r] effects. Error bars represent ±s.e.; *P < 0.05; ***P < 0.001; signed-rank tests. Right, mean time course of the cross-validated Info × Uncertainty Type effect on neuronal activity, measuring the enhancement of information-related activity by 50/50 relative to 25/50/25 offers (Methods). Data are shown in the same format as in Fig. 3b,e. **k**, Model comparison favors an SD-like form of uncertainty. Data are shown in the same format as in **e**. **l**–**n**, Same as **i**–**k** for the Pal. Model comparisons were performed using all neurons where the model converged to a stable fit in all bootstrap samples (*n* = 373 LHb, *n* = 293 Pal). See Supplementary Table 6 for details of all tests and *P* values.

To understand why and how the value of information scales with uncertainty, it is fundamental to uncover the specific form of uncertainty that governs information seeking. Many mathematical forms of uncertainty have been proposed to influence cognition and behavior. However, most neuroscience studies manipulate uncertainty using single parameters of probability distributions, which makes it hard to differentiate these proposals (Supplementary Fig. 11). Here, we use our task to test three hypothesized families of uncertainty measures (Fig. 4a,b): (1) uncertainty measures that only depend on outcome probabilities, like Shannon entropy^49^, which are proposed to regulate information preferences^42,50,51^ and many other neural computations^52,53^; (2) measures that only depend on outcome magnitudes, like range, which are proposed to regulate the dynamic range of activity^54–56^; and (3) measures that consider both probabilities and magnitudes, like SD and variance, which are proposed to regulate risk- and information-related behavior and activity^12,48,57^.

To dissociate these families of uncertainty measures, we used two types of uncertain reward distributions: 50/50 offers, where big or small rewards each occurred 50% of the time, and 25/50/25 offers, where big or small rewards each occurred 25% of the time and intermediate rewards occurred the remaining 50% of the time (Fig. 4b). Uncertainty measures that only depend on probabilities, like entropy, are highest for the latter because it has more evenly spread probabilities, measures like range are indifferent because both have the same extreme big and small reward magnitudes, and measures like SD are highest for the former because it has the highest probability of large deviations from the mean magnitude.

Both humans and monkeys valued information based on an uncertainty measure more closely resembling SD than either range or entropy, as indicated by raw choice percentages (Fig. 4c,f) and fits to behavior with separate Info × Uncertainty weights for each of the two types of uncertain offers (Fig. 4d,g and Model 5). This was the case for humans with all risk attitudes and for each individual monkey (Fig. 4d,g). To further quantify this, we performed a formal model comparison between models of behavior in which the uncertainty measure was SD, range, entropy or none (Models 1–4). We found that SD was highly favored in both species (Fig. 4e,h) and task versions (Supplementary Fig. 12). This was also true for LHb and Pal information signals. The same example neurons from Fig. 1 had stronger information signals for offers with 50/50 distributions than 25/50/25 distributions (Fig. 4i,l). Similarly, the population average activity had a clear Info × Uncertainty Type interaction (Fig. 4j,m, Supplementary Fig. 10 and Model 12), with a similar time course to the basic Info × Uncertainty effect (Fig. 3b,e). Again, model comparisons favored SD (Fig. 4k,n and Models 8–11). Thus, the Pal and LHb tracked a form of uncertainty resembling that which governed information’s value in monkeys and humans.

### Information value scales with time

**Fig. 5:**
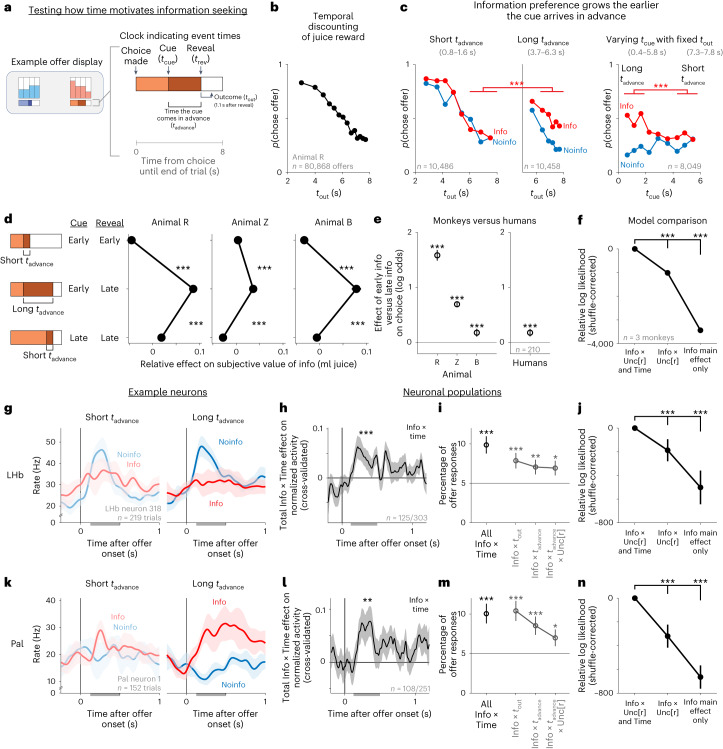
How information value grows with time. **a**, We tested how information value scales with time by adding a ‘clock’ stimulus to each offer indicating the timing of cues (*t*_cue_) and reveals (*t*_rev_) and hence their difference, the time the cue comes in advance (*t*_advance_). **b**, Conventional temporal discounting of delayed juice rewards. Plotted is the choice probability of offers as a function of outcome delivery time (*t*_out_) for animal R, showing a strong preference for early rewards. Data are fitted parameters ± bootstrap s.e. (too small to see). **c**, Information preference grows the earlier the cue arrives in advance. Left, data are shown in the same format as in **b** but are split into Info and Noinfo offers, and only offers with short *t*_advance_ are plotted. Middle, the same but only plotting offers with long *t*_advance_, showing increased preference for information; three asterisks (***) indicate a greater difference between Info and Noinfo offers when *t*_advance_ was long (the 99.9% bootstrap confidence interval excluded 0), when considering the same range of *t*_out_ (6–8 s). Right, data are shown in the same format, but choice as a function of *t*_cue_ is plotted while only including offers with *t*_out_ in a narrow fixed range (7.3–7.8 s), showing greater preference for Info when *t*_cue_ is early and hence *t*_advance_ is long. **d**, Fitted relative effect on the subjective value of Info of three types of offers defined by *t*_cue_ and *t*_rev_. All three animals tested in the task placed higher value on Info for offers with early *t*_cue_ and late *t*_rev_ (that is, the longest *t*_advance_). Error bars represent ±s.e.; ***P < 0.001; two-tailed *t*-test of difference in GLM weights. To illustrate the willingness to pay for information, fitted parameters were scaled based on the fitted effect of E[r] to convert from units of log odds to units of reward (juice). **e**, Cross-species comparison of the value of Early versus Late Info. Shown is the mean fitted effect of Early versus Late Info on choice for each animal (left; *n* = 40,434, 87,491 and 10,295 trials from animals R, Z and B; Methods) and for the human population tested on a version of the human task that manipulated information timing (right; different participants from the task in Figs. 1–4; Methods). Error bars represent ±bootstrap s.e. Three asterisks (***) indicate the 99.9% bootstrap confidence interval excluding 0. **f**, Model comparison favors a model including interactions between Info and both uncertainty- and time-related variables. Data are shown in the same format as in Fig. 4e. Error bars represent ±bootstrap s.e. **g**–**j**, LHb neuron information-related activity is commonly modulated by information timing. **g**, The same LHb neuron from Figs. 1 and 2 showing information-related activity when *t*_advance_ is short versus long. Data are shown as mean ± s.e. **h**, The LHb population had a significant total cross-validated effect of Info × Time interaction terms (Info × *t*_out_, Info × *t*_adv_, Info × *t*_adv_ × Unc[r]) using the 125/303 attribute-responsive LHb cells selected by the cross-validation procedure. The shaded area represents bootstrap ±s.e.; ***P < 0.001; signed-rank test. **i**, Percentage of LHb offer responses (*n* = 375 total neurons, two offers) with significant GLM weights of Info × *t*_out_, Info × *t*_adv_ and Info × *t*_adv_ × Unc[r]. The horizontal line represents chance. The leftmost data point was computed by pooling *P* values from the three individual effects (Fisher’s combination test^98^). Data are shown as mean ± s.e.; *P < 0.05; **P < 0.01; ***P < 0.001; one-tailed binomial test. **j**, Model comparison. Data are shown in the same format as in **f**. **k**–**n**, Same as **g**–**j** for Pal neurons. Data in **l** were computed using the 108/251 attribute-responsive Pal neurons selected by the cross-validation procedure. Model comparisons were performed using all neurons where the model converged to a stable fit in all bootstrap samples (*n* = 373 LHb, *n* = 293 Pal). See Supplementary Table 6 for details of all tests and *P* values.

We next asked whether the value of information scales with time. Information preferences are known to be influenced by task timing^10,39,45,58^, but we are only beginning to understand how this translates into subjective value and how this value is computed by neural circuits^59^. Notably, whereas conventional temporal discounting scenarios only require evaluating a single event, the time of the extrinsic reward outcome (*t*_out_) information seeking also requires evaluating the time when information will arrive: the time of the cue (*t*_cue_) for Info offers and the time of the final reveal (*t*_rev_) for Noinfo offers (Figs. 1d and 5a). We hypothesized that these are crucial ingredients for information valuation. Specifically, the difference between them, the time the cue comes in advance of the reveal (*t*_advance_), may be valued because it quantifies the temporal advantage in information: how much earlier an individual will get information by choosing Info.

To test this in monkeys, we modified the task to augment each offer with a visual ‘clock’, with three segments indicating the time durations between choice and cue, between cue and the reveal stimulus, and between the reveal and the end of the trial (Fig. 5a and Supplementary Fig. 2; *n* = 3 animals). After learning, monkeys retained their positive Info × Uncertainty effects while additionally showing strong time preferences (Supplementary Fig. 3). As expected, animals showed strong conventional temporal discounting, preferring early *t*_out_ (Fig. 5b and Supplementary Fig. 3).

Crucially, time also strongly influenced information preferences in a manner that depended on *t*_advance_. That is, all animals were more likely to choose informative offers when *t*_advance_ was long than when *t*_advance_ was short (Fig. 5c and Supplementary Fig. 13). Importantly, information preference depended on *t*_cue_, not just *t*_out_ (Fig. 5c). To quantify this, we fit a model where offers with different *t*_cue_ and *t*_out_ values could have different relative subjective value of information (Fig. 5d and Model 17). In all animals, the fitted value of information was highest when *t*_cue_ was early and *t*_out_ was late, that is, when *t*_advance_ was longest (Fig. 5c; all *P* < 0.001).

To quantify how individuals computed the subjective value of offers based on timing and all other attributes, we selected attributes that were required to account for ~99% of the above-chance cross-validated log likelihood of the choice data for all animals (Methods). This identified ten value-related attributes, which we then used to model both behavior and neuronal activity (Model 8). Of the ten attributes, five related to time: two for the timing and amount of juice delivery (*t*_out_ and *t*_out_ × E[r]) and three for interactions between timing and information (Info × *t*_out_, Info × *t*_advance_ and Info × *t*_advance_ × Uncertainty). Model comparisons confirmed that these Info × Time interactions improved the fit to animal behavior (Fig. 5f). Crucially, both animals from which neurons were recorded had significant positive weights of Info × *t*_advance_ and Info × *t*_advance_ × Uncertainty (Supplementary Fig. 3). Thus, the subjective value of information scaled with the time it would arrive in advance of the outcome, especially when there was a large amount of uncertainty for it to resolve.

To test if the value of information also scaled with time in humans in this setting^45,58^, we modified the human task to offer a choice between early and late access to informative cues (Fig. 5e and Supplementary Fig. 1; *n* = 210). The human population was fitted with a significant mean positive subjective value of obtaining early versus late information (Fig. 5e; *P* < 0.001; signed-rank test; Model 7), as were a substantial number of individuals (21%; above chance, *P* < 0.0001, binomial test; Supplementary Fig. 7).

Again, Pal and LHb information signals contained the necessary components to implement these value computations (Fig. 5g–n). Both areas had significant proportions of neurons with Info × Time interactions during offer presentation (Fig. 5h,l), including each of the three interactions identified from the behavioral model (Fig. 5i,m). For example, both areas contained neurons with stronger information signals when *t*_advance_ was long. Again, these Info × Time interactions improved the model fit (Fig. 5j,n and Models 8 and 14–16).

### The LHb reflects the integrated value of information and reward

**Fig. 6:**
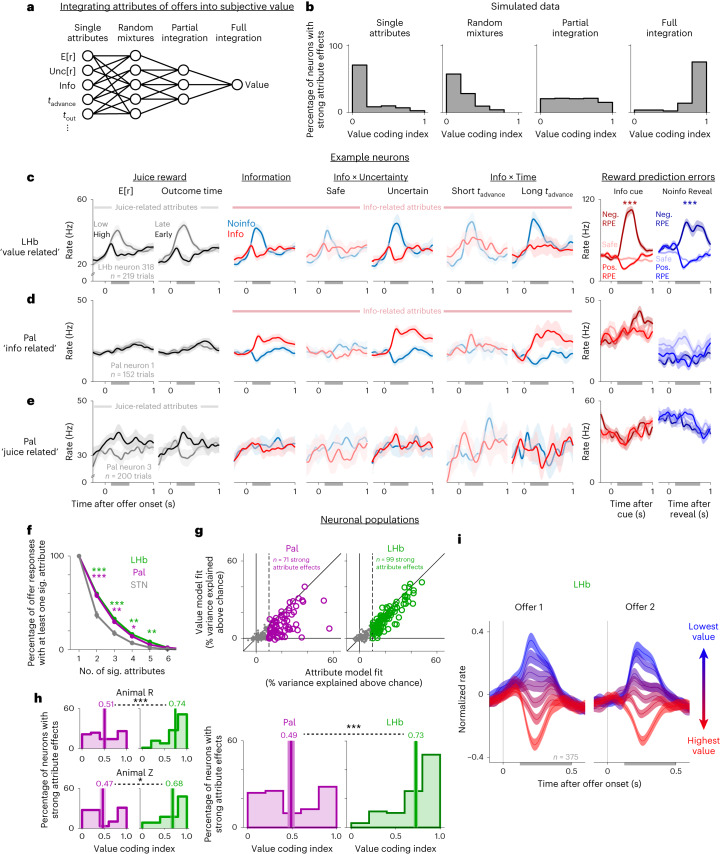
LHb neurons integrate information and reward into subjective value during multi-attribute decision-making. **a**, Hypotheses of neuronal attribute coding. Neurons could encode single attributes or random mixtures of attributes, partially integrate subsets of attributes or fully integrate all attributes to reflect subjective value. **b**, Simulations of these hypotheses produce different value coding indexes. **c**–**e**, Example neurons. Data are shown as mean ± s.e. **c**, The LHb neuron from Figs. 1–3 showing responses to attributes related to juice reward (left; E[r] and *t*_out_), information (middle; Info, Info × Unc[r] and Info × *t*_advance_; data are in the same format as in Figs. 1–3; for Info × Uncertainty and Info × Time effects, saturated color shades indicate conditions where Info versus Noinfo offers had greater differences in subjective value) and juice RPEs (right; induced by informative cues on Info trials (red) and the final reveal on Noinfo trials (blue); medium-, dark- and light-colored shades indicate positive RPEs (>50 µl), negative RPEs (<–50 µl) and safe offers with no RPE). **d**, The Pal neuron from Figs. 1–3 responds to attributes related to information (middle) but not juice (left) or RPEs (right). **e**, A second example Pal neuron responds to attributes related to juice but not information or RPEs. **f**, Attribute-responsive neurons in the LHb and Pal (green and purple, respectively) have significant effects of more attributes than in the STN (gray); *P < 0.05, **P < 0.01 and ***P < 0.001 between LHb or Pal and STN. These data include all offer responses with a significant effect of at least one attribute (treating responses to offer 1 and offer 2 separately, totaling *n* = 470, 387 and 186 such offer responses in the LHb, Pal and STN). **g**, Percent variance in the neuronal data explained above chance for each attribute-responsive Pal neuron (left, purple) and LHb neuron (right, green) when fit using the full attribute model (*x* axis) or the simple value model (*y* axis). The vertical dashed line is the threshold for classifying cells as having strong (colored circles) or weak (gray dots) attribute effects. **h**, Histograms of value coding indexes from the neurons in each area with strong attribute effects. Text and vertical lines indicate the mean, and the shaded area represents ±s.e. Left, separately for animals R (top) and Z (bottom). Right, pooled data. Comparisons between areas (dashed lines) used rank-sum tests. **i**, Population average normalized firing rate of all LHb neurons in response to offer 1 (left) and offer 2 (right) separately for each of seven bins of offer subjective values (derived from the model fit to behavior). Activity is negatively related to value. The shaded area represents ±s.e. See Supplementary Table 6 for the details of all tests and *P* values.

Thus far, we have shown that LHb and Pal neurons encode the offer attributes necessary to compute the subjective value of information. However, this does not necessarily mean that these neurons encode an offer’s total value. Value computations can have multiple stages (Fig. 6a). The many attributes of each offer must be detected, weighted and integrated to compute a value that is properly aligned with the individual’s preferences. Furthermore, both information- and reward-related attributes must be integrated together to compute the total subjective value of the offer to guide decisions. These stages should produce very different neural activity (Fig. 6b). Neurons that are ‘labeled lines’ encoding single attributes^60^ or have mixed selectivity to random subsets of attributes^61^ should generally be weakly aligned with value. Neurons that do partial integration by properly weighting a subset of attributes^60^ should generally be partially aligned with value. Finally, neurons that encode a fully integrated value signal^62^ should be closely aligned with subjective value (Fig. 6b).

Indeed, many LHb and Pal neurons encoded attributes in strikingly different ways, consistent with different stages in value computations. The LHb neuron in Fig. 6c tracked many attributes needed to compute subjective value, including attributes related to both juice reward and information. By contrast, the Pal neurons in Fig. 6d,e each encoded multiple attributes but did not integrate them into total subjective value. The first Pal neuron strongly activated for Info and activated more when other attributes scaled up the value of info (Uncertainty and *t*_advance_); however, it did not activate for attributes about juice (Fig. 6d). The second Pal neuron activated for attributes that govern the value of juice (high E[r], early *t*_out_) but not for attributes about information (Fig. 6e).

These response patterns were common in these areas. We first fit each neuron’s offer responses with the model described above and found that, on average, both LHb and Pal neurons were sensitive to similar numbers of offer attributes (Fig. 6f). We then asked whether they integrated those attributes in a manner resembling subjective value. To do this, we estimated the subjective value of each offer from each animal’s behavioral model fits, as the linear weighted combination of offer attributes that best predicted choice. We then calculated a value coding index defined as the ratio of the above-chance variance in a neuron’s activity that could be explained by a model with a single term reflecting subjective value versus a model with separate weights for each attribute (value model versus attribute model; Fig. 6g,h and Models 18 and 19). The above-chance percent variance explained for each model was defined as the percent variance explained from fitting the real data minus the percent variance explained from fitting shuffled data (Methods). Thus, an index of 1 indicates that all the above-chance variance in attribute-related activity can be explained by subjective value, whereas an index of 0 indicates that attributes are encoded orthogonally to subjective value. We analyzed all neurons with strong attribute effects, meaning that the fitted attribute model explained at least 10% more response variance than expected by chance, with at least one attribute having a significant effect (Methods). The result was clear. LHb neurons predominantly had high value coding indexes, with many close to the maximum index of 1, consistent with full integration (Fig. 6h). Pal neurons had a roughly uniform distribution of indexes, reflecting highly diverse degrees of integration, consistent with partial integration being common in Pal neurons (Fig. 6h). Thus, as a whole, LHb had greater value coding indexes than Pal (rank-sum test; *P* < 0.001; animal R, *P* < 0.001; animal Z, *P* = 0.024). Furthermore, LHb value coding indexes were much closer than Pal to the hypothesis that offer responses exclusively encoded subjective value (Supplementary Fig. 14). Indeed, even without selecting neurons based on their task responsiveness or response properties, the simple population average LHb firing rate in response to each offer closely tracked its total subjective value (Fig. 6i).

**Fig. 7:**
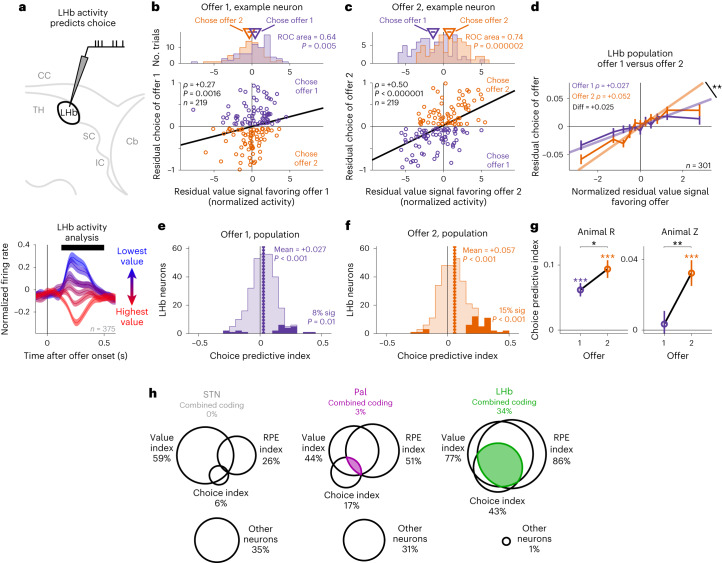
LHb value signals predict online multi-attribute decisions. **a**, Schematic of testing if LHb value-related activity is choice predictive. The black bar indicates the analysis time window. CC, corpus callosum; TH, thalamus; SC, superior colliculus; IC, inferior colliculus; Cb, cerebellum. **b**,**c**, The LHb example neuron from Figs. 1–4 had trial-to-trial variations in value-related activity during offer 1 (**b**) and especially during offer 2 (**c**) that predicted trial-to-trial variations in choice. Scatter plots show the correlation of residual neuronal value signals in response to each offer (*x* axis) versus residual choice of that offer (*y* axis). Each dot represents one trial. Colors indicate choices of offer 1 (purple) or offer 2 (orange). Lines are linear fits (type 2 regression). The text indicates rank correlation and its *P* value. Top, histograms of residual neuronal value signals for choices of offer 1 versus offer 2; the text indicates receiver operating characteristic curve (ROC) area and *P* value (rank-sum test). **d**, The LHb population average relationship between normalized residual value signals (*x* axis) and residual choice (*y* axis) had a more positive slope for offer 2. Two asterisks (**) indicate the 99% bootstrap confidence interval excluded 0. Error bars represent ±s.e. Shown are all *n* = 301 attribute-responsive LHb neurons with sufficient data from both offers for this comparison. **e**,**f**, Histograms of each neuron’s choice predictive indexes for offer 1 (**e**) and offer 2 (**f**). The dark areas indicate significant indexes (*P* < 0.05). The dashed vertical line and text indicate mean and significance of the median (signed-rank test); text in the lower right indicates the fraction of neurons with significant positive indexes and whether it is above chance (one-tailed binomial test). **g**, Both animals had higher mean choice predictive indexes for offer 2 (orange) than for offer 1 (purple); *P < 0.05; **P < 0.01; ***P < 0.001; signed-rank tests. Data are shown as mean ± s.e.; *n* = 122 and 179 for animals R and Z, respectively. **h**, Venn diagrams show, for neurons in each area with strong attribute effects, the overlap of neurons with high value coding indexes (left), significant RPE coding indexes (right) and significant choice predictive indexes (center); other neurons that met none of those criteria are shown at the bottom. Colored areas indicate neurons with combined coding of all three properties. See Supplementary Table 6 for the details of all tests and *P* values.

Our multi-attribute task design was crucial to detect this difference in coding between Pal and LHb. For example, the Pal cell in Fig. 6e would have appeared to encode total subjective value if we had only manipulated juice and not information. This suggests that the Pal contains subpopulations of neurons that partially integrate attributes, which would be suitable to motivate specific actions (for example, information- or juice-specific behaviors), whereas other Pal neurons integrate attributes more fully, suitable to regulate LHb activity and decision-making. These Pal coding properties are especially notable because they are not simply found in all basal ganglia nuclei that project to the LHb. To test this, we recorded *n* = 185 neurons from the subthalamic nucleus (STN), which has strong connections with both the Pal and LHb. The STN had strong signals related to offer attributes (Fig. 4f and Supplementary Fig. 5), but, compared to Pal, its neurons coded fewer attributes of offers (Fig. 6f) and combined fewer coding properties (Fig. 7 and Supplementary Fig. 16). Thus, our data identify Pal as a site of diverse integration of motivational attributes and the LHb as a site of predominant integration into subjective value.

### LHb value signals predict online multi-attribute decisions

We hypothesized that LHb signals tracking subjective value causally contribute to decisions. If so, this would extend the role of LHb phasic signals beyond their classic role in trial-and-error reinforcement learning to online control of multi-attribute decisions. The LHb responses to each offer in our task resembled encoding of RPEs, with each offer triggering an RPE related to its subjective value. Consistent with classic findings in the LHb, these neurons also had strong RPE signals later in the task when outcomes were cued and revealed (Fig. 6c and Supplementary Fig. 15)^25,27^. However, LHb RPEs have been implicated in reinforcement learning more than online decision-making^26,27,31,32,63,64^. If our hypothesis is correct, then variations in the LHb response to each offer should predict variations in the animal’s decision about whether to choose that offer. This relationship should hold above and beyond the predictions of our models (which are fitted to the pooled data over all trials and hence cannot predict trial-to-trial variations in subjective values). For example, if an LHb neuron treats a particular offer on a particular trial as less valuable than the neural model predicts, then the animal should be less likely to choose that offer than the behavioral model predicts. Furthermore, given the negative sign of LHb value-related signals, LHb activity should be negatively associated with choice.

To test this hypothesis, we asked whether variations in LHb value signals predict variations in choice behavior (Fig. 7a). For each neuron, we computed a choice predictive index as the correlation between the animal’s residual choice and the neuron’s residual offer response in the direction of its value signal. That is, the correlation between the choice and the neural offer value signal, after subtracting out the animal’s predicted choice and the neuron’s predicted offer value signal based on fits from the attribute model (Fig. 7b,c and Model 18). Many neurons had significant positive indexes, consistent with value signals related to choice (Fig. 7e,f). If this activity related to decision-making, it might become stronger after the animal observed both offers and could decide between them^56^. To test this, we computed the index separately for responses to the first and second offers. LHb value signals were more choice predictive during offer 2 (Fig. 7d–g).

These results suggest that the subjective value signal in LHb neurons could be well suited for several roles in motivated behavior. First, our results suggest that the value signal reflects the subjective value that governs choice and could be used to monitor or drive choice behavior. Second, the value signal could be present in conventional RPE coding neurons, in which case it could be used as an RPE signal to drive reinforcement learning of value-based behavior^25^. Indeed, LHb offer responses resembled classic LHb negatively signed encoding of RPEs^25,27^.

Therefore, we next tested whether LHb activity is organized to support these roles and whether this organization is specific to the LHb or whether it could be inherited from inputs such as the Pal. To do this, we classified LHb neurons with three indexes. We classified them as offer value related if they had high value indexes (≥0.6; Methods), as choice predictive if they had significant choice predictive indexes, and as RPE related if they had a significant RPE index, quantifying how neurons encoded RPEs in response to feedback about the trial’s reward outcome (which, in our task, came from cues on Info trials and reveals on Noinfo trials; Methods). Many LHb neurons had significant RPE indexes (Fig. 7h and Supplementary Fig. 16), including the example neuron (Fig. 6c).

We found that these coding properties were partially combined in many Pal neurons but fully combined in many LHb neurons. Consistent with partial combination, both the Pal and LHb had positive pairwise correlations between all three indexes (Supplementary Fig. 16). However, the LHb had much higher overlap between the three indexes (Fig. 7h). Only the LHb had a substantial proportion of ‘combined coding’ neurons that coded all three with the same sign (Fig. 7h). This was far above chance in the LHb (*P* < 0.0001; permutation test controlling for base rates of each coding property), but not in the Pal (*P* = 0.10), and hence was more elevated above chance in the LHb than in the Pal (*P* = 0.0025).

### The LHb causally influences online multi-attribute decisions

**Fig. 8:**
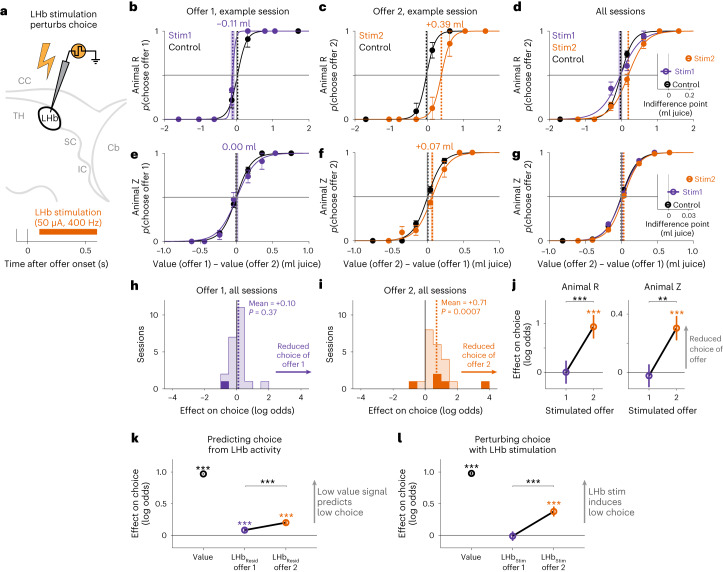
LHb stimulation causally influences online multi-attribute decisions. **a**, Schematic of testing if LHb stimulation perturbs choice. The orange bar and text indicate the stimulation time window and parameters. **b**,**c**, Psychometric curves from an example session in animal R showing how LHb stimulation during offer 1 (**b**; Stim1, purple) and offer 2 (**c**; Stim2, orange) affected the probability of choosing that offer as a function of the subjective value difference between the offers (derived from the model fit to behavior on control trials (black) and converted to units of ml of juice). Data are shown as mean ± s.e. Text and vertical dashed lines indicate the fitted indifference point. The shaded area represents ±bootstrap s.e.; *n* = 62, 24 and 31 trials for control, Stim1 and Stim2. **d**, Psychometric curves from animal R pooling data from all sessions showing how stimulation influenced choice of offer 2 relative to control trials. Data are shown as mean ± s.e.; *n* = 537, 216 and 263 for control, Stim1 and Stim2. The inset shows indifference points for each condition. Data are shown as mean ± s.e. **e**–**g**, Same as in **b**–**d** but for animal Z; *n* = 517, 132 and 141 for **e** and **f**; *n* = 5,088, 1,418 and 1,316 for **g**. **h**,**i**, Histograms of per session GLM fitted effects of stimulation during offer 1 (**h**) and offer 2 (**i**) on choice of that offer. Positive effects indicate reduced log odds of choice. The dark areas are significant (*P* < 0.05, *t*-test). The dashed vertical lines and text indicate the mean effect and significance of the median (signed-rank test). **j**, Fitted main effect of stimulation on choice in each animal, pooling data over all sessions. Positive effects indicate reduced log odds of choice. Data are fitted parameters ± s.e.; **P < 0.01; ***P < 0.001; *t*-tests. **k**, Trial-to-trial variations in LHb activity aid in predicting choices. Shown are fitted weights from a GLM predicting choices based on the model-derived subjective values of the offers (value, black) and residual (Resid) normalized value signals from LHb responses to offer 1 (purple) and offer 2 (orange). Data are shown as fitted parameters ± s.e.; ***P < 0.001. **l**, LHb stimulation (Stim) perturbs choices. Shown are fitted weights from a GLM predicting choices based on the model-derived subjective values of the offers (value, black) and LHb stimulation during offer 1 (purple) and offer 2 (orange). Positive effects of stim indicate the hypothesized effect, reduced log odds of choice. Data are shown as fitted parameters ± s.e. LHb stimulation during offer 2 significantly reduces choice and does so significantly more than stimulation during offer 1. See Supplementary Table 6 for the details of all tests and *P* values.

To test whether the LHb causally influences online decisions, we manipulated LHb activity using weak electrical stimulation at the same time LHb neurons signaled offer value (Fig. 8a; *n* = 2 animals; 50 µA, 400 Hz, 500 ms). On each trial, we stimulated during the LHb response to offer 1 or offer 2 or did not stimulate (Methods). If our hypothesis is correct, then adding spikes to the LHb response to an offer should cause the animal to treat that offer as if it had less subjective value and hence choose it less. Furthermore, stimulation during offer 2 might have a greater influence on choice, both because LHb activity was more choice predictive during offer 2, and because animals typically made their choice shortly after offer 2 was presented and hence had less time to review and re-evaluate the options after offer 2 stimulation.

Indeed, LHb stimulation influenced decisions (Fig. 8). This could be seen as a shift in the psychometric curves relating estimated offer values to choice. Both animals were less likely to choose the stimulated offer, particularly when stimulation occurred during offer 2 (Fig. 8b–g). To quantify this, we extended our behavioral model to include additional attributes indicating the presence of stimulation during each offer (Stim1 and Stim2). Stimulation had a significant effect on choice, which was more potent during offer 2 (Fig. 8h–j; Stim2 *P* = 0.000003, Stim1 *P* = 0.98, Stim2 > Stim1 *P* = 0.0001). This occurred consistently across sessions (Fig. 8h,i) and animals (Fig. 8j). The fitted model parameters are consistent with LHb stimulation during offer 2 leading animals to treat that offer as if it had less subjective value, equivalent to a reduction in its expected yield of juice by 0.164 ml in animal R and 0.036 ml in animal Z. Control analysis showed that stimulation effects on choice were not caused by directly evoking motor actions, such as choice reports (Supplementary Fig. 17), and could be modeled as subtracting a fixed amount of value from the offer (Supplementary Table 4 and Models 23–27).

Finally, we asked if we could use LHb activity to predict choices by augmenting our behavioral models with terms representing either natural variations in LHb residual activity (Fig. 8k and Models 20 and 21) or stimulation-induced variations in LHb activity (Fig. 8l and Model 22). The fits were similar. Both natural and artificial variations in LHb activity were choice predictive and more so during offer 2 (Fig. 8k,l).

## Discussion

We found that humans and monkeys value information about uncertain future outcomes and integrate it with the value of extrinsic rewards through remarkably conserved computations, even to the extent of measuring uncertainty with similar functional forms and using it to motivate information seeking in similar manners. Furthermore, we find that these conserved value computations are propagated in an evolutionarily conserved ancient epithalamic structure, the LHb. The LHb contains neurons tracking the total subjective value of offers, integrating both informational and primary reward values, and causally influences online decisions. By contrast, many neurons in major basal ganglia inputs to the LHb, anterior/ventral Pal and STN commonly encode attributes or partially integrated values rather than fully integrated, total subjective values that could drive multi-attribute decisions.

The LHb is an ancient structure conserved in even the most primitive extant vertebrates^65^. It has been implicated in primary reinforcement in diverse species, including fish, rats and primates^25,33,66^. Our work shows that the LHb also has a key role in the sophisticated motivational value computations that primates use for multi-attribute decisions, including economic judgements trading off extrinsic rewards against the intrinsic reward of gaining information.

Our findings shed new light on the Pal–LHb pathway in value computations and decisions. Neurons in both areas are reported to respond to stimulus attributes needed to compute subjective value^27,34,35,37^ and to encode RPEs^27,38,67^, suggesting that Pal neurons could motivate behavior by directly providing LHb neurons with value and RPE-related signals. Here, we show in primates that Pal has the necessary signals to accomplish this but primarily at the population level. Our multi-attribute task revealed that many single Pal neurons only partially integrated offer attributes, and very few combined strong value signals with the choice-predictive and RPE-related activity that were common in LHb neurons. Instead, our data indicate that the Pal contains a diverse repertoire of neurons resembling intermediate stages of value computations. These Pal subpopulations could be suitable to motivate attribute-specific forms of behavior tailored to attributes of juice (for example, thirst^38^), information (for example, curiosity and gaze shifts^23^) or other incentives, such as risk^36^. This organization may explain why Pal is implicated in such diverse forms of motivated behavior^34^. These subpopulations could then be further integrated to compute the subjective value signals in LHb to motivate total value-guided behavior. It is also possible that the minority of Pal neurons with the most value- and RPE-like activity could have a predominant role in regulating LHb activity or that, in spite of the mixed coding schemes in the Pal, stimulation of Pal and LHb could produce similar effects on behavior. In either case, our data are consistent with these areas performing different steps of subjective value and RPE computations.

Our data implicate the LHb in the subjective valuation of options that drives online decisions. Although it was established early on that LHb responses are related to reward and punishment^27,28^, there are multiple motivational systems in the brain that track similar motivational variables for radically different functions (learning versus online decisions, different learning rules, different decision rules, etc.^68,69^). The LHb has been most implicated as a source of teaching signals in a reinforcement learning system, including dopamine and the basal ganglia^25,30,33^. Yet, in many contemporary theories, the ‘value’ in these teaching signals is quite different from the subjective value that drives online decisions^70^. Further, although stimulation of the LHb is known to strongly influence behavior, this is often a slow change over trials indicative of a learning process^31,32,71,72^ or a general aversion to stimulated contexts^32^. There are less data on the role of LHb activity during decisions^26,63,71,73–75^. Here, we demonstrate converging evidence that LHb neurons reflect value and influence online decisions: (1) LHb offer responses were linked to subjective value, (2) variations in these value signals were choice predictive, (3) LHb stimulation altered decisions as if reducing the stimulated offer’s value, and (4) both choice predictiveness and causal influence were greatest after both offers were presented and animals could decide between them. Thus, the LHb does not simply signal RPEs for adjusting estimated values over long timescales during reinforcement learning; it also influences ongoing decisions.

An important question for future work will be uncovering the mechanism and downstream pathways by which LHb stimulation alters online decisions. Past work indicated that LHb responses are too slow to govern choices during simple rapid decisions (immediate saccades to single-attribute options^26^). Our data indicate that LHb does influence more prolonged, complex, multi-attribute decisions. The LHb has potent control over dopamine and serotonin neurons that could influence decisions on this timescale^25^. The LHb could also reduce choice of the stimulated option via projections to areas implicated in aversive behaviors^74,76–78^. A caveat is that LHb stimulation could induce spikes in neuronal fibers in the LHb rather than solely in LHb neurons themselves (although we used a protocol intended to minimize current spread and off-target activation of fibers of passage, as suggested by previous work^79–82^).

Our data also have important implications for a fundamental question in neuroscience: what form of uncertainty motivates behavior and how? Uncertainty regulates many aspects of cognition, motivation and behavior^83^. However, it is rarely possible to test between proposed uncertainty measures in conventional tasks that manipulate uncertainty with a single parameter (Supplementary Fig. 11). Here, we show that, in our task, humans and monkeys scale up the value of information with a specific family of uncertainty measures, resembling the SD or variance of rewards. This is consistent across species and individuals with diverse attitudes toward risk. Further, Pal and LHb information signals closely track this value of information. This places important constraints on the underlying neural computations, indicating that the brain computes the value of information by tracking a large suite of the reward statistics that produce uncertainty in natural environments. Of course, organisms may adapt their value computations to the task at hand. In some tasks, they may use a different uncertainty measure if it is easier to compute or provides an instrumental benefit^51^. Our key finding is that, when confronted with analogous tasks, humans and monkeys valued information in analogous manners. These value computations were surprisingly robust to their different behavioral regimes. Humans learned the task from written instructions and chose with mouse clicks for monetary rewards in a single session sitting at their own computer, whereas monkeys learned from experience over many sessions and chose with eye movements for juice rewards in an experimental booth. Thus, these conserved computations to endow information with value may generalize across a range of natural and experimental environments.

Finally, our work shows that a neuroscience approach using both humans and monkeys can identify conserved computations underlying complex multi-attribute decision-making and tie them to neuronal substrates. Work in both humans and monkeys has been crucial for understanding movement disorders and developing neuroscientific treatments^84^, and the same is likely to be true for disorders of mood and cognition, which impair complex decision-making and often include maladaptive information-seeking strategies^85,86^.

The LHb is implicated in human disorders, including depression, schizophrenia and substance abuse, and in animal models^33,87,88^. Interventions targeting the LHb are being evaluated for human treatments^89,90^. Our work may explain why alterations in the LHb could have broad effects on mood and motivation in everyday life. Its highly integrated code means that it could be engaged by diverse motivational goals. Further, LHb signals can fulfill two complementary functions simultaneously: (1) a teaching signal to learn which environments produce valuable outcomes and (2) an immediate motivational push to enter those valuable environments. These functions can support each other; a high-quality teaching signal requires access to informative cues, and an immediate motivational push to seek information would help obtain them^6,10^. Several forms of information seeking are altered in individuals with traits or disorders affecting mood and cognition^91–93^, including diseases or impairments of the dopamine and serotonin neuromodulator systems that the LHb potently regulates^94–97^. Thus, our work raises the possibility that disordered LHb signals produce mood alterations due to not only impairing an individual’s motivation for extrinsic rewards but also sapping their motivation to seek information from their environment.

## Methods

### General procedures

All human procedures were approved by the Washington University Institutional Review Board. All animal procedures conformed to the Guide for the Care and Use of Laboratory Animals and were approved by the Washington University Institutional Animal Care and Use Committee. In total, 824 human participants completed tasks using Amazon’s Mechanical Turk service (mean age = 36.06 years, s.d. of age = 11.15 years; 410 females). All provided informed consent. Participants were monetarily compensated based on their performance, as detailed below. Participants were required to be healthy adults between the ages of 18 and 55 years with no history of previous neurological or psychiatric illnesses, located in the United States and able to read English and have normal or corrected-to-normal visual acuity. Four adult male rhesus monkeys (*Macaca mulatta*) participated in the experiments (animals R, Z, B and P; all 7–9 years old during the experiments). Data collection and analysis were not performed blind to the conditions of the experiments. No statistical methods were used to predetermine sample sizes, but our sample sizes are similar to or exceed those reported in previous publications^23,26^.

### Behavioral task for humans

The main multi-attribute information choice task for humans involved making choices between offers, which provided opportunities to earn virtual coins representing monetary rewards (*n* = 580 participants; self-reported mean age = 36.20 years; s.d. of age = 11.06 years; 294 female, 282 male, 2 other and 2 no response). These participants earned an average of $9.81 (s.d. = $0.41) from the entire task. We analyzed data from all participants whose behavior had enough variability for the model described below to converge to a valid fit. This excluded participants who followed a simple deterministic strategy like always choosing the leftmost offer (although results were very similar if analyzing all participants). This produced a dataset of *n* = 565 participants. Participants learned the task by reading written instructions at their own pace and completing ten practice trials before beginning the actual task. The task was programmed using psiturk v3.1.0 and jspsych v6.0.4.

The task had 150 trials in three blocks of 50 trials each. Participants had 45 min to complete all trials and 10 min of break time that they could take as they wished between blocks. Each trial began with a central cross that the participant had to click. Two offers then appeared on the left and right. The participant had 5 s to choose an offer by clicking it. If they did not choose, the computer randomly chose an offer for them; this occurred very rarely (0.88% of trials), and results were very similar if those trials were excluded from analysis. After choice, the unchosen offer disappeared, and the chosen offer displayed an animation for ~3.7 s (Supplementary Fig. 1). The participant then had to click the offer to complete the trial.

Each offer’s reward distribution was depicted with four coin stacks. After an offer was chosen, one of its four stacks was selected uniformly at random as the outcome, and the number of coins in that stack was added to the participant’s total winnings. Each reward distribution was defined by its expected reward and type of uncertainty. The expected reward could be 5, 6 or 7 coins. Type of uncertainty could be safe (all stacks were the same), 25/50/25 (one stack was four coins below the mean, two were the mean, and one was four coins above the mean) or 50/50 (two stacks were four coins below the mean and two stacks were four coins above the mean). Each offer could be informative or non-informative, as indicated by its color (blue or orange). Informative offer animations ended by highlighting the outcome stack and displaying text indicating its amount (for example, ‘You won 7 coins!’). Non-informative offer animations ended without highlighting any stacks and displaying non-informative text (‘You won ? coins!’). The initial mapping between color and informativeness was randomized for each participant and reversed approximately halfway through the task (block 2, trial 20). Participants were instructed that the information was non-instrumental and did not affect their payment in any way and that this reversal would occur at some point during the task.

Offer pairs were generated randomly for each trial and participant. In 75% of trials, each offer’s features (expected reward, uncertainty type and informativeness) were selected independently and uniformly at random across each feature’s possible values. In the remaining 25% of trials, offer features were selected so one offer was informative and the other was non-informative, but both offers had the same expected reward and uncertainty type (selected independently and uniformly at random). We combined data from three slightly different task versions, which produced similar results. The first version instructions referred to 25/50/25 and 50/50 offers as ‘moderate risk’ and ‘high risk’; other version instructions referred to both as ‘risky’. The first version placed the informative offer on the right on the 25% of trials where offers were generated with the same reward distribution but different informativeness; in the other version, offer positions were always randomized.

To test how participants understood the reversal, we included additional ‘question trials’ (two per block after trials 30 and 50). Participants were shown pictures of two offers, one informative and one non-informative, and were asked to click on the informative offer on half of question trials and the non-informative offer on the other half. Each correct response earned $0.10. The majority of participants answered most question trials successfully; restricting analysis based on question trial performance produced similar results.

A second task for humans manipulated information timing (*n* = 244 participants who earned an average of $6.45 (s.d. = $0.45) from the entire task; *n* = 210 with valid model fits; self-reported mean age = 35.73 years, s.d. of age = 11.37 years, 116 female, 124 male, 2 other and 2 no response; no overlap of participants between the two tasks). The task was similar, with the following differences (Supplementary Fig. 1). There were two blocks of 50 trials each, for a total of 100 trials. All offers were 50/50. The animation was longer (~14 s). Instead of offer colors indicating informative versus non-informative, they indicated early information versus late information (EarlyInfo versus LateInfo). EarlyInfo animations highlighted the outcome stack at the start, revealing the outcome immediately. LateInfo animations highlighted the outcome stack near the end, revealing the outcome after an approximately 12-s delay. All animations ended with informative text (for example, ‘You won 7 coins!’). Of the 25% of trials in which the offers were generated with the same offer distribution but different informativeness, expected reward was generated randomly on *n* = 1 trial and deterministically on *n* = 24 trials (8 for each of the three possible expected reward values). Question trials occurred after trials 10 and 40 in each block.

After each experiment, we asked participants to complete a brief questionnaire to report their age and gender and answer questions about their understanding of the task (the meanings of coin stacks, cues, reward amounts and probabilities, offer colors and so on). Participants were told that they were not required to answer any questions, and their answers would not affect their payment, but almost all participants answered all questions. Reported age or gender had no significant effects on the main behavioral effects (Info × Uncertainty in the first task and EarlyInfo in the second task; Supplementary Fig. 18). One key question tested if participants understood that the offer’s color indicated its informativeness: ‘What did the color of the option mean?’. The correct response was phrased slightly differently for different participants. In the first version of the task, it was ‘Whether the option’s lights will indicate the outcome’ (*n* = 179), ‘Whether the outcome will be revealed at the end of the trial’ (*n* = 105) or ‘When the outcome will be revealed’ (*n* = 286). In the second version of the task, it was ‘When the outcome will be revealed’. In all versions, there were four possible incorrect responses: ‘Number of coins awarded from that option’, ‘Probability of winning coins if that option is chosen’, ‘Length of the trial’ and ‘None of the above’.

### Behavioral task for monkeys

The multi-attribute information choice task for monkeys had two versions. The first verison had choices between offers for juice rewards with fixed event timing (animals R, Z, B and P). The second version augmented offers to allow different event timings (animals R, Z and B). Recording and stimulation experiments were performed in the second version in two animals (animals R and Z).

In the first version of the task, each trial began with the appearance of a white fixation point at the center of the screen, which the animal was required to fixate on with its gaze. The task proceeded once the animal maintained fixation continuously for 0.25 s and at least 0.5 s had passed since fixation point onset. If the animal did not fixate within 5 s or broke fixation before 0.5 s had passed since fixation point onset, the trial was an error; it moved immediately to the intertrial interval and repeated until it was performed successfully. After the fixation requirement was completed, the fixation point disappeared, and there was no longer any gaze requirement to advance the task. Simultaneously with fixation point disappearance, the first offer appeared on the screen. After 1 s, the second offer appeared on the screen. After a further 0.5 s, the choice period began. An offer was counted as chosen once the animal held its gaze on it continuously during the choice period for a fixed duration (0.5 s in animals R, B and P and 0.4 s in animal Z). If the animal did not choose an offer within 5 s, the computer randomly selected an offer and the trial proceeded as if the animal had chosen it. This occurred rarely, and these computer-chosen trials and error trials were excluded from analysis unless otherwise noted. Each offer stimulus was ~6° × 5° of visual angle. Offer locations were randomly selected each trial from a set of three possible locations (center, 10° left of center and 10° right of center).

Each offer’s reward distribution was depicted with four bars, with bar height proportional to juice reward volume, up to a maximum size *R*_max_ corresponding to the maximum bar height (Supplementary Table 1). If an offer was chosen, one of its bars was selected uniformly at random as the juice outcome to be delivered that trial. Juice was delivered through a metal spout placed directly in the mouth to ensure that anticipatory actions, such as licking, were not required and did not influence the amount of reward received. Each offer could be informative or non-informative, as indicated by distinct visual textures on its bars (we used complex textures sourced from images of scenes; for clarity of presentation, these are depicted in the figures as simple colors (shades of red, blue and white)). The mapping from texture to informativeness was counterbalanced across animals. Informative offers presented a visual cue highlighting the outcome bar. Non-informative offers presented the same cue at the same time but highlighting a random one of the four bars. Thus, choosing an informative offer gave early information about the upcoming outcome.

After the choice, the unchosen offer disappeared, and after 0.5 s, the visual cue appeared on the chosen offer in the form of a black box highlighting a bar. After a further 3.5 s, a ‘reveal’ occurred where the three non-outcome bars disappeared, and only the outcome bar remained. After a further 0.5 s, the reward was delivered. Thus, the reveal always provided full information about the upcoming outcome. This ensured that animals always had adequate time to physically prepare to consume the reward on all trials. Thus, in this task, *t*_cue_ (the time of the cue after the choice) was 0.5 s, *t*_reveal_ (the time of the reveal after the choice) was 4.0 s, and *t*_advance_ (the time the cue appeared in advance of the reveal) was 3.5 s. Finally, the offer disappeared 0.5 s after outcome onset, then a 1.2-s intertrial interval occurred before the next trial.

Offers could have multiple types of reward distributions. For full details, see Supplementary Table 1. In brief, when randomly generating an offer, the offer first had its expected reward drawn uniformly at random from a prespecified set of possible expected rewards. To reduce the number of trials with trivial decisions where one offer was much better than the other, the expected reward of offer 2 was constrained to be within a prespecified range of the expected reward of offer 1 for some animals and sessions. Then, the offer had its reward distribution randomly drawn from the following types: safe distributions with 100% probability of a specific amount; 25/50/25 distributions with 25, 50 and 25% chances of small, medium and large amounts, where medium was the mean of small and large; 50/50 distributions with 50% chances of small or large amounts and, in some animals and sessions, 25/75 distributions with a 25% chance of one amount and a 75% chance of a different amount. After the offer’s distribution type was selected, its reward range was randomly drawn from a prespecified set of ranges. Finally, the offer’s four possible reward amounts were discretized to occur in increments of *R*_step_ between 0 and *R*_max_. For example, in animal R, *R*_step_ = 0.02 and *R*_max_ = 0.6, so there were 31 possible reward amounts (0, 0.02,…, 0.6 ml).

In the second version of the task (Supplementary Fig. 2), we manipulated information and reward timing. Each offer was augmented with a ‘clock’: a horizontal bar at the bottom of the offer. The horizontal extent of the clock represented the full time duration between choice and the end of the trial (always equal to 8 s). The clock was divided into three sequential segments, representing the times (1) from choice to cue, (2) from cue to reveal and (3) from reveal to the end of the trial. The reward delivery time was not explicitly indicated but was always 1.1 s after the reveal. The three segments had distinct visual textures, which were also distinct for informative versus non-informative offers (again, for presentation clarity, these are depicted in figures as shades of red and blue). Thick black vertical lines indicated *t*_cue_ and *t*_reveal_. Finally, to help animals anticipate event times and learn how the clock represented times, we animated the clock (Supplementary Fig. 2). After choice, a ‘hand of the clock’ appeared. It was a black rectangle whose position on the clock indicated the current time. The hand moved from left to right at a constant speed. When the hand touched the vertical line indicating *t*_cue_, a 0.6-s animation played where the vertical line moved upward to the cued bar then expanded to become the cue. When the hand touched the second vertical line indicating *t*_reveal_, a 0.6-s animation played where the vertical line moved upward to the chosen offer and ‘erased’ the three non-outcome bars, leaving only the outcome bar remaining. When the hand touched the right edge of the clock, the offer disappeared, and the intertrial interval began.

For each offer, the pair of event times (*t*_cue_ and *t*_reveal_) was drawn uniformly at random from the set of all possible pairs meeting these requirements: *t*_cue_ between 0.4 and 5.8 s, *t*_reveal_ between 1.2 and 6.7 s, *t*_cue_ at least 0.8 s before *t*_reveal_ and *t*_cue_ and *t*_reveal_ both multiples of 0.1 s. We also altered the statistics of the reward distributions. For full details, see Supplementary Table 1. There were two major changes. First, because this task version had longer trials, we scaled up *R*_max_ to maintain a high reward rate to keep animals motivated. Second, we used more limited reward distributions to make it easier for animals to interpret them along with the added visual and motivational complexity from the clock.

During recordings, the above task was interleaved in alternating blocks with a simpler information anticipation task used previously^23^. The multi-attribute choice task had a block of 30 correctly performed trials; then the information anticipation task had a block of 9 correctly performed trials. The information anticipation task began with a large purple fixation point at the center of the screen, which the animal was required to fixate on with its gaze. Importantly, the fixation points for the multi-attribute choice task and the anticipation task were visually distinct, so animals could know which task was in effect. After fixation, a visual fractal conditioned stimulus (CS) was presented for 1.5 s and replaced by a visual fractal cue for 1.5 s. The cue then disappeared simultaneously with outcome delivery, followed by a 1.6-s intertrial interval. The CS location on screen was randomly selected each trial from the same set of locations from the multi-attribute choice task. There were three unique CSs: safe CS, risky CS and info CS. These CSs all yielded exactly the same expected reward volume. Each CS had a unique set of two possible cues, one of which was randomly selected for each trial. The safe CS had two cues, each yielding 100% probability of a medium-sized reward. The risky CS had two cues, each yielding 50% large reward (two times the medium reward) and 50% no reward. The Info CS had one cue yielding 100% large reward and one cue yielding no reward. Each nine-trial block had three presentations of each CS in a random order.

### Data acquisition

We recorded neurons in LHb, anterior/ventral regions of Pal, and STN. A plastic head holder and plastic recording chamber were fixed to the skull under general anesthesia and sterile surgical conditions. The chambers were tilted laterally by 35–40° and aimed to access the areas of interest. After animals recovered from surgery, they participated in the experiments. Electrode trajectories were determined with a 1-mm spacing grid system and with the aid of magnetic resonance images (3T) obtained along the direction of the recording chamber. This magnetic resonance imaging (MRI)-based estimation of neuron recording locations was aided by custom-built software (PyElectrode v0.3.0 (ref. ^99^)). Also, to further verify the location of recording sites, after a subset of experiments, the electrode was temporarily fixed in place at the recording site, and the electrode tip’s location in the target area was verified by MRI (Supplementary Fig. 19).

Electrophysiological recordings were performed using multicontact electrodes (Plexon V-probes, 32 channels, 50-μm spacing) inserted through a stainless steel guide tube and advanced by an oil-driven micromanipulator (MO-97A, Narishige). Signal acquisition (including amplification and filtering) was performed using an OmniPlex 40-kHz recording system (Plexon). Spike sorting was performed offline using publicly available software (Kilosort2) to extract clusters from the recordings and manual curation to identify sets of clusters that corresponded to single neurons, the spans of time when they were well isolated and whether they were located in the regions of interest. Regions of interest were identified based on electrophysiological characteristics, firing patterns and locations relative to anatomical landmarks estimated from MRI and recordings at multiple grid locations. LHb neurons were also identified by having recording depths within ~0.5 mm of neurons with negatively signed reward-related activity in response to cues, reveals and/or reward delivery. A subset of Pal neurons (*n* = 30) were recorded using single-contact glass-coated electrodes (Alpha Omega) or epoxy-coated electrodes (FHC), from which spikes were sorted offline (Plexon Offline Sorter). Neuronal and behavioral analyses were conducted offline in MATLAB (Mathworks). In total, *n* = 375 LHb neurons, *n* = 294 Pal neurons and *n* = 185 STN neurons were recorded. The following are the statistics of the trial counts per neuron: LHb: mean 205.2, median 204, s.d. 82.6 and range 80–423; Pal: mean 187.6, median 163, s.d. 89.8 and range 82–436; STN: mean 143.4, median 130, s.d. 58.7 and range 43–330.

Eye position was obtained with an infrared video camera (Eyelink, SR Research). Behavioral events and visual stimuli were controlled by MATLAB (Mathworks) with Psychophysics Toolbox extensions. The juice reward was delivered with a solenoid delivery reward system (CRIST Instruments).

### Electrical stimulation

During electrical stimulation sessions (*n* = 10 in animal R and *n* = 12 in animal Z), low-intensity electrical stimulation (50 μA, 400 Hz, 500 ms, biphasic negative-positive pulses, 200 μs per phase) was delivered to the LHb in a subset of trials. Stimulation strength was chosen based on previous monkey studies^79,80,100,101^. Stimulation was delivered in a postoffer time window starting 100 ms after the onset of one of the offers. Specifically, in animal R, stimulation occurred after offer 1 in 25% of trials and after offer 2 in 25% of trials, and there was no stimulation in the remaining 50% of trials. In animal Z, stimulation occurred after offer 1 in 17% of trials and after offer 2 in 17% of trials, and there was no offer period stimulation on the remaining 66% of trials. In a subset of trials in animal Z with no offer period stimulation, stimulation occurred after the choice in a precue or prereveal time window, ending simultaneously with cue or reveal onset, respectively, each occurring in approximately 12.5% of total trials. In animal R, the information anticipation task was not run during stimulation experiments. In animal Z, the information anticipation task was interleaved with the main task and included stimulation in 25% of trials, which was equally likely to occur at one of three times: post-CS starting 100 ms after CS onset, precue ending simultaneously with cue onset or preoutcome ending simultaneously with outcome delivery.

### Data analysis

Unless otherwise noted, all statistical tests were two tailed, and statistical significance corresponds to *P* < 0.05 without adjustment for multiple comparisons. The notations *, ** and *** in figures correspond to *P* values of <0.05, <0.01 and <0.001 (or 95%, 99% and 99.9% confidence intervals excluding 0), respectively. Neural firing rates in response to each offer were analyzed in a time window 125–500 ms after offer onset. Firing rates for each neuron were converted to normalized firing rates by *z* scoring. Specifically, for each neuron, we computed a vector of firing rates from all times during all trials in non-overlapping 500-ms bins. We then normalized the neuron’s firing rates in our analyses by subtracting the mean of that vector and dividing by the s.d. of that vector. A neuron was classified as attribute responsive if the main GLM used to analyze neuronal activity (described below), when fit to its responses to either offer 1 or offer 2, yielded a significant effect of at least one attribute (*P* < 0.05).

#### Psychometric analysis of the value of information

For each individual, we plotted the choice of Info as a function of the difference in E[r] between the Info and Noinfo offers using only trials where the offers differed in informativeness. We fit the underlying single-trial data with a logistic function (using a GLM for binomial data with a logistic link function). We then estimated the subjective value of information as the function’s indifference point (the *x* coordinate where the function produces 50% choice of info) multiplied by −1 and estimated its standard error by bootstrapping over trials (*n* = 200 bootstraps). To estimate the mean subjective value of information for the human population as a whole, we did the same procedure except fitted the population average of the choice data from the individuals, fitted the logistic function by minimizing the squared error and bootstrapped over individuals (*n* = 200 bootstraps). To estimate the effect of uncertainty, we performed the same analysis separately for the subsets of trials where both offers were high uncertainty or both offers were low uncertainty (humans: uncertain offers versus certain offers; monkeys: offers with SD[r] greater than or less than the median SD[r] of offers presented to that animal).

#### Modeling framework

We fit each individual’s binary choice data using GLMs designed to model a standard decision-making formulation in which values are computed for each offer, and the resulting choice probability is a logistic function of their value difference:

$$\log \Big(\Big.\frac{p\left({\mathrm{choose}}\,{\mathrm{offer }}\,2\right)}{p\left({\mathrm{choose}}\,{\mathrm{offer }}\,1\right)}\Big)=V\left({\mathrm{offer }}\,2\right)-V({\mathrm{offer }}\,1)$$

and the value of each offer *i* is a linear weighted combination of the offer’s vector of *n* attributes $< {x}_{i,1},{x}_{i,2},\ldots ,{x}_{i,n} >$:

$$V\left({\rm{offer }}\,i\right)={\beta }_{1}{x}_{i,1}+{\beta }_{2}{x}_{i,2}+\ldots +{\beta }_{n}{x}_{i,n}$$

Thus, the resulting model was a GLM for binomial data with a logistic link function, with the equation

$$\begin{array}{l}\log \Big(\Big.\frac{p\left({\rm{choose}}\,{\rm{offer }}\,2\right)}{p\left({\rm{choose}}\,{\rm{offer }}\,1\right)}\Big)={\beta }_{1}\left({x}_{2,1}-{x}_{1,1}\right)+{\beta }_{2}\left({x}_{2,2}-{x}_{1,2}\right)\\+\ldots +{\beta }_{n}\left({x}_{2,n}-{x}_{1,n}\right)\end{array}$$

Note that the binary choice data satisfied this GLM’s assumption of binomial data. For neuronal data, we fit firing rates in response to the offers, with an analogous GLM for normal data with an identity link function (equivalent to ordinary linear regression):

$${{\mathrm{Rate}}}\left({\rm{offer }}\,i\right)={\beta }_{i,0}+{\beta }_{i,1}{x}_{i,1}+{\beta }_{i,2}{x}_{i,2}+\ldots +{\beta }_{i,n}{x}_{i,n}+\varepsilon$$

where ${\beta }_{i,0}$ is the neuron’s mean or baseline response to offer *i*, and *ε* is the error term. We separately fitted each neuron’s responses to offer 1 and offer 2. Note that this model was chosen for simplicity of fitting and interpretation so that weights represent the simple linear effect of each attribute. This model is optimal if true effects are linear and noise is normal but is likely to be suboptimal for firing rate data for which these were not formally tested and may not hold.

For each analysis, we used models with attributes tailored to the behavioral task and question at hand. For full details, see Supplementary Modeling Note and Supplementary Tables 2 and 3. Below is a brief summary of the model-based analysis including model comparison, peristimulus activity, value coding index, simulated datasets and choice-predictive activity.

#### Model comparison

We compared models with shuffle-corrected log likelihoods to correct for the fact that some models had different numbers of parameters. Model interpretations were aided by testing if key parameters were significant or were significantly different from each other (using the linhyptest function in MATLAB). For the first version of the human and monkey tasks (Figs. 2–4), we compared models that were identical except for whether their Uncertainty terms were set equal to SD, range or entropy:

$${{\mathrm{SD}}}\left[r\right]={\left(\sum _{r}p\left(r\right){\left(r-E\left[r\right]\right)}^{2}\right)}^{0.5}$$

$${{\mathrm{Range}}}\left[r\right]=\max \left(r\right)-\min \left(r\right)$$

$${{\mathrm{Entropy}}}\left[r\right]=-\sum _{r}p\left(r\right){\log }_{2}(p\left(r\right))$$

We also fit more detailed models with separate attributes to measure the effect of each uncertain offer type (25/50/25 versus 50/50). For the second version of the tasks (Figs. 3–8) that manipulated event timing, we used models with attributes for key timing parameters and interactions with information, including Info × *t*_out_, Info × *t*_advance_ and Info × *t*_advance_ × Uncertainty. In several analyses, we used model fits to derive an estimate of the subjective value that an individual assigned to each offer presented on each trial (in units of log odds of choice) by simply plugging the fitted weights $({\beta }_{i,1},{\beta }_{i,2},\ldots ,{\beta }_{i,n})$ and offer attributes $({x}_{i,1},{x}_{i,2},\ldots ,{x}_{i,n})$ into the equation for $V({\rm{offer }}\,i)$ above.

#### Peristimulus activity

For uncertainty activity, we defined measures of the effects of Info × Uncertainty (Fig. 3) or Info × Uncertainty Type (Fig. 4) on activity over time separately for neural responses to each offer

$$\begin{array}{l}{\mathrm{InfoxUnc}}=\left[{{\mathrm{Rate}}}\left({\mathrm{Info}},\,{\mathrm{Uncertain}}\right)-{{\mathrm{Rate}}}\left({\mathrm{NoInfo}},\,{\mathrm{Uncertain}}\right)\right]\\-\left[{{\mathrm{Rate}}}\left({\mathrm{Info}},{\mathrm{Certain}}\right)-{{\mathrm{Rate}}}\left({\mathrm{NoInfo}},{\mathrm{Certain}}\right)\right]\end{array}$$

$$\begin{array}{l}{\mathrm{InfoxUncType}}=\left[{{\mathrm{Rate}}}\left({\mathrm{Info}},50/50\right)-{{\mathrm{Rate}}}\left({\mathrm{NoInfo}},50/50\right)\right]\\-[{{\mathrm{Rate}}}\left({\mathrm{Info}},25/50/25\right)-{{\mathrm{Rate}}}({\mathrm{NoInfo}},25/50/25)]\end{array}$$

and sign normalized them so that both negative and positive modulations of activity resulted in positive effects. We then selected neural responses with significant effects and plotted their mean effects over time. We used cross-validation across offers to control for selection bias. For each neuron, we decided whether to include data from its offer 1 response and whether to treat its offer 1 response as having a positive or negative sign solely based on the model fit to its offer 2 response (and vice versa). Thus, under the null hypothesis that the neuron had no true effect, there would be no cross-validated activity (confirmed by simulations; Supplementary Fig. 10). We used an analogous procedure for peristimulus time activity (Fig. 5) but pooled the effects of the three info- and time-related attributes in that model (Info × *t*_out_, Info × *t*_advance_, Info × *t*_advance_ × Uncertainty) into a single net Info × Time effect.

#### Value coding index

We estimated the fraction of above-chance attribute-related response variance that could be explained by subjective value. We first fit each neuron using an ‘attribute model’, which had separate weights for each of ten separate attributes that could influence choices, and a ‘value model’, which replaced them with a single attribute, ‘Value’, equal to the estimated subjective value of the offer derived from a model fit to behavior. We quantified the fraction of response variance that each model explained above chance with *R*^2^_cor_, the shuffle-corrected *R*^2^ value. Shuffle correction corrected for extra variance that the attribute model could explain by chance simply due to having more parameters. The value coding index was then defined as

$$\text{Value coding index}=c\left(\frac{{R}_{{{\mathrm{cor }}}}^{2}\text{ for value model}}{{R}_{{{\mathrm{cor }}}}^{2}\text{ for attribute model}}\right)$$

where $c\left(x\right)=\max (0,\min \left(1,x\right))$ clamps the index between 0 and 1 (for rare cases when the index was slightly outside those bounds due to small variations in shuffles used for correction). Importantly, the index is only meaningful if the attribute model explained substantial response variance (because the ratio is only meaningful when its denominator is >0). Hence, we only computed it for neurons with strong attribute effects, defined as significantly attribute-responsive neurons with *R*^2^_cor_ ≥0.1 for the attribute model.

#### Simulated data

Using model fits to the real dataset, we estimated several parameters of each neuron’s coding, including its signal strength and noise level. We used these quantities to generate simulated datasets representing how each neuron would have responded under alternate hypotheses of that neuron’s coding: (1) single attributes (only encodes one randomly selected attribute), (2) random mixtures (same attribute weights as the real data but shuffled and sign flipped), (3) partial integration (a random half of attributes are weighted consistent with ‘random mixtures’, whereas the other half are weighted consistent with subjective value) and (4) full integration (all attribute weights consistent with subjective value).

#### Choice-predictive activity

We computed a choice predictive index as a measure of how variations in LHb neuronal activity above and beyond those accounted for by our neuronal model were predictive of variations in choice behavior above and beyond those accounted for by our behavioral model. We fit each neuron’s neural data with a neural model separately for each offer and fit the behavioral choices on those same trials with an analogous behavioral model. To make the index interpretable, we wanted to ensure that a positive index means that neural signals treating an offer as high value are associated with greater choice of that offer, regardless of the sign the neuron uses to code that value signal (positive or negative). Hence, rather than fitting raw firing rates, we fit ‘normalized value signals’ (*NVS*), defined as the normalized firing rate that has been sign flipped based on that neuron’s fitted sign of value coding (derived from the value model in the value coding index analysis described above). We then computed the behavioral and neural residuals as the difference between the actual data and the prediction derived from the fitted model

$${\Delta }_{{{\mathrm{behavioral}}}}={\mathrm{choice}}-({\mathrm{predicted }}\,p\left({\mathrm{choice}}\right))$$

$${\Delta }_{{{\mathrm{neural}}}}={NVS}-({\mathrm{predicted}}\,{NVS})$$

The choice predictive index was then defined as the Spearman’s rank correlation of the residuals,

$${\mathrm{Choice}}\, {\mathrm{predictive}}\, {\mathrm{index}}={{\mathrm{corr}}}\left({\Delta }_{{{\mathrm{behavioral}}}},{\Delta }_{{{\mathrm{neural}}}}\right)$$

#### RPEs

We computed an RPE index for each neuron. First, we defined the RPE on each correctly performed trial as the difference between the delivered reward and the chosen offer’s E[r]. Next, we selected trials in four categories, defined by the 2 × 2 combinations of chosen offer informativeness (Info or Noinfo) and resulting RPE (positive or negative corresponding to RPE values of >0.1 ml or <−0.1 ml of juice). This produced four conditions, InfoPos, InfoNeg, NoinfoPos and NoinfoNeg. We analyzed single-trial firing rates on these trials after two task events where RPEs commonly occurred in our task: a postcue time window 150–750 ms after cue onset and a postreveal time window 150–750 ms after reveal onset. We used this activity to compute separate measures of the strength of RPE-related responses in each of those time windows (*RPE*_cue_ and *RPE*_rev_), described below. Importantly, *RPE*_cue_ is focused on Info trials, whereas *RPE*_rev_ is focused on Noinfo trials. This is because the cue should only produce RPEs on Info trials because it only provides new information about the outcome on Info trials^10,26^. Similarly, the reveal should only produce RPEs on Noinfo trials because it only provides new information about the outcome on Noinfo trials; on Info trials, the cue already indicated the outcome, so the reveal provides no new information^10,26^. Indeed, our LHb neurons produced prominent RPE signals at those times. Thus, *RPE*_cue_ was designed to reflect the degree to which a neuron’s cue period activity reflects RPEs in Info trials (when RPEs should occur during the cue period) versus Noinfo trials (when they should not occur during the cue period), while *RPE*_rev_ for the reveal period did so in the analogous manner for Noinfo trials versus Info trials, as follows:

$$\begin{array}{l}{{RPE}}_{{{\mathrm{cue}}}}={{\mathrm{ROC}}}\left({{\mathrm{InfoPos}}\; {\mathrm{postcue}}},{{\mathrm{InfoNeg}}\; {\mathrm{postcue}}}\right)\\-{{\mathrm{ROC}}}\left({{\mathrm{NoinfoPos}}\; {\mathrm{postcue}}},{{\mathrm{NoinfoNeg}}\; {\mathrm{postcue}}}\right)\end{array}$$

$$\begin{array}{l}{{RPE}}_{{{\mathrm{rev}}}}={{\mathrm{ROC}}}\left({{\mathrm{NoinfoPos}}\; {\mathrm{postrev}}},{{\mathrm{NoinfoNeg}}\; {\mathrm{postrev}}}\right)\\-{{\mathrm{ROC}}}({{\mathrm{InfoPos}}\; {\mathrm{postrev}}},{{\mathrm{InfoNeg}}\; {\mathrm{postrev}}})\end{array}$$

where ROC(*x*,*y*) is the the ROC area^102^ between the set of single-trial firing rates in condition *x* and those in condition *y*, such that the ROC area is >0.5 if condition *x* generally has higher activity than condition *y*, <0.5 if condition *x* generally has less activity than condition *y* and 0.5 if the distributions are the same. Significance was assessed by permutation tests (*P* < 0.05, *n* = 400 permutations), where each of these measures was compared to the distribution of the same measure computed on permuted datasets in which firing rates were shuffled between InfoPos and NoinfoPos and between InfoNeg and NoinfoNeg, reflecting the null hypothesis that activity was not different between the conditions during which RPEs should and should not occur. Finally, the total RPE index was computed as the mean of these two measures,

$${\mathrm{RPE}}\, {\mathrm{index}}=({{RPE}}_{{{\mathrm{cue}}}}+{{RPE}}_{{{\mathrm{rev}}}})/2$$

and its *P* value was computed from the *P* values of the two constituent measures using a Fisher’s combination test^98^^,103^.

#### Overlap between coding properties

For each neural population, we tested the overlap between the three coding properties measured by the indexes described above: value coding index, choice predictive index and RPE index. We classified cells as strongly value coding if they had a value coding index > 0.6 and as significantly choice predictive or RPE coding if their respective indexes were significant (*P* < 0.05). This analysis included all attribute-responsive neurons for which all three coding properties could be computed. We classified neurons as ‘combined coding’ if they passed all three criteria and all three had identical coding signs. For example, a combined coding neuron that has all three coding properties with negative signs would have offer responses negatively related to offer subjective value, a lower firing rate during offer responses predicting greater choice of that offer and a lower firing rate during cue/reveal responses associated with more positive RPEs. We used permutation tests (*n* = 200,000 permutations in which each index was independently shuffled across neurons) to test whether the proportion of ‘combined coding’ neurons in an area was greater than chance and whether the difference of that proportion between areas was significantly greater than chance. Finally, we plotted a Venn diagram depicting these neurons, where the area of each of the three main circles was proportional to the percentage of these neurons with the corresponding coding property, and the intersections between pairs or triples of circles represented neurons that had pairs or triples of coding properties and coded them with identical signs. The diagram was optimized by fixing the areas of each circle and adjusting the centers of the circles to minimize the total error between the desired and displayed areas of the regions representing the intersections between sets of circles.

#### Analysis of electrical stimulation

As a first pass, we used psychometrics to test whether LHb electrical stimulation after the onset of an offer influenced the subsequent choice of that offer. We calculated psychometric curves representing the percent choice of offer 2 as a function of the estimated difference in subjective value between offer 2 and offer 1. To do this, for each animal, we first fit the main model of the second version of the monkey task to the subset of choice data collected from stimulation sessions (Supplementary Table 1) using only trials in which there was no stimulation during the offers. Based on this model fit, we used the procedures described above to derive an estimate of the subjective value of offer 1 and offer 2 (V1 and V2) and the difference between the two offer values (V2 – V1) on each trial. All of these estimated values were in units of log odds of influencing choice. Importantly, while the model was only fit on trials without offer period stimulation, we then derived its value estimates even for trials in which stimulation was applied. Thus, (V2 – V1) gave us an estimate of what the difference in estimated subjective value of the two offers ‘should have been’ on those trials if LHb stimulation had not been applied. We then plotted the percent choice of offer 2 as a function of (V2 – V1) separately for trials with no stimulation, trials with offer 1 stimulation and trials with offer 2 stimulation. For each curve, we estimated the indifference point using the same approach described above (that is, by fitting the underlying choice data with a logistic function and using the confint function in MATLAB to compute its confidence interval). Finally, we tested whether the 95% confidence interval of the indifference point excluded 0.

Next, we quantified the stimulation effect more precisely by using a modeling approach. First, to measure the mean effect of stimulation regardless of whether it occurred during offer 1 or offer 2, we initially fit each animal’s total behavioral dataset during stimulation sessions with a model with three attributes: (V2 – V1), Stim1 and Stim2 (Supplementary Table 3). To measure the LHb stimulation effect from each individual session, we fit this model to each individual session separately.

### Reporting summary

Further information on research design is available in the Nature Portfolio Reporting Summary linked to this article.

## Online content

Any methods, additional references, Nature Portfolio reporting summaries, source data, extended data, supplementary information, acknowledgements, peer review information; details of author contributions and competing interests; and statements of data and code availability are available at 10.1038/s41593-023-01511-4.

### Supplementary information

Supplementary Information Supplementary Figs. 1–19, Tables 1–6 and Modeling Note.

Reporting Summary

## Data Availability

Data from this study are available upon reasonable request to the corresponding author and will be made publicly available 1 year after publication at https://github.com/ethan-bromberg-martin/a-neural-mechanism-for-conserved-value-computations-integrating-information-and-rewards.

## Code availability

Code from this study is available upon reasonable request to the corresponding author and will be made publicly available 1 year after publication at https://github.com/ethan-bromberg-martin/a-neural-mechanism-for-conserved-value-computations-integrating-information-and-rewards.
