# Supplementary material for: A neural mechanism for conserved value computations integrating information and rewards
Source: Nat Neurosci. 2024 Jan 4;27(1):159–75. doi: 10.1038/s41593-023-01511-4 (PMC10774124; doi:10.1038/s41593-023-01511-4)
Supplement: Supplementary file 1 — Supplementary Figs. 1–19, Tables 1–6 and Modeling Note. [file 41593_2023_1511_MOESM1_ESM.pdf]

nature neuroscience

Article

https://doi.org/10.1038/s41593-023-01511-4

# A neural mechanism for conserved value computations integrating information and rewards

In the format provided by the authors and unedited

# Supplemental Material

The supplement includes Figures S1-S19, Tables S1-S6, and a Modeling Note.

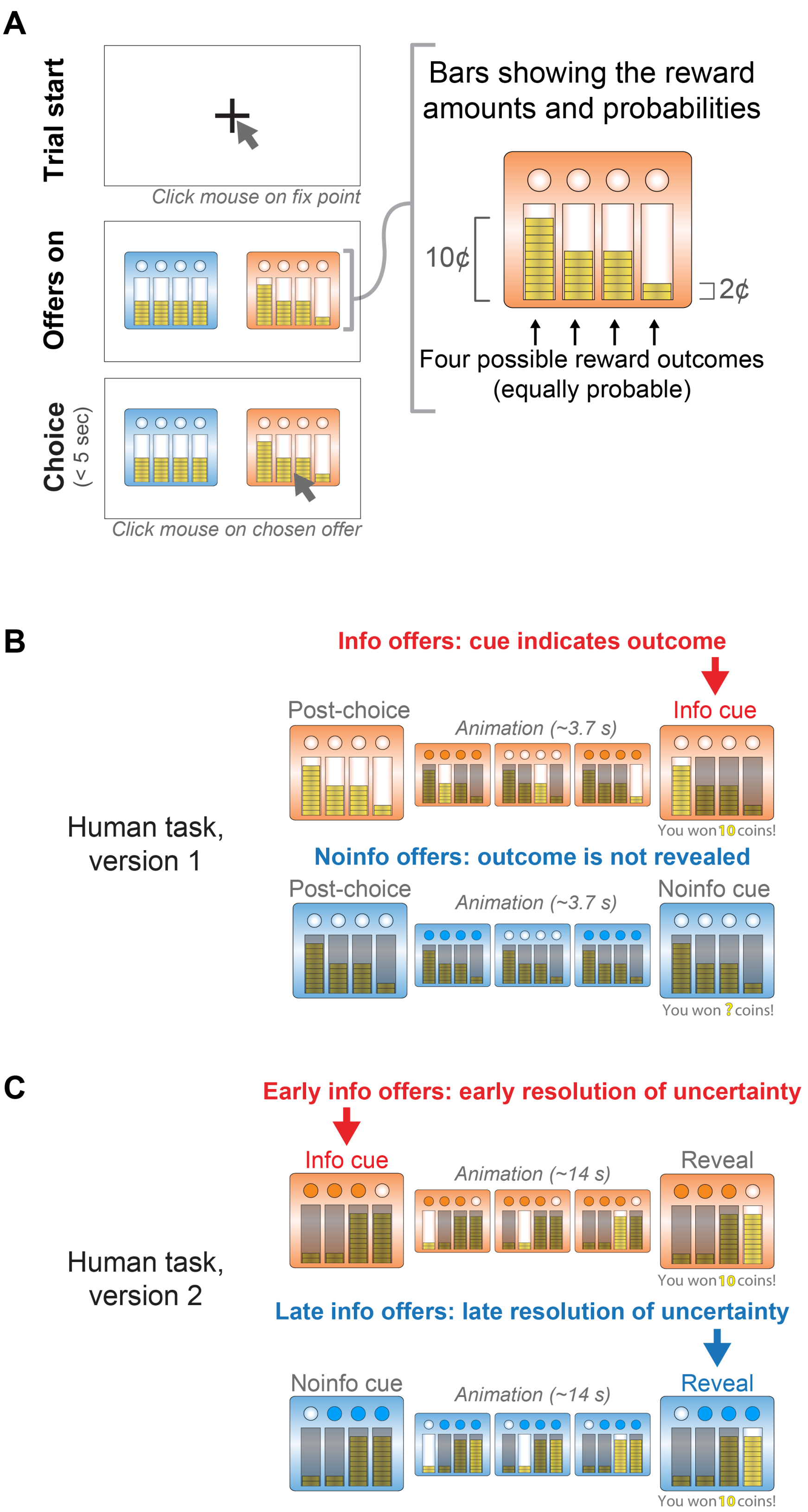


**Figure S1. The multi-attribute information choice tasks for humans.** (A) After clicking on a fixation point, participants were presented with two offers and chose between them by clicking on the chosen offer. Same as Figure 1A. (B) In the first version of the task, after choosing an Info offer (top), an ~3.7 s long animation would play in which a single coin stack at a time would be 'lit up' for a period of 0.19 s, with the 'lit' location initially being a random stack and then cycling sequentially among the stacks from left to right (wrapping around from

the rightmost stack to the leftmost stack). The animation ended after 4 full cycles had been completed and the currently lit bar was the one the computer selected to be delivered, at which point text appeared indicating the number of coins that had been won (e.g. “You won 11 coins!”). In addition, during the animation the small circular lights at the top of the offer all alternated between ‘lit’ or ‘not lit’ each time the lit stack changed. After choosing a Noinfo offer (bottom), a matched ~3.7 s long animation would play, in which the small circular lights at the top of the offer all alternated between ‘lit’ and ‘dark’ just as they did for Info offers, but in which no coin stacks were lit, and the text at the end did not reveal the number of coins that had been won (e.g. “You won ? coins!”). This non-informative text was provided so that both Info vs Noinfo trials would have a relatively close match in the visual and timing-related features of their feedback displays; it is possible that this text also contributed to information preferences, by emphasizing the unknown nature of the outcome on Noinfo trials. (C) In the second version of the task, after choosing an Early Info offer (top), an animation was played which was very similar to the Info animation in the first version of the task, except that it ended after 8 cycles with each animation frame lasting 0.38 s (for a total duration of ~14 s from choice to final reveal), and immediately after the choice and 1 s before the cycling animation began, one of the four small circular lights at the top of the offer became ‘lit’ and the other three lights became ‘dark’, which accurately indicated the stack that the computer had selected to be delivered on that trial (“Info cue”). Thus if participants chose Early Info, they got early information to resolve their uncertainty about which of the four possible outcomes they would receive. After choosing a Late Info offer (bottom), the same animation occurred except that the ‘lit’ circular light at the top of the offer was randomized and hence did not provide any information about the outcome (“Noinfo cue”). Thus participants only got information to resolve their uncertainty at a later time, from the final ‘lit’ coin stack and the text message indicating their winnings.

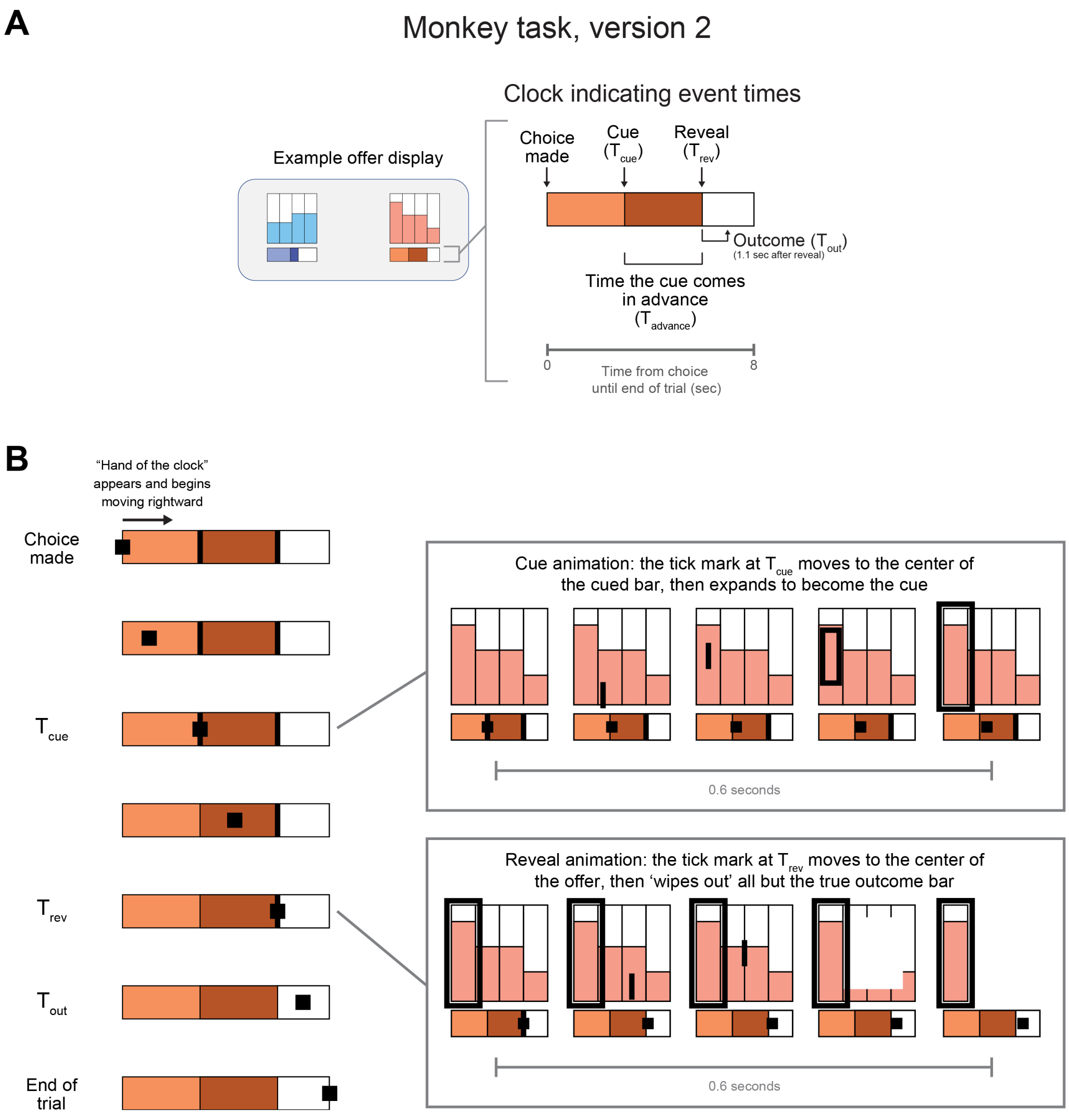


**Figure S2. The second version of the multi-attribute information choice task for monkeys, manipulating cue and reward timing.** (A) Example Info and Noinfo offer stimuli. Note that the stimuli shown on the screen were complex visual textures sourced from scenes (Methods), which here are represented as shades of red and blue. The top part of the offer indicates the reward distribution (same as the first version of the task). The bottom part is a horizontal bar indicating the timing of task events if the offer is chosen. The leftmost edge of the bar indicates the time of the choice, and the rightmost edge indicates the end of the trial (which was always 8 seconds after the choice). Vertical lines, and changes in the bar texture, indicate Tcue and Treveal. The reward was always delivered 1.1 s after Treveal. (B) After the choice, an animated "hand of the clock" appeared on the left edge of the bar. The location of the hand indicated the current time during the trial. The hand moved rightward at a constant rate, eventually touching the right edge of the bar simultaneously with stimulus offset and the end of the trial. When the hand touched the first vertical line at Tcue, the line moved upward and morphed to become the cue. When the hand touched the second vertical line at Treveal, the line moved upward and revealed the outcome by 'wiping out' three of the possible reward bars, so that only the single reward bar that controlled the trial's outcome remained on the screen.

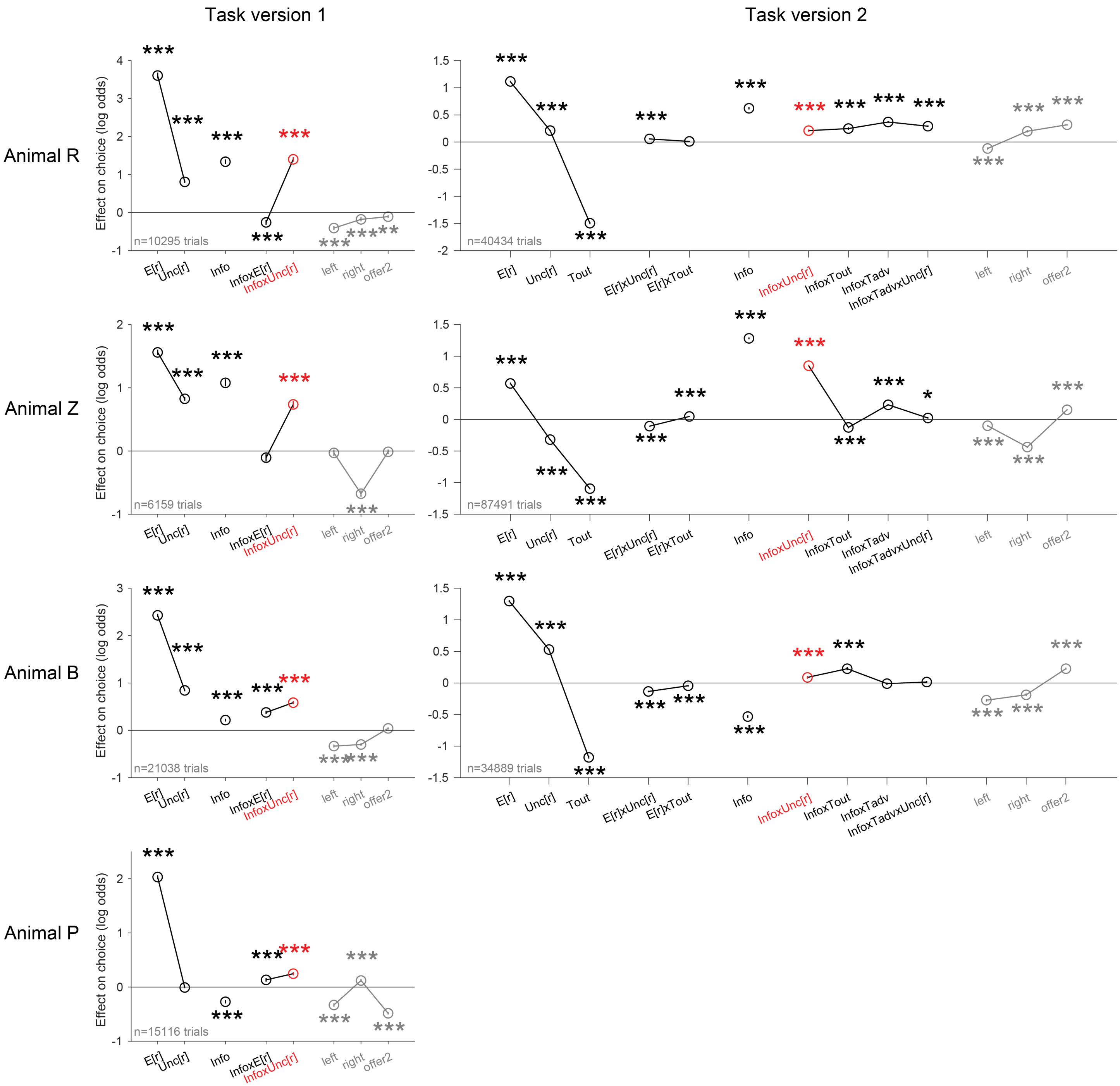


**Figure S3. Complete behavioral model fits for each animal and task version.** Rows indicate animals. Columns indicate task versions with data from that animal (version 1: fixed event timing; version 2: variable timing with visible clock). Black and red data points are terms reflecting the influence of motivational attributes on choice. Gray data points on the right side of each plot are bias terms representing the effect of offer location on the screen (left or right vs center), and presentation order (offer 2 vs offer 1). Data are fitted parameters ± SE.

All animals had significant positive effects of E[r] in all tasks (juice seeking), and all animals tested with task version 2 had significant negative effects of $T_{out}$ (temporal discounting). Attitudes toward risk were generally positive but could vary across animals or tasks (non-significant in Animal P in task version 1, and significantly negative in Animal Z in task version 2). All animals had a significant main effect of Info ($p < 0.001$) and a significant Info x Unc[r] interaction ($p < 0.001$) in each task version. In task version 2 where event timing was manipulated, all animals had at least one highly significant interaction between Info and a time-related

variable ($p < 0.001$). Animals R and Z, from which the neurons were recorded, had significant effects of all three interactions (Info x Tout, Info x Tadvance, Info x Tadvance x Unc[r]), notably with significant positive Info x Tadvance (i.e. placing greater value on Info when it arrived early in advance of the outcome) and Info x Tadvance x Unc[r] (i.e. doing so even more so when the outcome had high uncertainty).

Importantly, Info x Unc[r] effects were generally positive even in occasional individuals with negative main effects of Info. This suggested that even on occasions when individuals had a net aversion to information, they still placed higher value on that information when it resolved a higher amount of uncertainty. This could be seen in two monkeys: Animal P in task version 1 and Animal B in task version 2. In both cases they had a significant negative main effect of Info and positive effect of Info x Unc[r] (both $p < 0.001$).

Humans showed a similar tendency. In human task version 1, n=64 humans had a negative main effect of Info. Their median Info x Unc[r] effect was significantly positive (mean = $0.160 \pm 0.046$, $p = 0.008$, signed-rank test). Also, these individuals were significantly more likely to have significantly positive Info x Unc[r] effects than expected by chance (9/64 significantly positive, $p < 0.001$; 1/64 significantly negative, $p = 0.80$; one-tailed binomial tests). A similar trend occurred for the n=24 of these humans whose negative main effect of Info reached significance. Their median Info x Unc[r] effect trended positive (mean = $0.163 \pm 0.068$, $p = 0.068$, signed-rank test) and they were significantly more likely to have significantly positive Info x Unc[r] effects than expected by chance (3/24 significantly positive, $p = 0.021$; 0/24 significantly negative, $p = 1.000$; one-tailed binomial tests).

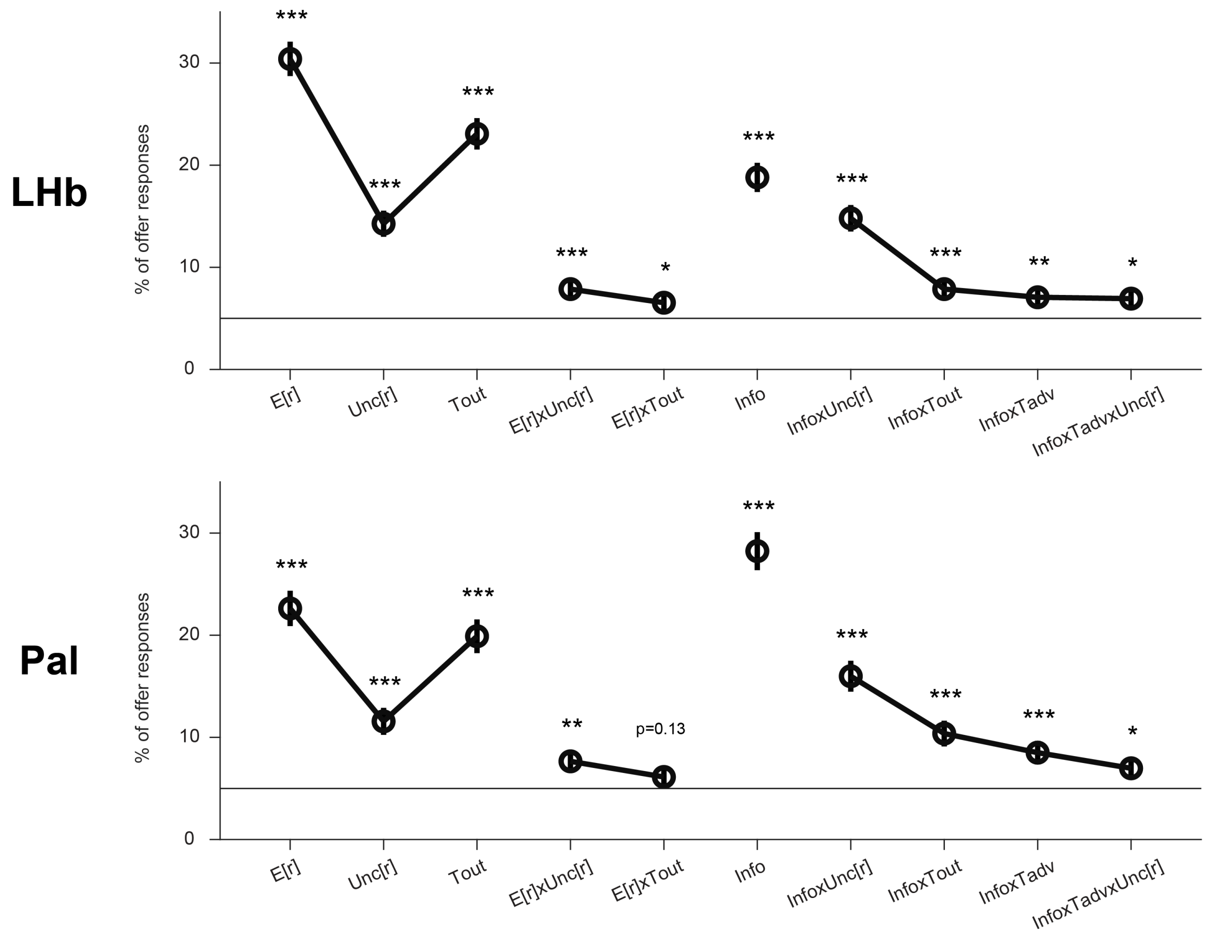


**Figure S4. LHb and Pal offer responses with significant effects of each offer attribute.** Shown are the percentage of offer responses in each area with significant effects of each offer attribute ($p < 0.05$, from the GLM fit using the standard model of the task used for neuronal recording (Model 8). Top: LHb (n= 375 total neurons x 2 offers). *Bottom:* Pal (n=294 total x 2 offers). Data are fitted parameters ± SE. Asterisks indicate that the percentage of significant neurons is greater than expected by chance (one-tailed binomial test, *, **, *** for $p < 0.05, 0.01, 0.001$).

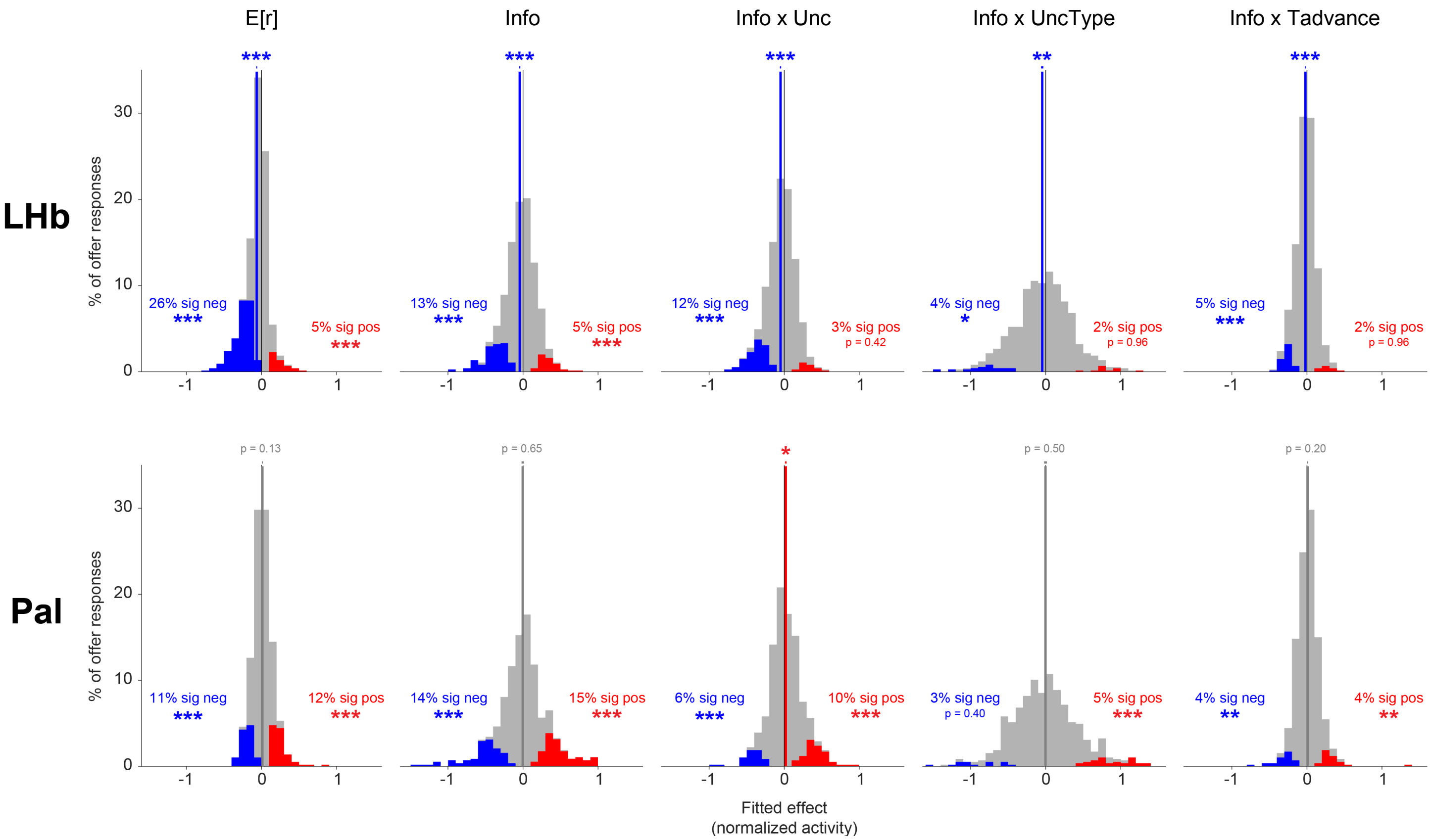


**Figure S5. LHb offer responses had predominantly negative signs of attribute coding while Pal offer responses had mixed negative and positive signs.** LHb offer responses as a population had significantly negative mean effects of each of these offer attributes (blue vertical lines and asterisks at top), and the significant offer responses predominantly had negative signs (blue text on left vs red text on right; lower activity for the setting of an attribute that had higher value). By contrast, Pal offer responses as a population had little or no significant mean effect of each of these offer attributes (predominantly gray vertical lines) and individual significant offer responses were a roughly even mix of negative and positive signs (blue text on left vs red text on right). Rows show data from LHb (top) and Pal (bottom). Columns show different motivational attributes. Histograms show the percentage of neurons (y-axis) with fitted effects of expected juice and key information-related attributes on normalized neuronal activity (x-axis) for each neuron's response to each offer. One offer response was excluded for one Pal neuron due to the model fit not converging, thus yielding a total of n=750 offer responses for LHb and n=587 offer responses for Pal. All attribute effects except Info x UncType are estimated from fits with Model 8 (Methods), while Info x UncType is estimated as the difference between the Info x 25/50/25 and Info x 50/50 weights from Model 12. Blue indicates significantly negative offer responses, red indicates significantly positive offer responses ($p < 0.05$). Vertical line indicates the mean and text or asterisks indicate the p-value or significance of whether the median is different from 0 ($p < 0.05$, signed-rank test). Text on left and right indicate the percentage of offer responses that are significantly negative or positive, and the p-value or significance of whether that percentage is higher than the 2.5% expected by chance under the null hypothesis that neurons do not have true activity related to that attribute (one-tailed binomial test).

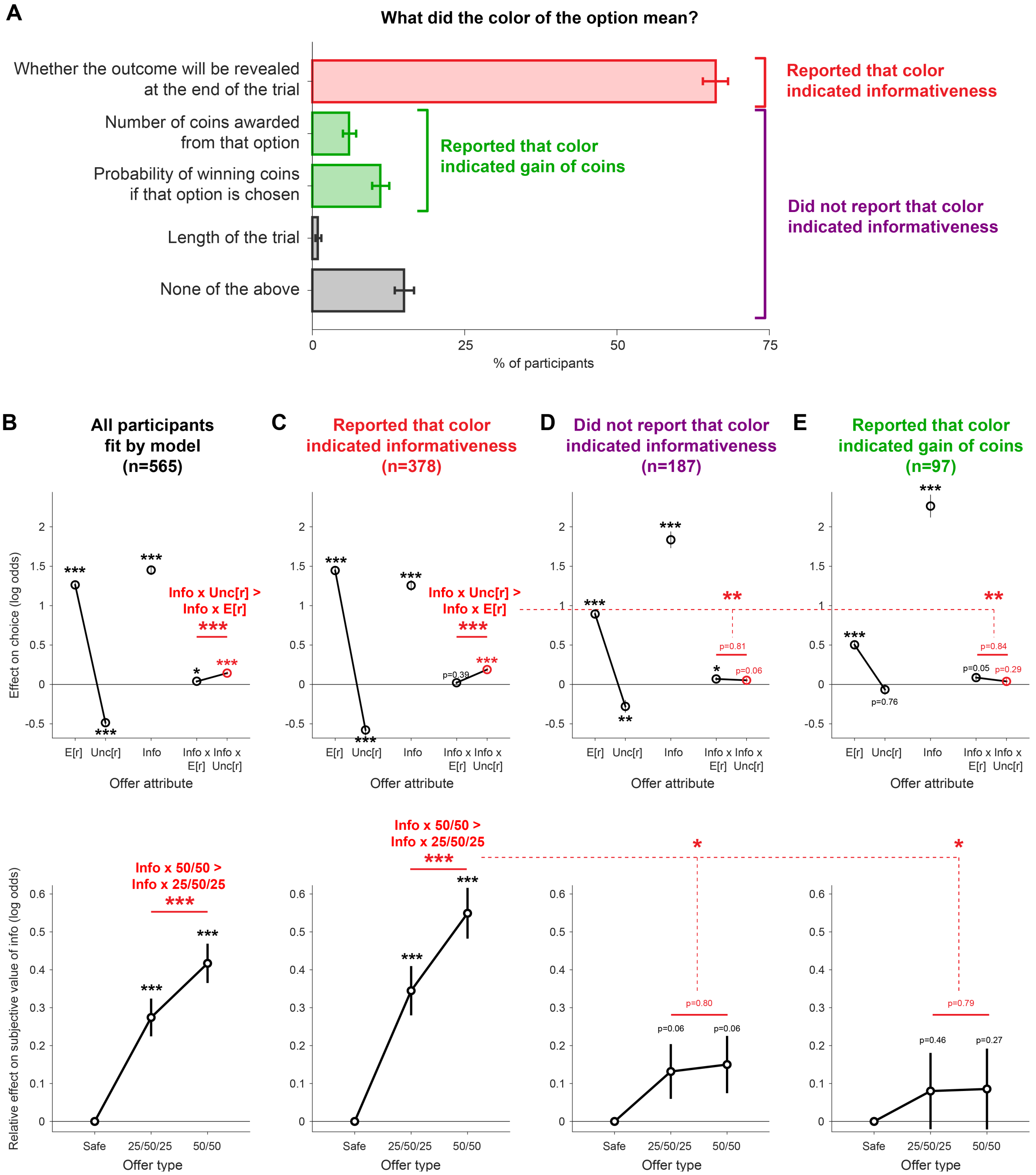


**Figure S6. Strong valuation of information in participants who explicitly reported that offer color indicated informativeness. (A)** Histogram of participant responses to a questionnaire question about the meaning of the color of the options (the exact phrasing of the questionnaire varied slightly across participants; Methods) from the first version of the human task (n=565 participants). Data are mean ± SE. The great majority of participants reported that offer color indicated informativeness (red), but a minority did not report that offer color indicated informativeness (purple), including a subset who specifically reported that offer color indicated the gain of coins (green, either amount or probability of gaining coins). **(B)** Pooled behavior of all participants fit by the models, showing key results. *Top:* mean fitted effects of offer attributes on choice (from Model 1). *Bottom:* mean fitted effects of offer type on the subjective value of information (from Model 5). Data are fitted parameters ± SE. This shows key results including information value scaling up more positively with uncertainty than expected reward (top, red, Info x Unc[r] > Info x E[r], $p < 0.001$, signed-rank test), and the responsible form of uncertainty resembling SD more than Range or Entropy (bottom, red, Info x 50/50 > Info x

25/50/25, $p < 0.001$, signed-rank test). **(C)** Same as (B), for the subset of participants who reported that offer color indicated informativeness. The key results still hold, trending stronger than in the population as a whole, and significantly stronger than in the other subsets of the population shown in (D,E) (dashed lines indicate comparisons, all $p < 0.05$, rank-sum tests). **(D)** Same, for the subset of participants who did not report that offer color indicated informativeness. The key results no longer hold, without any apparent trend or significance. (E) Same, for the subset of participants who reported that offer color indicated gain of coins. The key results no longer hold, without any apparent trend or significance.

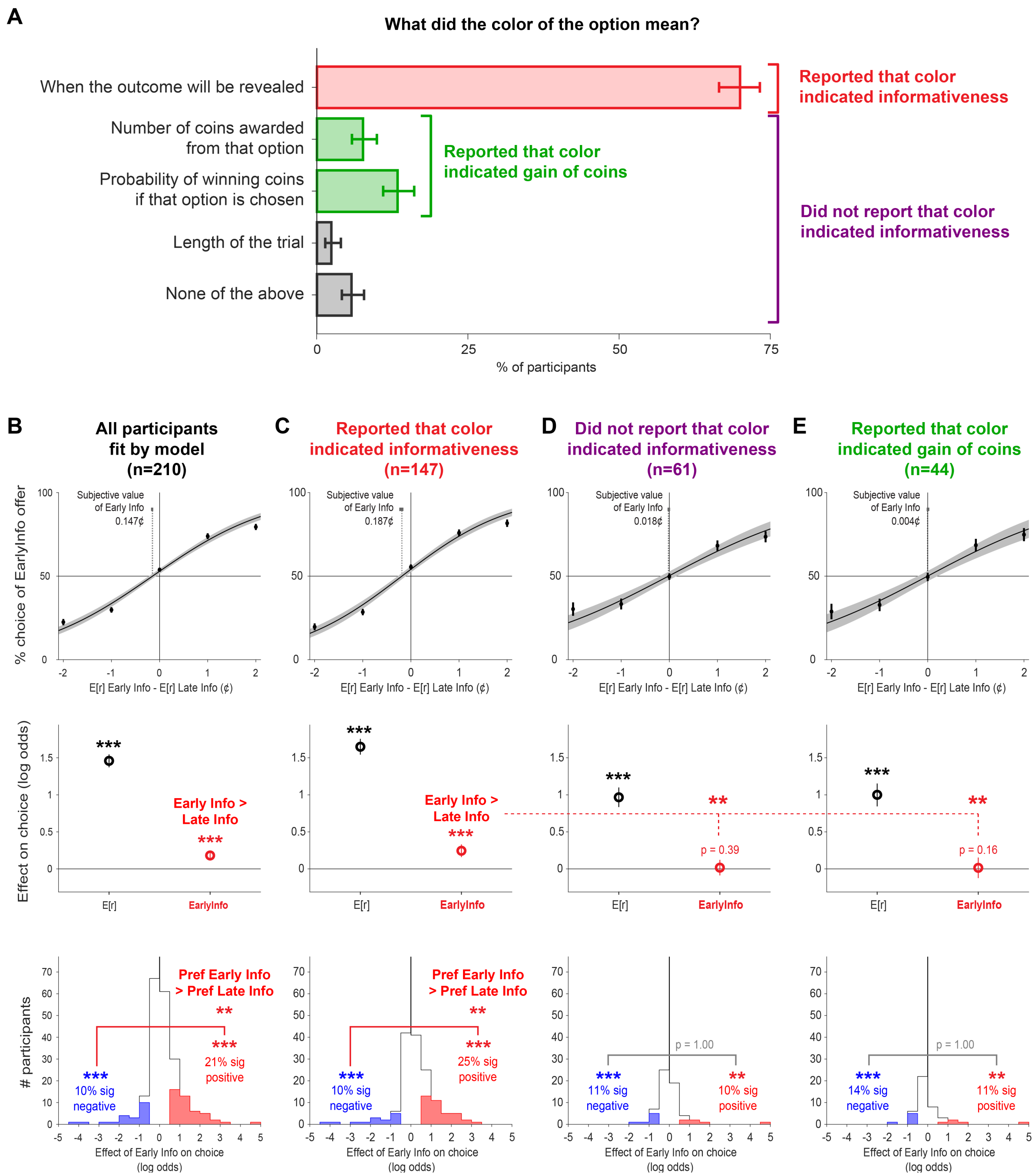


**Figure S7. Strong valuation of information timing in participants who explicitly reported that offer color indicated informativeness. (A)** Same as Figure S6A but for the second version of the human task (n=210 participants). Data are mean ± SE. This task manipulated information timing, so the questionnaire asked whether offer color indicated the time when the outcome will be revealed. Again, the great majority of participants reported that offer color indicated informativeness, but a minority reported otherwise. **(B)** Pooled behavior of all participants fit by the model (Model 7), showing key results. *Top:* psychometric function measuring the subjective value of EarlyInfo. Data are mean ± SE. Shaded area is fitted logistic function ± SE. *Middle:* mean fitted effects of the two motivational attributes manipulated in this task, E[r] and EarlyInfo. Data are fitted parameters ± SE. *Bottom:* distribution of per-participant fitted effects of EarlyInfo. These show the key results that participants put significantly positive subjective value on EarlyInfo (top, middle, $p < 0.001$, signed-rank test) and that significantly more participants put significantly positive value on EarlyInfo than significantly negative value (bottom, red > blue, $p < 0.001$, binomial test). **(C)** Same as (B), for the subset of participants who reported that offer color indicated informativeness. The key results still hold, trending to be

stronger than in the population as a whole, and significantly stronger than the other subsets of the population shown in (D,E) (dashed lines indicating comparisons, all $p < 0.01$, rank-sum tests). **(D)** Same, for the subset of participants who did not report that offer color indicated informativeness. The key results no longer hold, without any apparent trend or significance. (E) Same, for the subset of participants who reported that offer color indicated gain of coins. The key results no longer hold, without any apparent trend or significance.

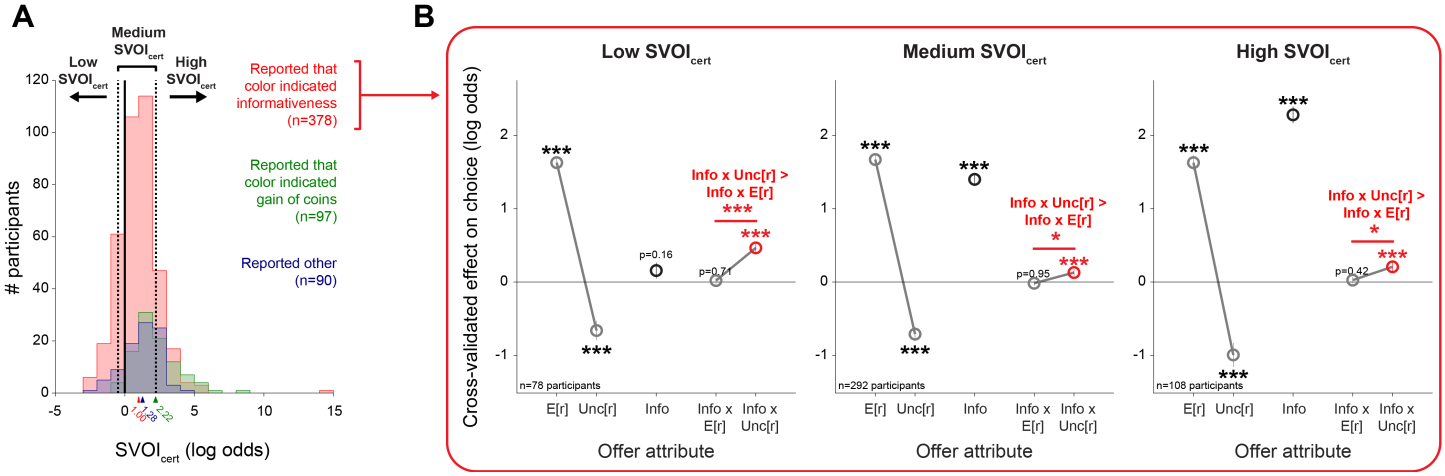


**Figure S8. Information value increased with reward uncertainty regardless of willingness to pay for information about certain outcomes.**

**(A)** For each participant we estimated the subjective value of information about certain outcomes ($SVOI_{cert}$, in units of log odds of choice), based on the fitted parameters of the behavioral model (Model 1). Specifically, $SVOI_{cert}$ = V(Info offer with certain reward) – V(noinfo offer with the same certain reward), expressed in units of log odds of choice. On average, participants had positive $SVOI_{cert}$. Histograms show $SVOI_{cert}$ separately based on participant answers to the questionnaire: those who reported that offer color indicated informativeness (red), gain of coins (green) or other (blue). Arrows under plot indicate mean of each histogram. As one might expect, the minority of participants who reported that offer colors indicated gain of coins had a much higher mean $SVOI_{cert}$ than other groups (green, $SVOI_{cert}$ = 2.22), presumably because they believed that choosing Info offers would actually provide higher monetary reward. However, such a belief cannot fully explain $SVOI_{cert}$, because $SVOI_{cert}$ was still typically positive even for the great majority of participants who reported that offer color indicated informativeness (red, mean $SVOI_{cert}$ = 1.00).

**(B)** To test whether $SVOI_{cert}$ was necessary for our key finding that information value increased with reward uncertainty, we plotted the mean fitted attribute effects from the behavioral model, considering only participants who reported that offer color indicated informativeness (red histogram in A), split separately into subsets where $SVOI_{cert}$ was low (left, lowest 1/7 of participants, negative $SVOI_{cert}$), medium (middle), or high (right, highest 1/7 of participants, very positive $SVOI_{cert}$; boundaries between groups are indicated by dashed vertical lines in A). Data are fitted parameters ± SE. As expected, the main effect of Info varied strongly across groups: it was non-significant in participants with low $SVOI_{cert}$, positive in participants with medium $SVOI_{cert}$, and very positive in participants with high $SVOI_{cert}$. However, all other attributes had similar contributions to value in all three groups. There was a tendency for Info x Unc[r] to be highest in participants with low $SVOI_{cert}$. Crucially, though, the mean Info x Unc[r] effect was significantly positive in all three groups (all $p < 0.001$, signed-rank tests), and was significantly higher than the mean Info x E[r] effect in all three groups (all $p < 0.05$, signed-rank tests). Thus, information value increased with reward uncertainty, regardless of whether participants had low, medium, or high willingness to pay for information about certain outcomes.

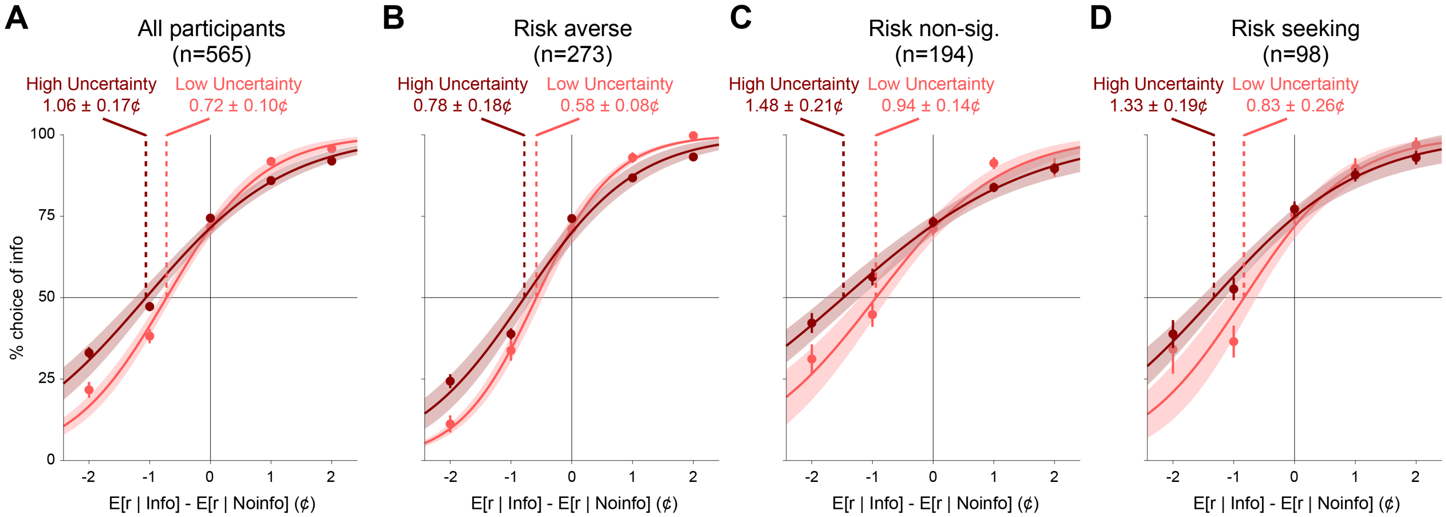


**Figure S9. Distinct attitudes toward risk versus information to resolve uncertainty. (A)** Psychometric function using pooled data from human participants in the first version of the human task (same as in the main text). Data are mean ± SE. Shaded areas are fitted logistic function ± SE. **(B,C,D)** Same, but only using participants who were categorized as significantly risk averse (B, significant negative effect of Unc[r] on choice, $p < 0.05$ from GLM fit), risk non-significant (C, $p > 0.05$), or risk seeking (D, significant positive effect). The bootstrap SE of the subjective value of information was computed using all bootstrap resamples where $|\beta| > 0.01$, where $\beta$ is the slope parameter of the fitted psychometric curve. Despite their very distinct attitudes toward risk *per se*, these three groups all tended to respond to reward uncertainty by increasing the subjective value of obtaining information (compare dashed lines indicating subjective value as measured by willingness to pay for information, “High Uncertainty” vs “Low Uncertainty”).

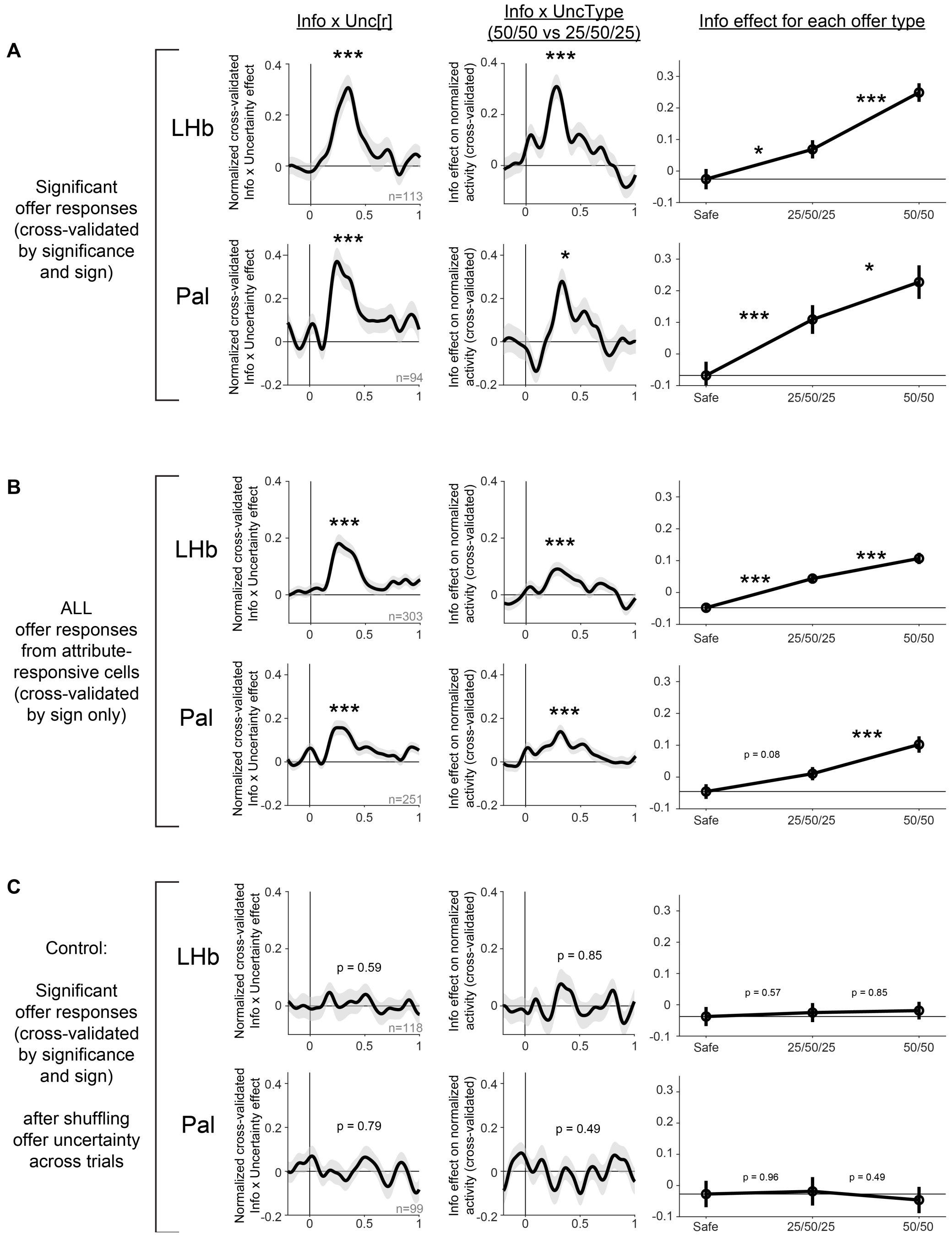


**Figure S10. Validation of the cross-validation method for measuring Info x Uncertainty and Info x UncertaintyType effects on neuronal activity.** (A) Results shown in the main text figures, using a method with the goal of plotting significant Info x Uncertainty effects, while using cross-validation to correct for selection bias. In this procedure, each neuron's response to each offer was only selected for inclusion in the analysis based on the neuron's response to the *other* offer. This procedure had two steps. First, each neuron's responses to Offer 1 were only included if that neuron's response to Offer 2 had a significant Info x Unc[r] effect (according to any of the three models representing hypothetical forms of uncertainty – SD, Range, or Entropy (Models 8-10)). Second, to ensure that more positive activity corresponded to a stronger Info x Uncertainty signal, each neuron's responses to Offer 1 were sign-flipped based on the sign of that neuron's Info x Uncertainty effect for Offer 2. The same procedure was used to decide whether to include and/or sign-flip each neuron's responses to Offer 2 (i.e. based on its responses to Offer 1). Data are mean ± SE. Shaded areas are mean ± SE. (B) The analysis produces very similar results even without step 1. That is, this version

includes *all* attribute-responsive neurons in each area (instead of using step 1 to select significant Info x Unc[r] responses), but still uses cross-validation to align the sign of neuronal responses (step 2). This produces similar results, though smaller in magnitude. This is what one would expect, because this analysis includes *all* attribute-responsive neurons, without attempting to select for ones with true Info x Uncertainty-related signals. (C) These Info x Uncertainty effects are not spurious effects of the cross-validation procedure, because they disappear when the analysis is run on a control, shuffled dataset that lacks true Info x Uncertainty effects. To generate this control dataset, we kept all neuronal responses intact but shuffled each neuron's uncertainty-related attributes across trials. This shuffling destroyed all uncertainty-related coding effects in the dataset while keeping all other attribute effects on activity intact. As desired, the analysis no longer shows any apparent Info x Uncertainty effects (column 1) or Info x UncertaintyType effects (columns 2,3).

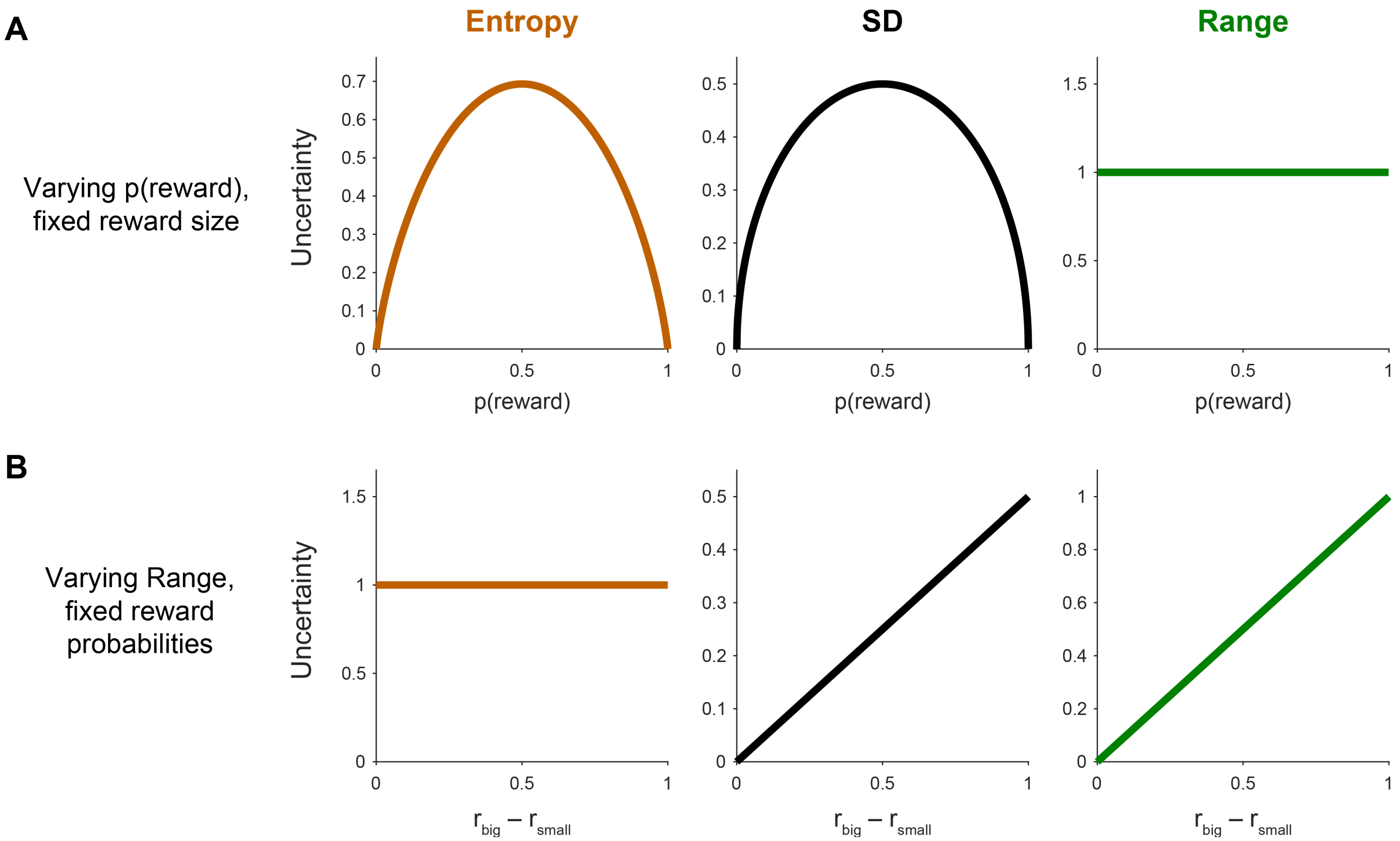


**Figure S11. Necessity to manipulate multiple parameters of reward distributions to dissociate hypothesized forms of uncertainty.** (A) One type of standard task used to study uncertainty has conditions with different reward probabilities while maintaining a fixed reward magnitude (e.g.[58,104,105]). This produces a classic inverted-U shaped function relating reward probability to certain measures of uncertainty (e.g. Entropy and SD; left and middle) but not other measures (e.g. Range; right), as shown in this plot in which the reward size is fixed at 1 and the reward probability is varied from 0 to 1. Thus, this type of task can measure behavior and neural activity related to certain forms of uncertainty, but cannot clearly distinguish between them, and cannot measure behavior and activity related to other forms of uncertainty (as has been pointed out by authors using those tasks[105,106]). (B) A second type of standard task used to study uncertainty has conditions with different ranges between large vs small reward magnitudes while maintaining fixed reward probabilities (e.g.[12,49,107]). This produces a linear function relating reward range to certain measures of uncertainty (e.g. SD and Range; middle and right) but not other measures (e.g. Entropy; left), as shown in this plot in which the reward probabilities are fixed at p(big reward) = p(small reward) = 0.5 and the difference between the reward sizes is varied from 0 to 1. Thus, again, this type of task can measure behavior and activity related to certain forms of uncertainty, but cannot clearly distinguish between them, and cannot measure behavior and activity related to other forms of uncertainty (as has again been pointed out by authors using these tasks[108]).

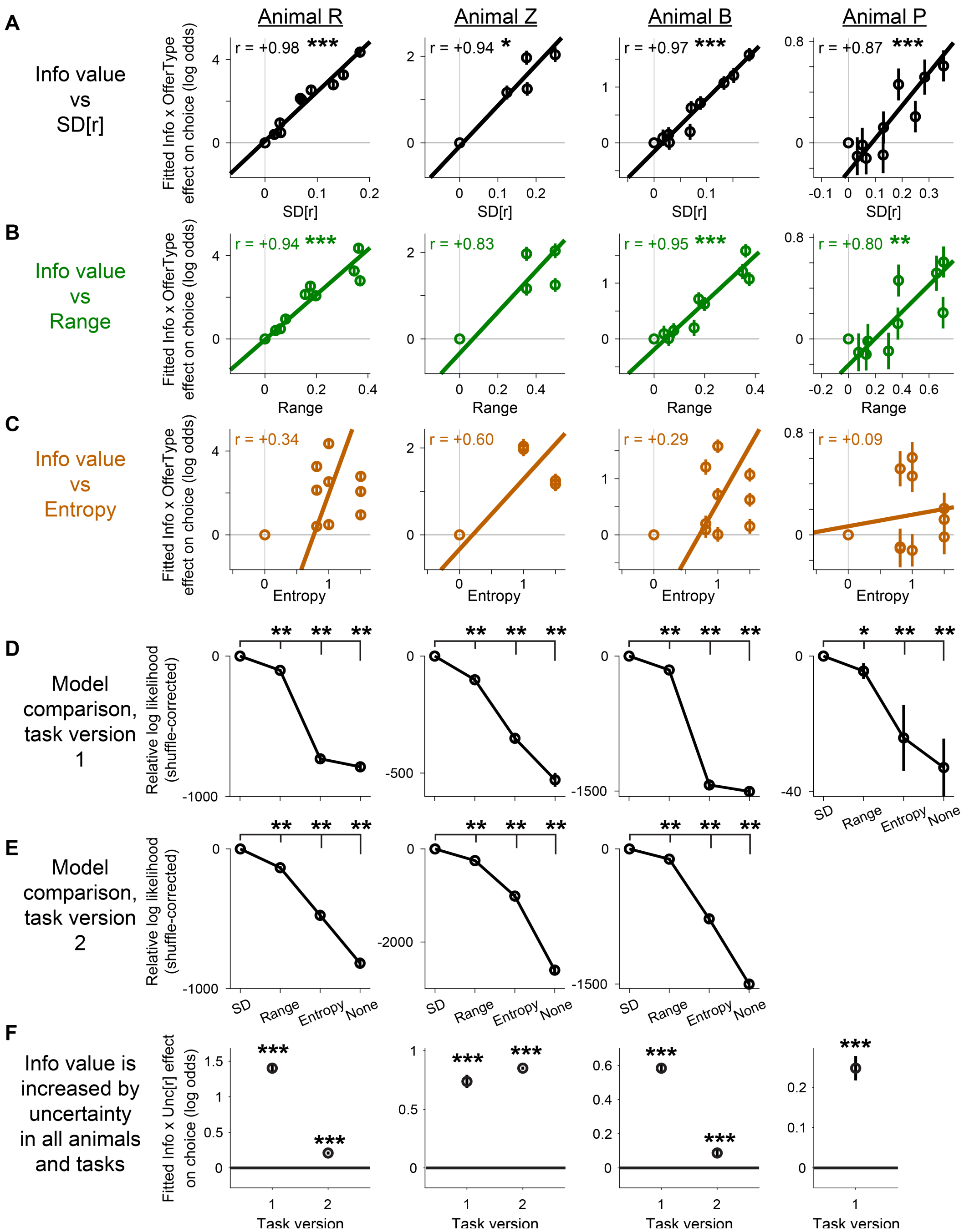


**Figure S12. Consistent Info x Uncertainty Type effects across monkeys, tasks, and reward distributions.** (A-C) Correlation between each candidate uncertainty measure – SD, Range, and Entropy, shown in panels A, B, and C – with the fitted Info x Uncertainty Type weights from a more detailed version of the model used in the main text, that included a more detailed classification of offers into uncertainty types (Table S2). In brief, this included all combinations of up to three types of reward distributions (25/50/25, 50/50, 25/75) x up to two types of risk levels (low range, high range), depending on which distributions were tested in each animal (Table S1). The weights are highly correlated with SD, less correlated with Range, and least correlated with Entropy. Data are mean ± SE. Text indicates Pearson's linear correlation and asterisks indicate significance (*, **, *** indicate $p < 0.05$, 0.01, 0.001). The colored line is a linear fit from type 2 regression. N=10295, 6159, 21038, and 15116 trials for animals R, Z, B, P. (D) Direct comparison between models of behavior in which uncertainty is defined as SD, Range, or Entropy, or in which no uncertainty term is included (Table S2) separately for each animal, in the first version of the monkey task. Same format and data as Fig. 2H. The SD model fit best in all animals. Data are fitted relative log likelihood ± bootstrap SE. (E) Same, in the second

version of the monkey task, for all animals studied with the task. The SD model fit best in all animals. N=40434, 87491, 34889 trials for animals R, Z, B. (F) Fitted weight of Info x Unc[r], measuring how the subjective value of information grows with uncertainty, from the main behavioral model for each animal and task (Table S1, Model 1 ("Distrib SD") for task version 1 and Model 8 ("Clock SD") for task version 2). All animals increased the value of information with uncertainty, as indicated by significantly positive weights. Data are fitted parameters ± SE. *, **, *** indicate $p < 0.05, 0.01, 0.001$.

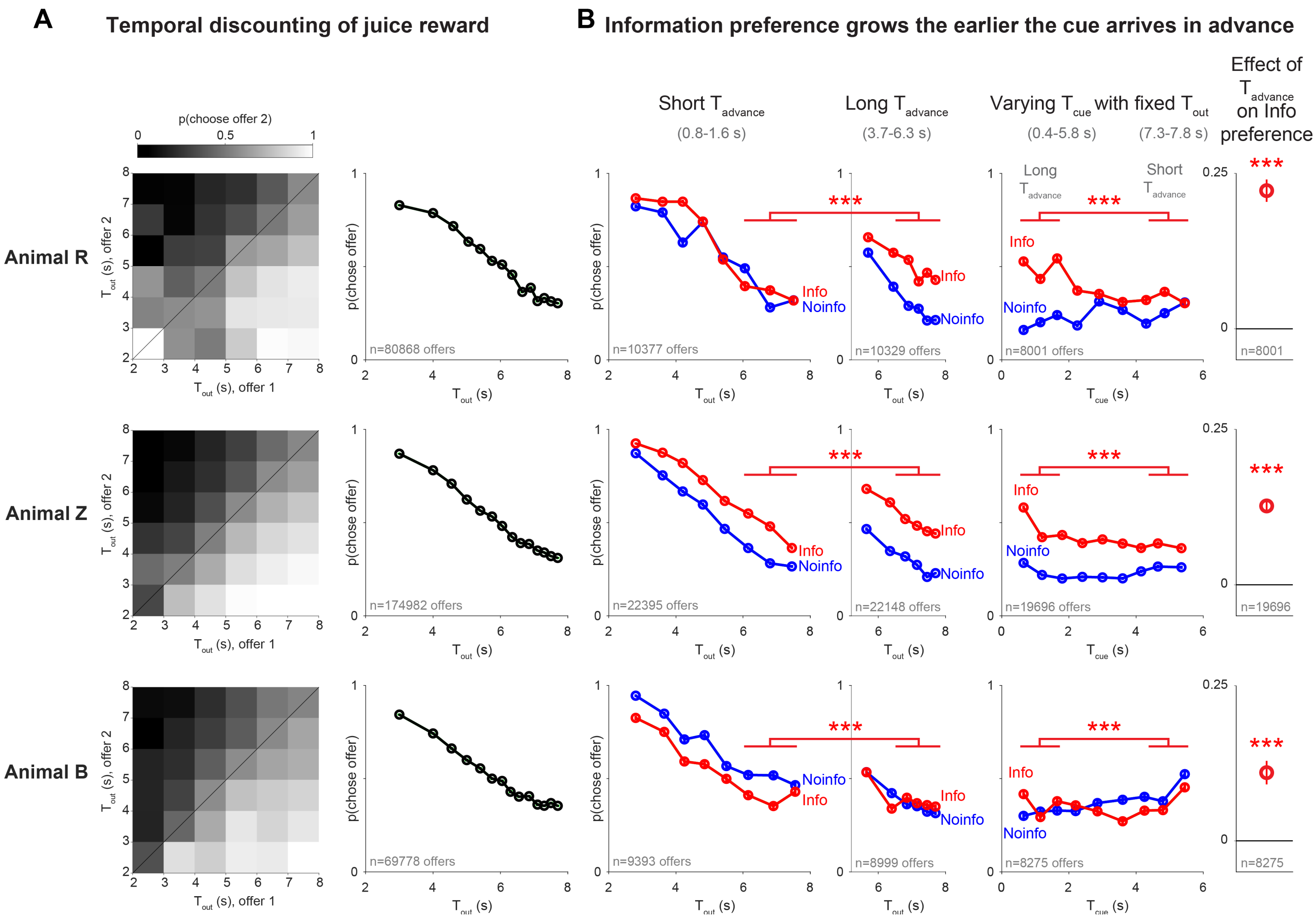


**Figure S13. Monkeys use time to value both juice and information.** Data is shown separately for each animal (rows). (A) Temporal discounting of juice reward. *Left:* how the outcome delivery time Tout for offer 1 (x-axis) and offer 2 (y-axis) affects the probability of choosing offer 2 (grayscale). All animals chose Offer 2 more often when it delivered reward earlier (bottom right, light) and less when it delivered reward later (top left, dark). *Right:* overall probability of choosing an offer as a function of Tout. Same as in the main text figure. All monkeys preferred earlier reward delivery. (B) Same as in the main text figure, for each animal. Red data points at right quantify the effect of Tadvance on Info preference, quantified using the comparison in the right panel (i.e. ((Info, long Tadvance) – (Noinfo, long Tadvance)) – ((Info, short Tadvance) – (Noinfo, short Tadvance))). While each animal had a different average attitude toward Info vs Noinfo offers (animal R, slight Info preference; animal Z, strong Info preference; animal B, slight Info aversion), all animals were relatively more willing to choose Info when the cue came early in advance of the outcome (all red comparisons on line plots, $p < 0.001$; all effects of Tadvance effects > 0, $p < 0.001$, signed-rank tests).

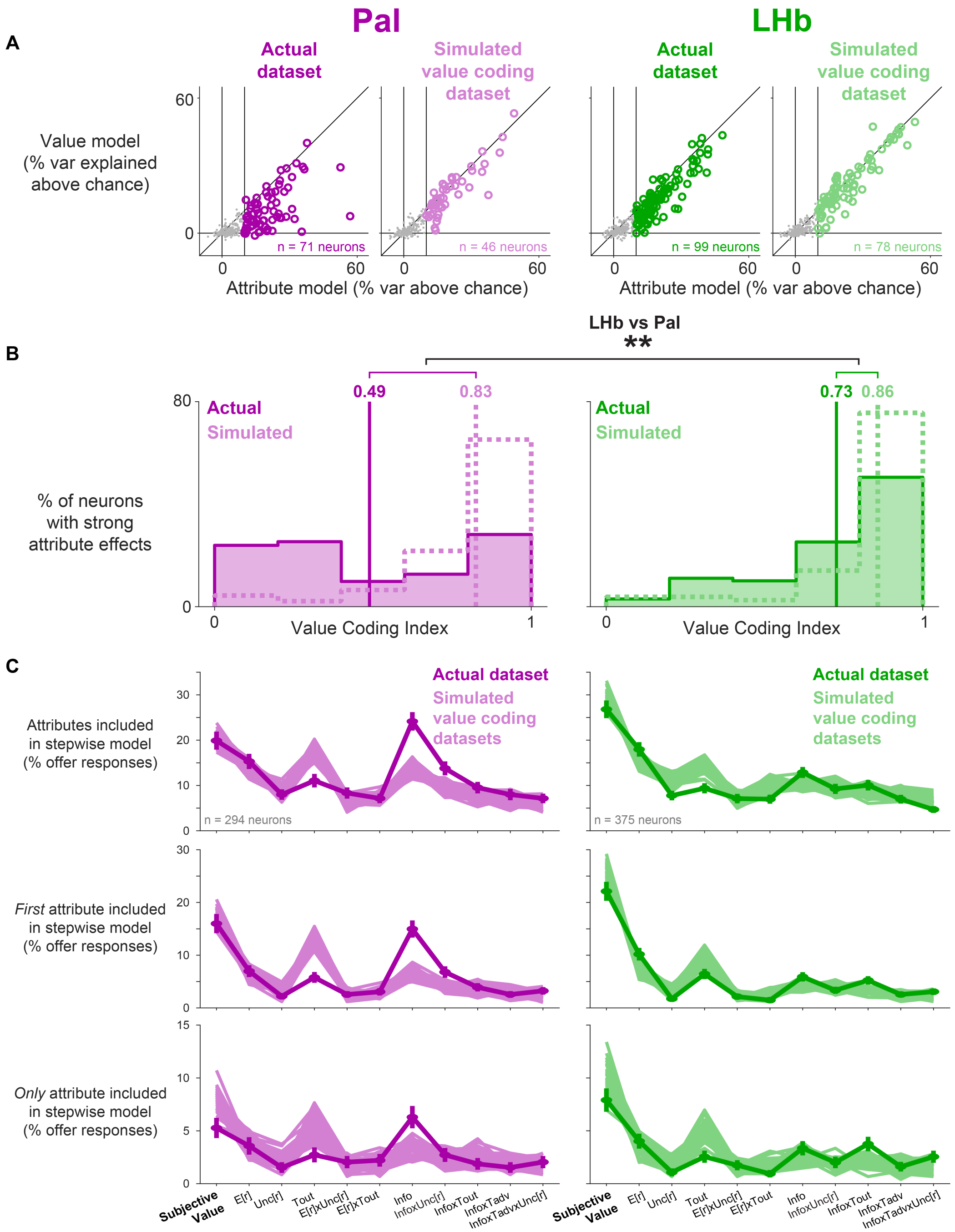


**Figure S14. Validation of prominent subjective value coding in LHb using simulated data.** (A) Evaluation of Pal (left, purple) and LHb (right, green) neuron activity using the Attribute Model vs. Value Model (scatterplots, same format as in main figure), on the real dataset (dark, left side of panels), and a simulated dataset representing the hypothesis that all neurons purely encoded subjective value (light, right side of panels; Methods).

(B) Same, for the Value Coding Index (histograms, same format as in main figure), showing the real data (dark solid lines) vs the simulated data (light dashed lines). Colored horizontal lines indicate difference between mean indexes for real vs simulated data; black horizontal line and asterisks indicates comparison between those differences for Pal vs LHb. The real LHb neurons had a considerably overlapping scatterplot and histogram to the simulated neurons, consistent with LHb neuron offer responses prominently encoding attributes in a manner resembling subjective value. The real LHb neurons had a somewhat smaller mean Value Coding Index than the simulated neurons (0.73 vs 0.86, $p < 0.001$, rank-sum test; n=99 neurons for actual data, n=78 simulated data), which

could be due to small additional attribute-related influences on their activity, or due to limitations of our models or simulations in representing subjective value. By contrast, the real Pal neurons had a broader scatterplot and much lower and more uniform distribution of Value Coding Indexes than the simulated neurons (0.49 vs 0.83, $p < 0.001$, rank-sum test; n=71 neurons for actual data, n=46 simulated data). This is consistent with Pal neurons integrating attributes in a variety of manners rather than all purely encoding subjective value. Thus, the real and simulated indexes were much more similar in LHb than Pal (difference between real and simulated indexes = 0.13 in LHb vs 0.34 in Pal; $p = 0.006$, permutation test).

(C) Stepwise model analysis of subjective value. We fit each neuron's response to each offer using a stepwise procedure (the matlab function "stepwiseglm"). In this procedure, attributes are added to the model one by one in order of how much they improve the fit, as long as adding each attribute significantly improves the fit ($p < 0.05$). Then terms are removed as long as this has little significant effect on the fit ($p > 0.10$). Then this process is repeated until no attributes can be added or removed. For this fit, we used all the attributes from the standard model (Model 8), plus an additional attribute "Subjective Value" representing the total subjective value of the offer according to the standard model. The rationale is that if neurons purely encode subjective value rather than any other combination of attributes, then compared to all other attributes, Subjective Value should be more commonly (1) *included* in model fits, (2) the *first* attribute included in model fits, (3) the *only* attribute included in model fits. Indeed, this was the case for LHb (left, dark green lines; n=375 neurons). However it was not the case for Pal, where the Info attribute was included at similar or higher rates than the Subjective Value attribute (right, dark purple lines; Info was included more often, was included first similarly often, and was the only attribute more often; n=294 neurons). Data are mean ± SE.

Of course, even if all neurons purely encoded subjective value, the stepwise procedure would not always prioritize the Subjective Value attribute for every fit to every offer response (e.g. due to estimation noise from the limited size of the dataset). Hence, we compared the results for the real dataset (dark lines) with the results from simulated datasets representing the hypothesis that all neurons purely encoded subjective value (light lines, one for each of n=50 simulated datasets; Methods). This showed that the LHb data was roughly similar to what one would expect if all neurons purely encoded subjective value (green, dark vs light), while the Pal data was distinctly different (purple, dark vs light; much greater representation of the Info attribute than expected).

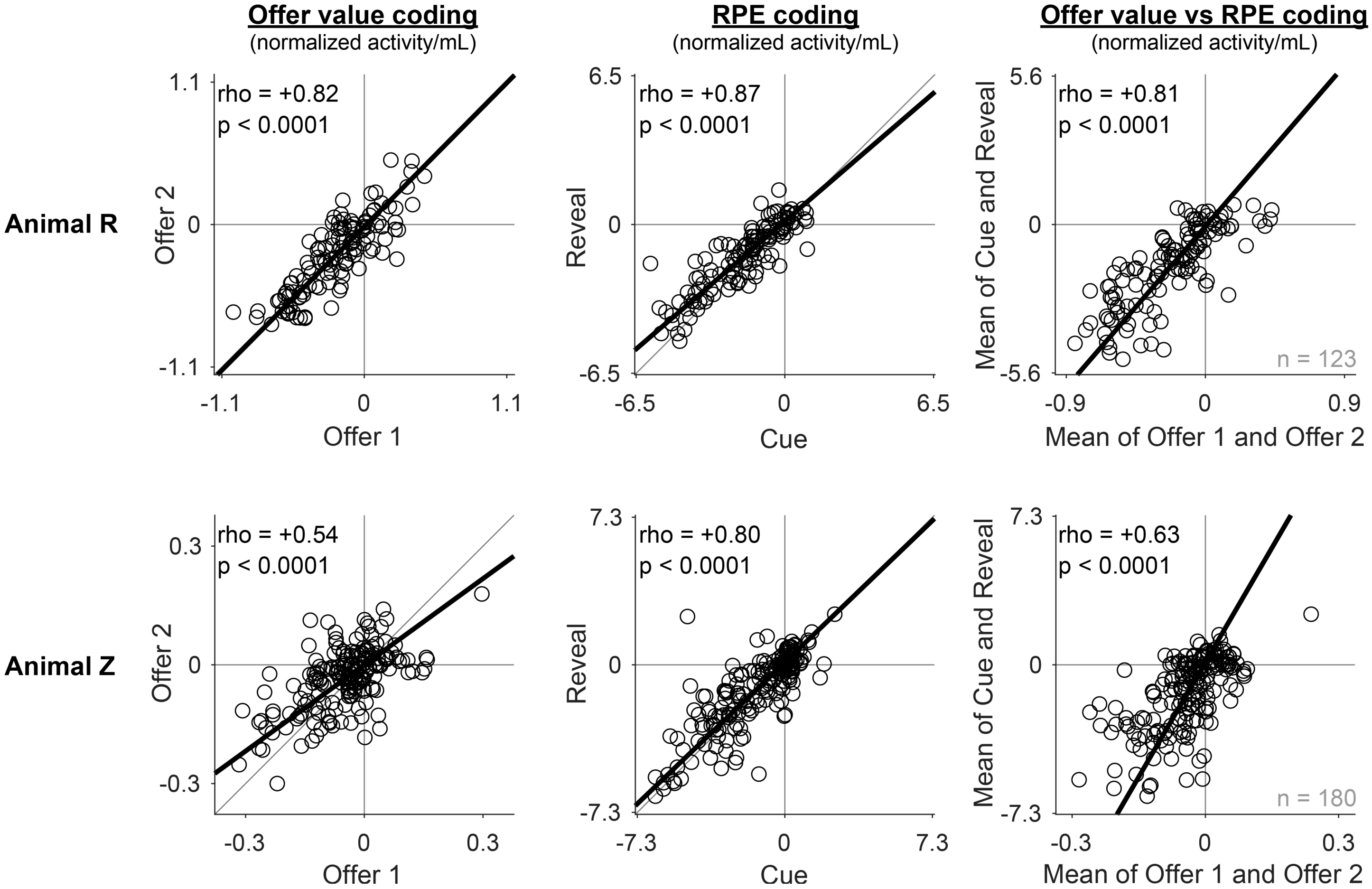


**Figure S15. Strength of LHb offer value signals vs RPE signals.** Rows show attribute-responsive neurons from Animal R and Z (top and bottom). Columns show estimates of the strength of offer value signals (left, Offer 1 and Offer 2), RPE signals (middle, Cue for Info offers and Reveal for Noinfo offers), and a comparison of the two (right, mean Offer 1/2 signal vs mean Cue/Reveal signal) for each neuron (dots). Gray lines in left and middle columns indicate the identity line. Black thick lines indicate the best linear fit (type 2 regression), text indicates rank correlation and its p-value.

To estimate the strength of neural signals, we used simple linear regression of each neuron's normalized activity in response to each offer with the subjective value of that offer (in units of log odds of choice), and of each neuron's normalized activity in response to cues and reveals with the RPE it produced (actual reward – predicted reward, in units of mL juice). To put offer value and RPE signals in common units, we then converted the offer value signal from units of log odds of choice into units of mL juice, by multiplying by the behavioral model's estimate of how much the log odds of choice would increase if the juice amount was increased by +1 mL.

This revealed that most neurons carried all of these signals in highly consistent manners. Offer value signals were highly consistent between Offer 1 and Offer 2 (left column). RPE signals were highly consistent between Info offer cues and Noinfo offer reveals (middle column). Offer value and RPE signals were highly correlated with each other (right column). This replicated in both animals (rows).

Offer value signals were smaller than RPE signals. The change in normalized activity per mL of juice was approximately 6.8x smaller (animal R) or 37.5x smaller (animal Z) for offers than for cues/reveals (slope of type 2 regression lines in right column). This could be for at least two reasons. First, temporal discounting. The subjective value per mL juice should be smaller for offers, because there is a much longer time delay between offer onset and reward delivery, than between cue/reveal onset

and reward delivery. Second, probability discounting. Only one of the two offers can be chosen on each trial, so each offer represents a possible outcome, whereas each Info trial cue and each Noinfo trial reveal indicates the actual outcome.

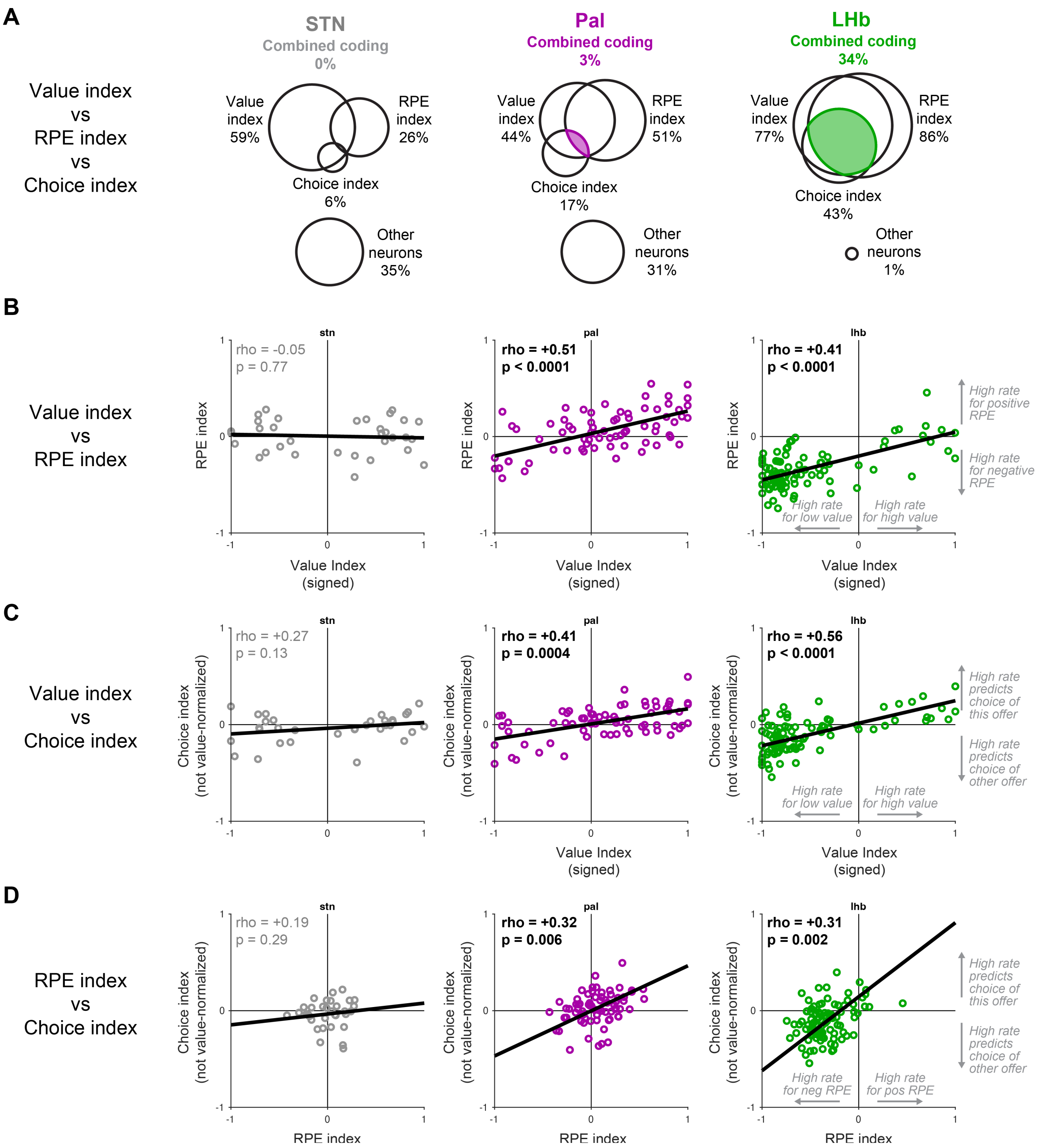


**Figure S16. Value Coding, RPE Coding, and Choice Predictive Indexes are correlated in Pal and LHb, consistent with partial and full integration, but not STN.** (A) Venn diagrams indicating overlap of the three coding indexes. Same as in Fig 4. Only LHb has a substantial population of neurons with a strong Value index, a significant RPE index, and significant Choice index, that all have the same coding sign. (B,C,D) Correlation of Value Coding Index with RPE Coding Index (B), Value Coding Index with Choice Predictive Index (C) and RPE Coding Index with Choice Predictive Index (D). All of these were highly significant in LHb and Pal, but not STN. This analysis was restricted to the subset of neurons for which all three indexes could be validly computed (n=33, 71, and 97 for STN, Pal, and LHb, respectively). Text indicates significance of rank correlation and its p-value. The line is the best linear fit by type 2 regression. For this analysis, to allow a direct comparison of the three indexes that can be interpreted in terms of changes in the neuron's firing rate, all indexes were adjusted so that positive indexes indicate that higher firing rates were associated with higher offer values, more positive RPEs, and greater choice, respectively. Specifically, the Value Coding Index was multiplied by the sign of each neuron's value coding effect (i.e. the sign of the mean of the fitted weight for the offer value term of

the Value Models; models 17 and 18, Table S3), so that a positive value index in this plot means that higher firing rate was associated with higher offer value, while a negative value index means that higher firing rate was associated with lower offer value. Similarly, the Choice Predictive index for this analysis was computed as the simple correlation between residual normalized activity and residual choice (i.e. without converting residual normalized activity into a residual value signal by multiplying by the neuron's sign of value coding, as was done in the main text), so that a positive index means that higher residual firing rate in response to an offer was associated with greater residual choice of the offer, while a negative index means that it was associated with lower residual choice of the offer. To incorporate data from both offers, the Choice Predictive Index for this analysis was computed by separately computing the indexes for offer 1 and offer 2 and then averaging the two.

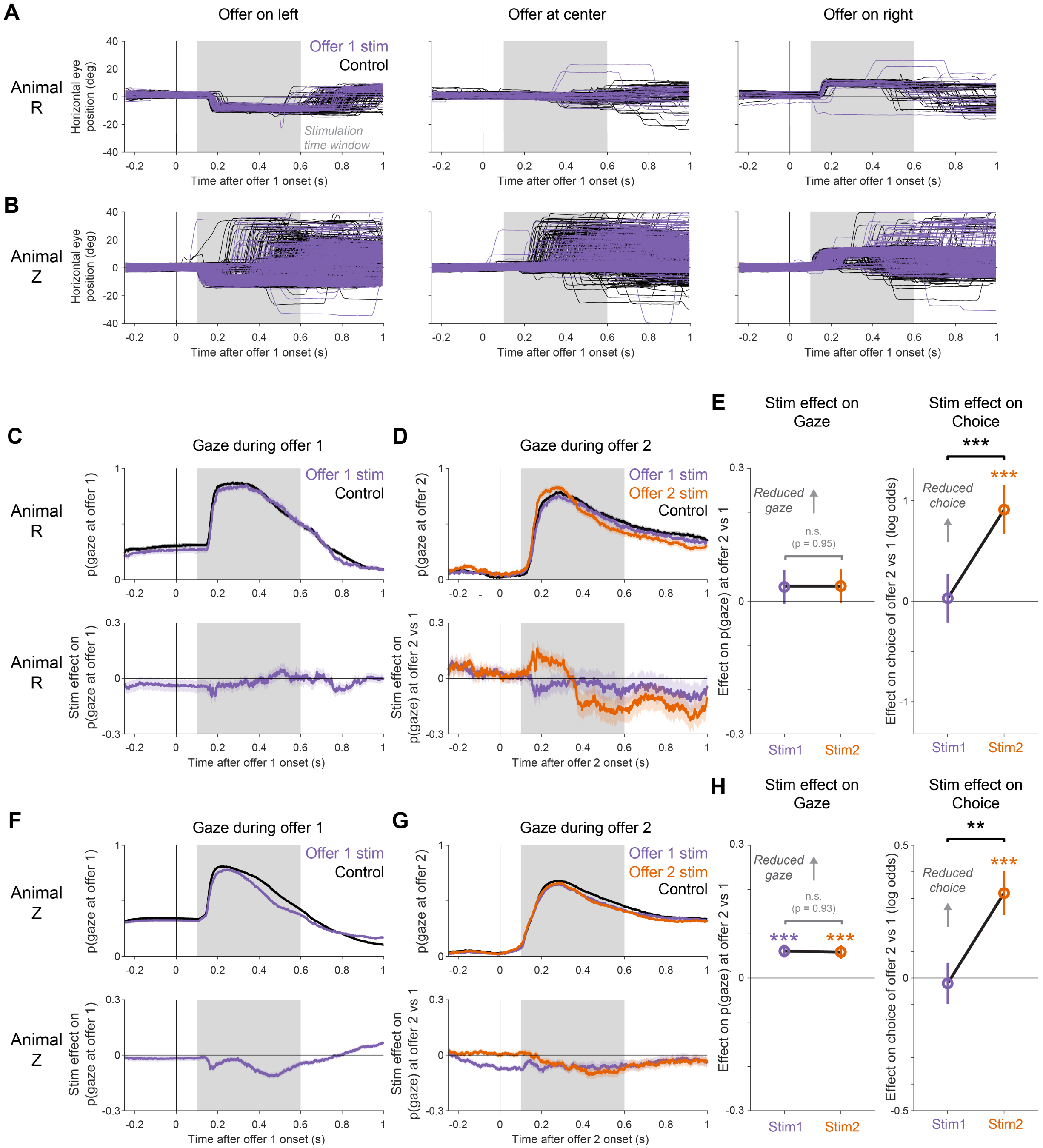


**Figure S17. LHb stimulation does not produce motor commands to control gaze, and the causal effect of stimulation on choice cannot be explained by an effect of stimulation on gaze.**

(A-B) LHb stimulation does not produce motor commands that directly interfere with choice in our task. If stimulation did so, then it would produce stereotyped perturbations in gaze that are tightly time-locked to stimulation onset, as is commonly observed in oculomotor areas (e.g. superior colliculus and frontal eye fields[109,110]). Furthermore, to interfere with choice, these perturbations would need to be both prominent and prolonged long after stimulation offset. For example, if stimulation induced contraversive eye movements, then it should induce a prominent rightward deviation in eye trajectories (since the electrode was located in the left LHb (Fig S7)). To test this, we compared eye position traces before, during, and after offer onset from single trials when the LHb was stimulated during offer presentation (purple) vs. a set of control trials in which no stimulation was performed during that offer (black; two control trials are shown for each stimulated trial). To make a direct comparison between full eye movement trajectories, this plot focuses on Offer 1 (to ensure

the eye always began at the same position, i.e. the fixation point at the center of the screen before offer onset); shows the horizontal component of eye position (since the three possible offer locations were spaced horizontally on the screen); and shows all trials which produced continuous gaze trajectories (uninterrupted by blinks). Similar results were observed for both offers, for both horizontal and vertical eye positions, and when examining blinks.

(A) Horizontal eye position during trials in which stimulation was performed during Offer 1 (purple curves) and control trials (black curves) in animal R, separately for offers presented on the left, middle, or right side of the screen (left, middle, right). Shaded area indicates the stimulation period. There was high overlap between the eye movement trajectories in stimulation and control conditions, including both initial saccades to the offer and later saccades away from the offer to empty locations on the screen. Stimulation did not induce obvious gross or stereotyped deviations from the natural variations of gaze shifts.

(B) Same, for Animal Z. Again, there was high overlap between stimulation and control conditions. Stimulation did not induce obvious gross or stereotyped deviations from the natural variations of gaze shifts.

(C-H) The causal effect of LHb stimulation on choice cannot be explained by an effect of stimulation on gaze. Specifically, while LHb stimulation did not have a direct motor-like effect on gaze, in some animals and conditions it had a modest tendency to influence the probability of gazing at the offer during and after stimulation, perhaps consistent with a change in the perceived motivational importance of the stimulus (C,D,F,G). Importantly, however, the effect on gaze alone could not explain the effect on choice: *choice* of Offer 2 vs Offer 1 was much more strongly influenced by stimulation during Offer 2, while *gaze* at Offer 2 vs Offer 1 was influenced similarly by stimulation during either offer (E,H).

(C) Testing for an effect of Offer 1 stimulation on gaze during Offer 1 in animal R. *Top:* probability of gazing at Offer 1 (defined as gaze in a 6° x 7.5° rectangular window around the center of the offer) at each time before and after Offer 1 onset, separately for trials with Offer 1 stimulation (purple) and the remaining trials (black). *Bottom:* estimated effect of Offer 1 stimulation on gaze at Offer 1 (purple), measured as the difference in gaze probability between the stimulation and control conditions above. Shaded areas are mean ± SE. There was little or no effect of stimulation on gaze during Offer 1.

(D) Testing for effects of Offer 1 and Offer 2 stimulation on gaze during Offer 2 in animal R. *Top:* probability of gazing at Offer 2 at each time before and after Offer 2 onset, separately for trials with Offer 1 stimulation (purple), Offer 2 stimulation (orange), and the remaining trials (black). *Bottom:* estimated effect of stimulation on gaze bias between the two offers. We measured gaze bias as the difference in the probability of gazing at the two offers (p(gaze at Offer 2) – p(gaze at Offer 1)). We then estimated the stimulation effect as the difference between the gaze bias in each stimulation condition vs. the control condition, and quantified it as the mean effect in the stimulation window (gray shaded area). Shaded areas are mean ± SE. There were trends for Offer 1 stimulation to make gaze at Offer 2 slightly less likely, and for Offer 2 stimulation to make gaze at Offer 2 initially slightly more likely and later slightly less likely. However, neither effect reached significance (i.e. the bootstrap 95% CIs did not exclude 0).

(E) Stimulation effects on gaze cannot explain stimulation effects on choice in animal R. *Left:* effect of stimulation during Offer 1 vs Offer 2 on gaze bias between Offer 1 vs Offer 2. This shows the same effects that were illustrated in (D), quantified here as the mean effect on p(gaze) during the stimulation window (gray shaded area), and plotted so that positive effects indicate a gaze bias against Offer 2. There were no significant effects of either Offer 1 or Offer 2 stimulation, and no significant difference between them (no bootstrap 95% CIs excluded 0). Data are fitted parameters ± SE. *Right:* effect of the same stimulations on the same trials on

choice bias between Offer 1 vs Offer 2. This is the same plot as in Figure 5, quantifying choice bias using the fitted GLM in terms of the log odds of choosing Offer 1 vs Offer 2, plotted so that positive effects indicate a choice bias against Offer 2. There was no significant effect of Offer 1 stimulation, a highly significant effect of Offer 2 stimulation, and a highly significant difference between them.

(F-H) Stimulation effects on gaze cannot explain stimulation effects on choice in animal Z. Same as (C-E), for animal Z. (F) There was a modest effect such that Offer 1 stimulation slightly reduced the probability of gazing at Offer 1 (bootstrap 99.9% CI excluded 0). (G) There was a modest effect such that either Offer 1 stimulation or Offer 2 stimulation slightly reduced the probability of gazing at Offer 2 (bootstrap 99.9% CIs excluded 0). (H) Stimulation effects on gaze were not significantly different between Offer 1 and Offer 2 stimulation (left), but stimulation effects on choice were significantly much greater for Offer 2 stimulation (right). *, **, *** indicate that the 95%, 99%, or 99.9% bootstrap CIs excluded 0 (left) or that the GLM regressor was significant at $p < 0.05$, 0.01, or 0.001, respectively.

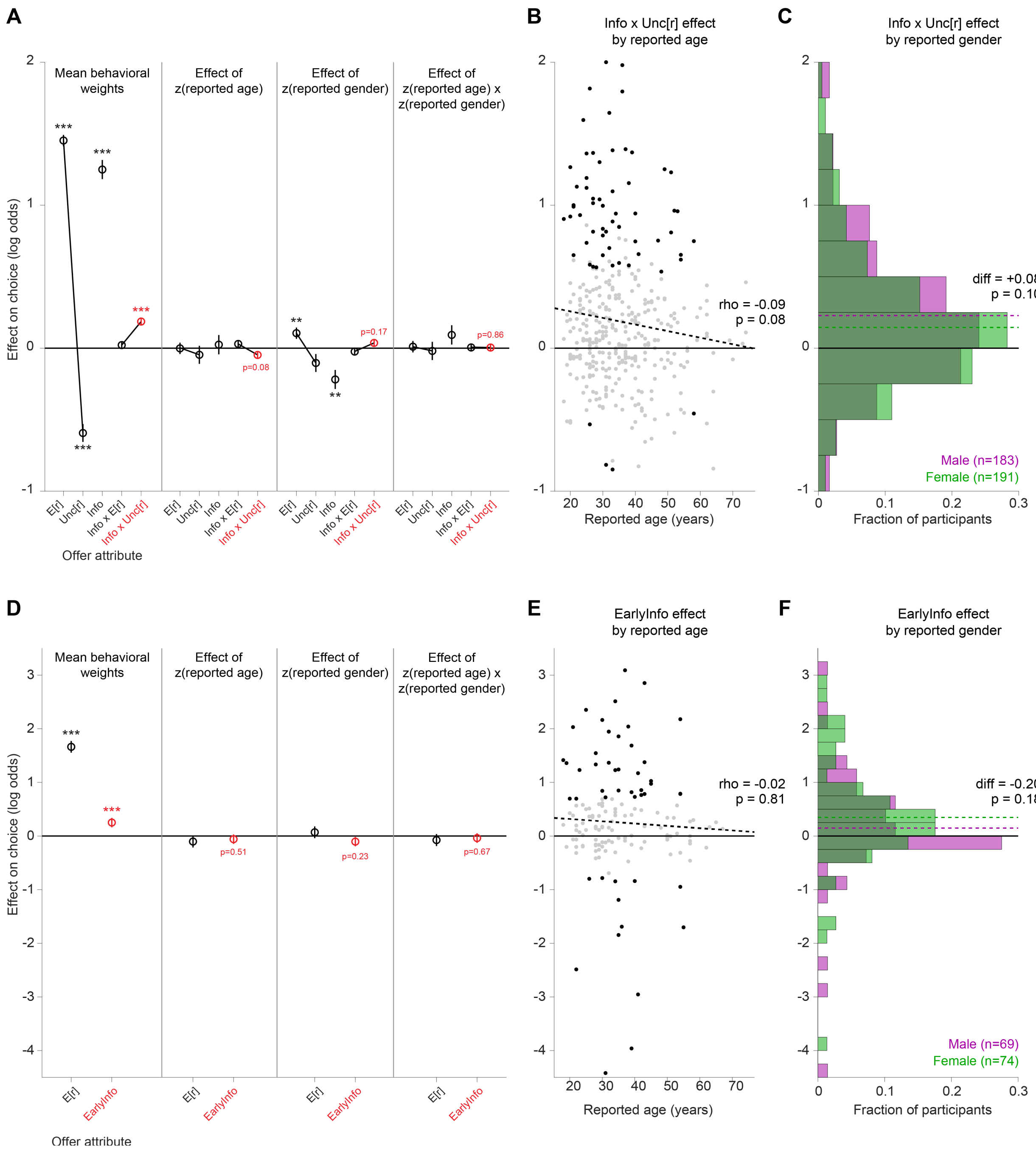

**Figure S18. Relationship between reported age, reported gender, and information seeking.** (A) Model of effects of reported age and reported gender on behavior in the first version of the human task. This model was fit to all individuals who reported that offer color indicated informativeness (see Figure S6,S7), reported an age, and reported themselves as male or female, and for whom the model converged (i.e. was able to produce a valid fit; n=374 participants). *Column 1:* mean fitted effects on each attribute on log odds of choice, using the standard model for the first version of the human task (Model 1). Data are mean ± SE. *Columns 2-4:* Then, for each attribute, we used standard linear regression to model the per-participant effects of that attribute as a linear combination of a constant term, participant's z-scored reported age, participant's z-scored reported gender (0 for female, 1 for male), and the interaction (product) of the two. Data are fitted parameters ± SE. *Column 2:* fitted effect of z-scored reported age on the fitted effects of each attribute on choice. There was no significant effect of age on any attribute's effect on choice. The closest was a trend for lower Info x Unc[r] interactions in participants with higher reported ages (red, p = 0.08). *Column 3:* fitted effect of z-scored reported gender. There were modest but significant effects for E[r] (higher in males, p = 0.0079)

and Info (higher in females, $p = 0.0011$). There was no significant effect for Info x Unc[r] (red, $p = 0.17$). Column 4: fitted effects of the interaction, which were all near-zero and non-significant (all $p > 0.15$). **(B)** Relationship between reported age (x-axis) and the fitted Info x Unc[r] effect on choice (y-axis). Black dots are significant along the y-axis ($p < 0.05$). Dashed line is best fit from type 2 regression, text indicates rank correlation and its p-value. There was a non-significant trend for lower Info x Unc[r] for older participants. **(C)** Relationship between reported gender (histogram color, purple for male and green for female) and Info x Unc[r] effect on choice (y-axis). Dashed lines indicate mean for each reported gender, text indicates difference and non-parametric p-value for difference of medians (rank-sum test). For clarity, the y-axis in (B,C) is cropped by excluding one outlier (a 32 year old female participant with Info x Unc[r] effect = -3.72). **(D,E,F)** Same as A,B,C but for the second version of the human task, which manipulated information timing (n=143 participants). **(D)** There were highly significant effects of E[r] and EarlyInfo on choice (Column 1, black and red) and these effects were not significantly different based on reported age (Column 2), reported gender (Column 3), or their interaction (Column 4). **(E)** No significant correlation between reported age (x-axis) and the EarlyInfo effect on choice (y-axis). **(F)** No significant relationship between reported gender (color of histograms) and the EarlyInfo effect on choice (y-axis).

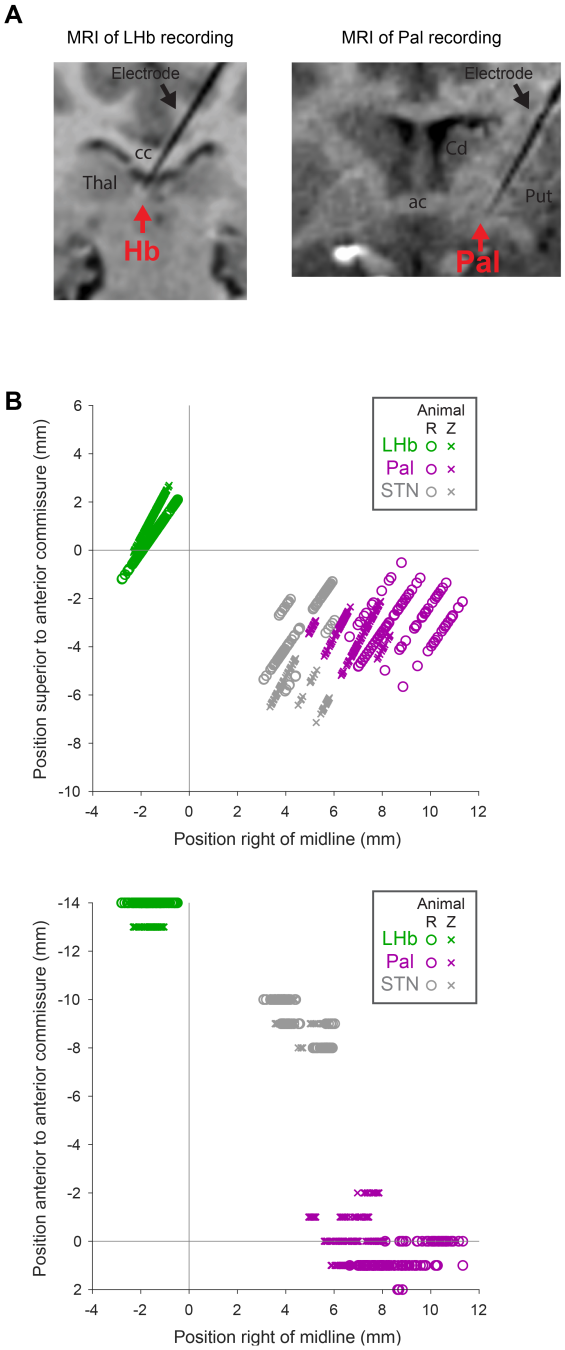


**Figure S19. MRI verification and anatomical locations of recording sites.**

(A) MRIs taken immediately after recording with the electrode still in place, verifying its location in the target area. Shown are coronal views slightly tilted to align with the electrode track. The electrode is visible as a black 'shadow' on the MRI. Black arrows indicate the electrode. Red arrows indicate the target area. Left: a recording site in LHb. Right: a recording site in Pal. Abbreviations: Hb, habenula; Pal, pallidum; cc, corpus callosum; Thal, thalamus; ac, anterior commissure; Cd, caudate nucleus; Put, putamen.

(B) Anatomical locations of recording sites. Reconstructed 3D coordinates of each neuron in the dataset, shown for all areas (indicated by colors: LHb, Pal, and STN as green, purple, and gray, respectively) and animals (indicated by symbols: circles and crosses for animals R and Z, respectively). Coordinates are relative to the midline, superior tip of the anterior commissure. *Top* shows coordinates in the coronal plane. *Bottom* shows

coordinates in the horizontal plane. The coordinates for each area were anatomically distinct from each other and were similar in both animals.

| | | | | | | P(reward distribution type) | | | | | |
|---|---|---|---|---|---|---|---|---|---|---|---|
| Animal | Task | #trials | E[r] off 1 | E[r] off 2 | Range | Safe | 25/50/25 | 50/50 | 25/75 | Rstep | Rmax |
| R | 1 | 10295 | 0.14-0.46 | +/- 0.24 | 0-0.6 | 0.11 | 0.33 | 0.33 | 0.22 | 0.02 | 0.6 |
| Z | 1 | 6159 | 0.15-0.55 | 0.15-0.55 | (0.35,0.5) | 0.33 | 0.33 | 0.33 | | 0.025 | 0.7 |
| B | 1 | 21038 | 0.14-0.46 | +/- 0.24 | 0-0.6 | 0.11 | 0.33 | 0.33 | 0.22 | 0.02 | 0.6 |
| P | 1 | 15116 | 0.28-0.85 | +/- 0.32 | 0-1.1 | 0.11 | 0.33 | 0.33 | 0.22 | 0.0375 | 1.125 |
| R | 2 | 40434 | 0.42-0.78 | 0.42-0.78 | (0.6,0.84) | 0.33 | 0.33 | 0.33 | | 0.04 | 1.2 |
| Z | 2 | 87491 | 0.48-0.72 | +/- 0.1 | (0.6,0.84) | 0.33 | 0.33 | 0.33 | | 0.04 | 1.2 |
| B | 2 | 4352 | 0.28-0.92 | +/- 0.5 | 0.3-1.2 | 0.33 | 0.22 | 0.22 | 0.22 | 0.04 | 1.2 |
| B | 2 | 16667 | 0.42-0.78 | +/- 0.35 | 0.4-1.2 | 0.33 | 0.22 | 0.22 | 0.22 | 0.04 | 1.2 |
| B | 2 | 13870 | 0.42-0.78 | 0.42-0.78 | (0.84) | 0.33 | 0.33 | 0.33 | | 0.04 | 1.2 |
| R | 2 w/stim | 1016 | 1.05-1.95 | 1.05-1.95 | (1.5,2.1) | 0.33 | 0.33 | 0.33 | | 0.10 | 3.0 |
| Z | 2 w/stim | 7822 | 0.48-0.72 | +/- 0.1 | (0.6,0.84) | 0.33 | 0.33 | 0.33 | | 0.04 | 1.2 |

**Table S1. Offer reward distribution statistics for all animals and sessions used in behavioral analysis.** Each row shows the parameter settings used to generate the offers while collecting a behavioral dataset after animals had been trained at the task and that was used in the main analysis of behavior. Separate data was used for analysis of behavior under normal conditions (tasks “1” and “2”) and in sessions with stimulation (task “2 w/stim”). Animal B’s behavioral dataset from task 2 included data collected with three slightly different task parameters as indicated by separate rows in the table; each other dataset from a given animal and task condition used a single set of parameters. All reward amounts and ranges are in units of mL juice. Columns indicate the animal’s identity; the task version (version 1 where offers showed reward distributions, or version 2 where offers also showed a clock); the number of correctly performed trials collected (only counting trials of the multi-attribute information task, not the interleaved simple information anticipation task if it was present in those sessions); the possible expected reward values for offer 1, indicated as a min-max range; the possible expected reward values for offer 2, indicated either as a min-max range (meaning that it was drawn independently of offer 1) or as “+/-“ (indicating that it was drawn from a range relative to the expected reward value of offer 1); the possible reward ranges for uncertain reward distributions, indicated either as a min-max range of possible reward ranges, or a set of specific reward ranges; the probability of the offer having each of the possible reward distribution types (Safe, 25/50/25, 50/50, or 25/75); and the settings of Rstep and Rmax.

| Model # | Human | Monkey | Model name | Shown in | UncTerm | E[r] | Tout | Unc[r] | E[r] x Tout | E[r] x Unc[r] | Info | Info x E[r] | Info x Tout | Info x Tadv | Info x Unc[r] | Info x Tadv x Unc[r] | Other |
|---|---|---|---|---|---|---|---|---|---|---|---|---|---|---|---|---|---|
| **1** | **1** | **1** | **Distrib SD** | **Fig 2,3** | **SD** | **1** | | **1** | | | **1** | **1** | | | **1** | | |
| 2 | 1 | 1 | Distrib Range | Fig 4 | Range | 1 | | 1 | | | 1 | 1 | | | 1 | | |
| 3 | 1 | 1 | Distrib Entropy | Fig 4 | Entropy | 1 | | 1 | | | 1 | 1 | | | 1 | | |
| 4 | 1 | 1 | Distrb None | Fig 4 | | 1 | | | | | 1 | 1 | | | | | |
| 5 | 1 | 1 | Distrib UncType | Fig 4 | * | 1 | | | | | 1 | 1 | | | | | 1 |
| 6 | | 1 | Distrib UncType Detailed | Fig S12 | * | 1 | | | | | 1 | 1 | | | | | 1 |
| | | | | | | | | | | | | | | | | | |
| **7** | **2** | | **Human Info Timing** | **Fig 5, S7** | | **1** | | | | | **1** | | | | | | |
| | | | | | | | | | | | | | | | | | |
| **8** | | **2** | **Clock SD** | **Fig 3-5** | **SD** | **1** | **1** | **1** | **1** | **1** | **1** | | **1** | **1** | **1** | **1** | |
| 9 | | 2 | Clock Range | Fig 3-5 | Range | 1 | 1 | 1 | 1 | 1 | 1 | | 1 | 1 | 1 | 1 | |
| 10 | | 2 | Clock Entropy | Fig 3-5 | Entropy | 1 | 1 | 1 | 1 | 1 | 1 | | 1 | 1 | 1 | 1 | |
| 11 | | 2 | Clock None | Fig 3-5 | | 1 | 1 | | 1 | | 1 | | 1 | 1 | | | |
| 12 | | 2 | Clock UncType | Fig 4 | | 1 | 1 | | 1 | | 1 | | 1 | 1 | | | 1 |
| 13 | | 2 | Clock SD with Info x E[r] | Fig 2 | SD | 1 | 1 | 1 | 1 | 1 | 1 | 1 | 1 | 1 | 1 | 1 | |
| 14 | | 2 | Clock Info x Time only | Fig 5 | SD | 1 | 1 | 1 | 1 | 1 | 1 | | 1 | 1 | | | |
| 15 | | 2 | Clock Info x Unc only | Fig 5 | SD | 1 | 1 | 1 | 1 | 1 | 1 | | | | 1 | | |
| 16 | | 2 | Clock Info main effect only | Fig 5 | SD | 1 | 1 | 1 | 1 | 1 | 1 | | | | | | |
| 17 | | 2 | Clock Simple Timing | Fig 5 | SD | 1 | 1 | 1 | | 1 | 1 | | | | 1 | | 1 |

**Table S2. Models used to fit behavior and/or neural activity to examine the attributes underlying the subjective value of information and reward.** Each row is one generalized linear model. Bold rows are the main models used for modeling the behavioral or neuronal data collected from each human or monkey task ("Distrib SD", "Human Info Timing", and "Clock SD"). Other rows are alternate models used for model comparisons or to analyze the influence of specific attributes. Columns indicate the model number; the human and monkey task versions whose data the models were used to fit; the model name; the figures where it was used; the uncertainty term (if any) used in the model, with * indicating models that did not use only a single term for uncertainty and instead had separate terms for separate types of uncertain reward distributions (as described in Methods); which offer attributes were included in the model (attributes marked with a "1" were included, and otherwise were not included); and whether other additional attributes were included. Specifically, as described in Methods, the "Distrib UncType", "Distrib UncType Detailed", and "Clock UncType" models had separate attributes for whether an offer had each of several specific types of uncertain reward distributions, as well as the interactions of those uncertainty types with Info; similarly, the "Clock Simple Timing" model had separate weights for whether an offer had each of several specific settings of (Tcue, Treveal) event timings. Finally, in addition to the attributes listed here, all neural and behavioral models included the appropriate set of bias terms, e.g. fits to neural activity included a constant term representing the mean or baseline firing rate (Methods).

| | Task | | | | Attributes | | | | | | | | | | | | | |
|---|---|---|---|---|---|---|---|---|---|---|---|---|---|---|---|---|---|---|
| Model # | Monkey Task | Stimulation | Model name | Shown in | **"Clock SD", offer 1** | **"Clock SD", offer 2** | **V1** | **V2** | **V2-1** | **LHbResid2-1** | **LHbResid1** | **LHbResid2** | **Stim2-1** | **Stim1** | **Stim2** | **Stim2 x (V2-1)** | **Stim2 x V2** | **Other** |
| 18 | 2 | | Attribute Model | Fig 6-7 | 1 | 1 | | | | | | | | | | | | |
| 19 | 2 | | Value Model | Fig 6-7 | | | 1 | 1 | | | | | | | | | | |
| | | | | | | | | | | | | | | | | | | |
| 20 | 2 | | ChoicePred | Fig 7-8 | | | | | 1 | 1 | | | | | | | | |
| 21 | 2 | | ChoicePred by Offer | Fig 7-8 | | | | | 1 | | 1 | 1 | | | | | | |
| | | | | | | | | | | | | | | | | | | |
| 22 | 2 | 1 | StimPred by Offer | Fig 8 | | | | | 1 | | | | | 1 | 1 | | | |
| 23 | 2 | 1 | StimPred None | Table S4 | | | | | 1 | | | | | | | | | |
| 24 | 2 | 1 | StimPred Stim2 | Table S4 | | | | | 1 | | | | | | 1 | | | |
| 25 | 2 | 1 | StimPred Stim2xV2-1 | Table S4 | | | | | 1 | | | | | | 1 | 1 | | |
| 26 | 2 | 1 | StimPred Stim2xV2 | Table S4 | | | | | 1 | | | | | | 1 | | 1 | |
| 27 | 2 | 1 | StimPred Stim2xAttrib | Table S4 | | | | | 1 | | | | | | 1 | | | 1 |

**Table S3. Models used to fit behavior and/or neural activity to examine the neural implementation of total subjective value.** Each row is one generalized linear model. Columns indicate the model number; the monkey task version whose data the models were used to fit; the model name; the figures where it was used; the attributes used in the model; and whether other attributes were used. In addition to the attributes listed here, all neural and behavioral models included the appropriate set of bias terms, e.g. fits to neural activity included a constant term representing the mean or baseline firing rate (Methods). The first two attributes listed in this table (“Clock SD, offer 1” and “Clock SD, offer 2”) indicate the use of all of the parameters from the “Clock SD” model (Table S2) separately for offer 1 and/or offer 2. The next three attributes, the values of the two offers (V1 and V2) and their difference (V2-1) were derived from fits of “Clock SD” model (Methods). For LHb neurons, the next three attributes, LHbResid1 and LHbResid2 and their difference LHbResid2-1, were derived from the residuals from fitting that model to the neuron’s responses to Offer1 and Offer 2, respectively (Methods). For each stimulation session, Stim1 and Stim2 indicated whether electrical stimulation was delivered during Offer1 and Offer 2, respectively.

Finally, “StimPred” models were used in the model comparison to determine the attributes which interacted with stimulation effects. The “StimPred Stim2xAttrib” model is marked as “other” because it is the result of a model selection procedure which iteratively adds parameters to the model based on whether they significantly improve the fit. Specifically, we used model comparison to ask if the LHb stimulation effect during Offer 2 could be fit as a main effect of stimulation (consistent with stimulation subtracting subjective value from the stimulated offer). Alternately, stimulation might have no effect; might interact with the value of the offer (e.g. larger effect for offers with high value); might interact with the difference between the values of the offers (e.g. larger effect when Offer 2 was higher value than Offer 1); or might interact with specific attributes of the offers (e.g. larger effect for Info offers). To test this for each animal, we fit the full dataset including all stimulation sessions with a set of models representing each of those possibilities (Table S3,S4). All models had the attribute (V2-V1), representing the difference in estimated value between the offers and computed in the manner listed above. The model “StimPred None” had no other attributes; the model “StimPred Stim2” and all subsequent models listed here had an attribute for Stim2; the model “StimPred

Stim2xV2-V1" also had an additional attribute for the interaction between Stim2 and the value difference V2-V1; and "StimPred Stim2xV2" instead had an additional attribute for the interaction between Stim2 and V2.

The final model "StimPred Stim2xAttrib" was formulated using iterative model selection to select the subset of possible interactions between Stim2 and specific attributes of Offer 2 that significantly improved the model's fit. We started with "StimPred Stim2" as the base model. We then modified versions of the base model, each of which was the same but with one additional attribute, representing the interaction between Stim2 and the corresponding one of the 10 original attributes from the main model for the second version of the task. If none of these modified models had a significant effect of the added attribute, we terminated the procedure. If at least one of the modified models had a significant effect of the added attribute, we selected the modified model whose added attribute had the lowest p-value, designated it as a the new base model, and repeated this procedure. We continued this procedure until it terminated, thus producing a model in which each added Stim2 x attribute interaction had significantly improved the fit.

| Animal | Model | Log likelihood | AIC | BIC | Shuffle-corrected log likelihood | 95% CI of shuffle-corrected log likelihood difference from Stim2 model | | CI excludes 0? |
|---|---|---|---|---|---|---|---|---|
| R | StimPred None | -356.7233 | 721.4466 | 741.1411 | 345.9049 | -18.9636 | -1.5942 | ** |
| R | StimPred Stim2 | -348.0694 | 706.1387 | 730.7568 | 353.8033 | 0.0000 | 0.0000 | |
| R | StimPred Stim2xV2-1 | -347.6026 | 707.2052 | 736.7470 | 354.0707 | -0.7970 | 4.9478 | |
| R | StimPred Stim2xV2 | -346.2558 | 704.5116 | 734.0534 | 355.1253 | -0.5970 | 6.4470 | |
| R | StimPred Stim2xAttrib | -344.8271 | 701.6542 | 731.1960 | 355.4650 | -0.3372 | 20.8822 | |
| | | | | | | | | |
| Z | StimPred None | -3379.3587 | 6766.7174 | 6794.5761 | 2040.6604 | -15.2393 | -0.9377 | ** |
| Z | StimPred Stim2 | -3372.6851 | 6755.3702 | 6790.1937 | 2047.1161 | 0.0000 | 0.0000 | |
| Z | StimPred Stim2xV2-1 | -3372.6172 | 6757.2343 | 6799.0225 | 2046.7735 | -0.8420 | 2.5533 | |
| Z | StimPred Stim2xV2 | -3372.6838 | 6757.3675 | 6799.1557 | 2046.3743 | -0.8630 | 1.8290 | |
| Z | StimPred Stim2xAttrib | -3370.3504 | 6752.7008 | 6794.4889 | 2048.0792 | -1.6781 | 17.2368 | |

**Table S4. The influence of LHb offer 2 stimulation on choice was predominantly explained by a main effect of stimulation.** Each row is one model fit (see Table S3 for full descriptions of all models). In brief, the “StimPred None” model does not include any stimulation effects; the “StimPred Stim2” model includes a main effect of stimulation during offer 2; and the further models additionally include interactions between stimulation and the value difference between the offers (“Stim2xV2-1”), the value of offer 2 (“Stim2xV2”), or a subset of offer 2 attributes selected via a stepwise model selection procedure to optimize the fit (“Stim2xAttrib”; Methods). Columns indicate the animal, model, log likelihood, AIC, BIC, shuffle-corrected log likelihood, and the 95% bootstrap confidence interval of the difference between the shuffle-corrected log likelihoods of the current model and the “StimPred Stim2” model. *, **, *** indicate that 0 was excluded by the 95%, 99%, or 99.9% bootstrap confidence intervals (computed with 10000 bootstraps). Comparing models with shuffle-corrected log likelihood was crucial to control for the fact that some models would be expected to have higher log likelihood even by chance (due to having more parameters, or due to the StimPred Stim2xAttrib model using an iterative procedure to select the subset of parameters that improve the fit and then only use those). In both animals, the fit was reliably improved by including the main effect of Stim2 (“StimPred Stim2” vs “StimPred None”), but was not reliably improved by including interactions between Stim2 and either offer values or specific offer attributes.

| | Task | | | Human fit | | | Monkey fit | | | LHb fit | | | Pal fit | | |
|---|---|---|---|---|---|---|---|---|---|---|---|---|---|---|---|
| Model # | Human | Monkey | Model name | Log likelihood | AIC | BIC | Log likelihood | AIC | BIC | Log likelihood | AIC | BIC | Log likelihood | AIC | BIC |
| **1** | **1** | **1** | **Distrib SD** | -34397.0 | 75573.9 | 85780.0 | -18607.7 | 37279.4 | 37515.7 | | | | | | |
| 2 | 1 | 1 | Distrib Range | -35315.2 | 77410.3 | 87616.4 | -18969.4 | 38002.8 | 38239.1 | | | | | | |
| 3 | 1 | 1 | Distrib Entropy | -36893.8 | 80567.7 | 90773.7 | -21149.0 | 42362.0 | 42598.3 | | | | | | |
| 4 | 1 | 1 | Distrb None | -40581.5 | 85683.0 | 92487.0 | -21468.8 | 42985.6 | 43162.8 | | | | | | |
| 5 | 1 | 1 | Distrib UncType | -32492.6 | 74025.2 | 87633.2 | -20510.2 | 41112.4 | 41453.5 | | | | | | |
| 6 | | 1 | Distrib UncType Detailed | | | | -18725.0 | 37622.1 | 38263.8 | | | | | | |
| **7** | **2** | | **Human Info Timing** | -10616.0 | 22911.0 | 25100.0 | | | | | | | | | |
| **8** | | **2** | **Clock SD** | | | | -69971.2 | 140020.4 | 140364.2 | -465012.7 | 949525.5 | 981075.5 | -342906.5 | 701049.0 | 724903.6 |
| 9 | | 2 | Clock Range | | | | -70469.0 | 141016.0 | 141359.8 | -465157.6 | 949815.2 | 981365.2 | -343039.3 | 701314.5 | 725169.2 |
| 10 | | 2 | Clock Entropy | | | | -72234.0 | 144546.1 | 144889.9 | -465397.2 | 950294.4 | 981844.4 | -343376.1 | 701988.2 | 725842.8 |
| 11 | | 2 | Clock None | | | | -74899.0 | 149851.9 | 150090.0 | -467176.9 | 947853.7 | 969696.0 | -345031.7 | 700611.4 | 717126.1 |
| 12 | | 2 | Clock UncType | | | | -70230.0 | 140570.0 | 141050.0 | -463450.0 | 952410.0 | 993670.0 | -341740.0 | 703410.0 | 734610.0 |
| 13 | | 2 | Clock SD with Info x E[r] | | | | -69874.0 | 139831.9 | 140202.2 | -464435.2 | 949870.4 | 983847.3 | -342504.1 | 701416.1 | 727105.7 |
| 14 | | 2 | Clock Info x Time only | | | | -72583.8 | 145233.6 | 145524.5 | -466123.0 | 948746.0 | 975442.1 | -343901.8 | 700695.6 | 720880.3 |
| 15 | | 2 | Clock Info x Unc only | | | | -70988.5 | 142037.0 | 142301.5 | -466319.6 | 947639.3 | 971908.5 | -344101.8 | 699923.6 | 718273.3 |
| 16 | | 2 | Clock Info main effect only | | | | -73417.4 | 146888.7 | 147126.7 | -467010.6 | 947521.3 | 969363.6 | -344744.9 | 700037.8 | 716552.5 |
| 17 | | 2 | Clock Simple Timing | | | | -70792.8 | 141657.6 | 141974.9 | | | | | | |
| 18 | 2 | | Attribute Model | | | | | | | -459528.9 | 956557.8 | 1017230.8 | -338803.7 | 706907.4 | 752781.7 |
| 19 | 2 | | Value Model | | | | | | | -469504.4 | 946508.9 | 958643.5 | -347594.5 | 701049.0 | 710223.8 |
| 20 | 2 | | ChoicePred | | | | -26951.2 | 56140.4 | 59766.0 | | | | | | |
| 21 | 2 | | ChoicePred by Offer | | | | -26745.1 | 56474.3 | 61308.4 | | | | | | |
| 22 | 2 | 1 | StimPred by Offer | | | | -3720.7 | 7465.4 | 7536.8 | | | | | | |
| 23 | 2 | 1 | StimPred None | | | | -3736.1 | 7488.2 | 7535.7 | | | | | | |
| 24 | 2 | 1 | StimPred Stim2 | | | | -3720.8 | 7461.5 | 7521.0 | | | | | | |
| 25 | 2 | 1 | StimPred Stim2xV2-1 | | | | -3720.2 | 7464.4 | 7535.8 | | | | | | |
| 26 | 2 | 1 | StimPred Stim2xV2 | | | | -3718.9 | 7461.9 | 7533.2 | | | | | | |
| 27 | 2 | 1 | StimPred Stim2xAttrib | | | | -3715.2 | 7454.4 | 7525.7 | | | | | | |

**Table S5. Model fit quantification for all models.** For each model, shown are the log likelihood, Akaike's Information Criterion (AIC), and Bayesian Information Criterion (BIC), separately for fits to human behavior, monkey behavior, and LHb and Pal neuronal activity. Quantities are summed over individuals, and for neuronal data are summed over fits to single neurons. Higher log likelihoods correspond to higher probability of the data given the model fit. For fits to the same data set, if one model has lower AIC than another then it is approximately estimated to have lower prediction error in a model comparison; if one model has lower BIC than another it is approximately estimated to have a higher Bayes Factor in a model comparison.

These results are consistent in many features with our findings in the main text. Notably, SD-based models have higher log likelihood and lower AIC and BIC than Range- or Entropy-based models, in human behavior, monkey behavior, LHb neurons, and Pal neuron. Similarly, monkey behavior during LHb stimulation has lower AIC and BIC as a simple main effect of stimulation during offer 2 ("StimPred Stim2") rather than by either simpler or more complex models. Similarly, the Attribute Model has higher log likelihood but lower AIC and BIC than the Clock SD model, as expected since the Attribute Model is the same as the Clock SD model but with more parameters (because it fits each offer response as a function of the attributes of both offers, in order to reduce any possible bias in estimates of residual neural activity, for the analysis relating residual activity to residual choice).

Note that neuronal data is analyzed using fits to single neurons, which have much less data than fits to full behavioral datasets. Hence model selection measures will favor neural models with many fewer parameters than the behavioral models (Methods). Indeed, for this data AIC and BIC appear to be most useful for selecting which models efficiently describe these small single neuron datasets, rather than which models accurately describe the full suite of attributes that govern the population's neural signals. For example, AIC and BIC favor overly simple models for single neuron fits (e.g. "Clock None" with no uncertainty effects, "Clock Info main effect only" with only a main effect of Info, and the "Value Model" for Pal which requires all attribute effects to be described by a single value parameter), even though our analysis in the main text of population activity and fitted parameters indicate that higher-order effects were strongly present in the neural populations and in

animal behavior (e.g. highly significant Info x Uncertainty and Info x Time effects in “Clock SD” models; highly significant attribute effects as distinct from value in the “Attribute Model” for Pal). Thus, these results indicate that very simple models can perform well for describing very small single neuron datasets, while models with additional parameters are crucial for uncovering how neural populations encode the motivational attributes that govern subjective value.

| **Figure** | **Panel** | **Test** | **Label** | **p** |
|---|---|---|---|---|
| 2 | b | sign-rank | E[r] | 0 |
| | | sign-rank | Unc[r] | 0 |
| | | sign-rank | Info | 0 |
| | | sign-rank | Info x E[r] | 0.0377 |
| | | sign-rank | Info x Unc[r] | 0 |
| | | sign-rank | E[r] > Unc[r] | 1.32E-83 |
| | | sign-rank | Info x Unc[r] > Info x E[r] | 5.29E-05 |
| 2 | d | t | E[r] | 0 |
| | | t | Unc[r] | 0 |
| | | t | Info | 0 |
| | | t | Info x E[r] | 0.0001 |
| | | t | Info x Unc[r] | 0 |
| | | t | E[r] > Unc[r] | 0 |
| | | t | Info x Unc[r] > Info x E[r] | 3.44E-88 |
| 2 | e | binomial | Higher E[r] | 0 |
| | | binomial | Higher Info x Unc[r] | 0.0054 |
| 2 | g | binomial | pos. Info x Unc[r] | 2.28E-06 |
| 2 | h | sign-rank | Risk avoiders | 0.001 |
| | | sign-rank | Risk non-sig | 0 |
| | | sign-rank | Risk seekers | 0 |
| 2 | j | t | Risk averse sessions | 0.000118 |
| | | t | Risk neutral sessions | 0.000001 |
| | | t | Risk seeking sessions | 0 |
| 3 | b | sign-rank | Info x Unc[r] | 0 |
| | c | binomial 1-tailed | Info | 0 |
| | | binomial 1-tailed | Info x E[r] | 1.59E-09 |
| | | binomial 1-tailed | Info x Unc[r] | 0 |
| | | sign-rank | Info x Unc[r] > Info x E[r] | 0.0406 |
| | e | sign-rank | Info x Unc[r] | 0 |
| | f | binomial 1-tailed | Info | 0 |
| | | binomial 1-tailed | Info x E[r] | 1.75E-05 |
| | | binomial 1-tailed | Info x Unc[r] | 0 |
| | | sign-rank | Info x Unc[r] > Info x E[r] | 0.0007 |
| 4 | c | sign-rank | Safe vs 25/50/25 | 0 |
| | | sign-rank | 25/50/25 vs 50/50 | 0.006 |
| | d | sign-rank | Safe vs 25/50/25 | 9.34E-07 |
| | | sign-rank | 25/50/25 vs 50/50 | 1.83E-04 |
| | f | sign-rank | Safe vs 25/50/25 | 0 |
| | | sign-rank | 25/50/25 vs 50/50 | 0.01 |
| | g | t | Safe vs 25/50/25 | 2.51E-27 |
| | | t | 25/50/25 vs 50/50 | 0.01 |
| | j | sign-rank | Safe vs 25/50/25 | 0.0212 |
| | | sign-rank | 25/50/25 vs 50/50 | 0 |
| | k | bootstrap CI | SD vs Range | > 99.9% |
| | | bootstrap CI | SD vs Entropy | > 99.9% |

| | | | | |
|---|---|---|---|---|
| | | bootstrap CI | SD vs None | > 99.9% |
| | m | sign-rank | Safe vs 25/50/25 | 0.0003 |
| | | sign-rank | 25/50/25 vs 50/50 | 0.0115 |
| | n | bootstrap CI | SD vs Range | > 99.9% |
| | | bootstrap CI | SD vs Entropy | > 99.9% |
| | | bootstrap CI | SD vs None | > 99.9% |
| 5 | c | bootstrap CI | Short vs Long (left panels) | > 99.9% |
| | | bootstrap CI | Short vs Long (right panel) | > 99.9% |
| | d | t | R, EarlyEarly vs EarlyLate | 0 |
| | | t | R, EarlyLate vs LateLate | 0 |
| | | t | Z, EarlyEarly vs EarlyLate | 0 |
| | | t | Z, EarlyLate vs LateLate | 0 |
| | | t | B, EarlyEarly vs EarlyLate | 0.000035 |
| | | t | B, EarlyLate vs LateLate | 0 |
| | h | sign-rank | Info x Time | 0.0006 |
| | i | binomial 1-tailed | All Info x Time | 0 |
| | | binomial 1-tailed | Info x Tout | 0.0005 |
| | | binomial 1-tailed | Info x Tadv | 0.0082 |
| | | binomial 1-tailed | Info x Tadv x Unc[r] | 0.0123 |
| | j | bootstrap CI | vs Info x Unc[r] | > 99.9% |
| | | bootstrap CI | vs Info main effect only | > 99.9% |
| | l | sign-rank | Info x Time | 0.00335 |
| | m | binomial 1-tailed | All Info x Time | 0 |
| | | binomial 1-tailed | Info x Tout | 0 |
| | | binomial 1-tailed | Info x Tadv | 0.0002 |
| | | binomial 1-tailed | Info x Tadv x Unc[r] | 0.0213 |
| | n | bootstrap CI | vs Info x Unc[r] | > 99.9% |
| | | bootstrap CI | vs Info main effect only | > 99.9% |
| 6 | c | Fisher's combination | Info cue | 0 |
| | | Fisher's combination | Noinfo reveal | 0 |
| | f | Fisher's exact | 2 LHb | 0 |
| | | Fisher's exact | 3 LHb | 0.000072 |
| | | Fisher's exact | 4 LHb | 0.001498 |
| | | Fisher's exact | 5 LHb | 0.004296 |
| | | Fisher's exact | 6 LHb | 0.307232 |
| | | Fisher's exact | 2 Pal | 0.000003 |
| | | Fisher's exact | 3 Pal | 0.001496 |
| | | Fisher's exact | 4 Pal | 0.012932 |
| | | Fisher's exact | 5 Pal | 0.118997 |
| | | Fisher's exact | 6 Pal | 0.283762 |
| | h | rank-sum | Animal R | 0.0001 |
| | | rank-sum | Animal Z | 0.0456 |
| | | rank-sum | Both animals | 0 |
| 7 | b | Spearman | rho | 0.0016 |
| | | rank-sum | ROC area | 0.005 |
| | c | Spearman | rho | 0 |

| | | | | |
|---|---|---|---|---|
| | | rank-sum | ROC area | 0.000002 |
| | e | sign-rank | population | 0 |
| | | binomial 1-tailed | significant positive cells | 0.0097 |
| | f | sign-rank | population | 0 |
| | | binomial 1-tailed | significant positive cells | 0 |
| | g | sign-rank | R 1 | 0 |
| | | sign-rank | R 2 | 0 |
| | | sign-rank | R 1 vs 2 | 0.0279 |
| | | sign-rank | Z 1 | 0.5715 |
| | | sign-rank | Z 2 | 0 |
| | | sign-rank | Z 1 vs 2 | 0.0014 |
| 8 | j | t | R 1 | 0.985198 |
| | | t | R 2 | 0.000105 |
| | | t | R 1 vs 2 | 0.000828 |
| | | t | Z 1 | 0.76922 |
| | | t | Z 2 | 0.000288 |
| | | t | Z 1 vs 2 | 0.00589 |
| | k | t | Value | 0 |
| | | t | LHbResid Offer1 | 0 |
| | | t | LHbResid Offer2 | 0 |
| | | t | LHbResid Offer1 vs Offer2 | 0.000703 |
| | l | t | Value | 0 |
| | | t | LHbStim Offer1 | 0.862425 |
| | | t | LHbStim Offer2 | 0.000002 |
| | | t | LHbStim Offer1 vs Offer2 | 0.000146 |

**Table S6. Detailed statistics from figures.** Each row is one statistical test or comparison. Columns indicate figure, panel, type of test or comparison, label of the relevant corresponding data from the figure panel, and p-value. All tests were two-tailed and not corrected for multiple comparisons unless otherwise noted. Very small p-values (all of which are < 0.001 or below) are indicated by "0". Comparisons made using confidence intervals are indicated as "> 99.9%", indicating that in those cases the 99.9% confidence interval excluded 0. Note that "t" indicates when fitted GLM weights were tested for significance using a standard approach with t statistics (as implemented by the default Matlab functions "glmfit" and "linhyptest") and "Spearman" indicates that Spearman's rank correlation was tested for significance using exact permutations or a standard large-sample approximation for its p-value (as implemented by the default Matlab function "corr")[111].

## Modeling Note

*Framework for modeling behavior and neural activity.* We fit each individual's binary choice data using GLMs designed to model a standard decision making formulation, in which values are computed for each offer and the resulting choice probability is a logistic function of their value difference:

$$\log \left(\frac{p(\text{choose offer 2})}{p(\text{choose offer 1})}\right) = V(\text{offer 2}) - V(\text{offer 1})$$

And the value of each offer *i* is a linear weighted combination of the offer's vector of n attributes $< x_{i,1}, x_{i,2}, \dots, x_{i,n} >$:

$$V(\text{offer } i) = \beta_1 x_{i,1} + \beta_2 x_{i,2} + \cdots + \beta_n x_{i,n}$$

Thus, the resulting model was a GLM for binomial data with a logistic link function, with the equation:

$$\log \left(\frac{p(\text{choose offer 2})}{p(\text{choose offer 1})}\right) = \beta_1 (x_{2,1} - x_{1,1}) + \beta_2 (x_{2,2} - x_{1,2}) + \cdots + \beta_n (x_{2,n} - x_{1,n})$$

For neuronal data we fit firing rates in response to the offers, with an analogous GLM for normal data with an identity link function (equivalent to ordinary linear regression):

$$Rate(\text{offer } i) = \beta_{i,0} + \beta_{i,1} x_{i,1} + \beta_{i,2} x_{i,2} + \cdots + \beta_{i,n} x_{i,n} + \varepsilon$$

…where $\beta_{i,0}$ is the neuron's mean or baseline response to offer *i* and ε is the error term. Note that we separately fitted each neuron's responses to offer 1 and offer 2.

For each analysis, we used models with attributes tailored to the behavioral task and analysis question at hand, producing a total of 27 models as described below (full formulation of models, at the end of this section, for full details and comparisons of all parameters used by all models). Some general procedures were consistent across models. First, to permit more interpretable comparisons between attributes, unless otherwise noted, all attributes that were not binary were standardized by z-scoring. Second, in addition to the terms described below related to offer values, all models also included terms for response biases. For monkey behavioral data, this included binary attributes for: if the offer was on the left side of the screen, if the offer was on the right side of the screen, if the offer was Offer 2. For monkey neuronal data, this included binary attributes for: if the offer was on the left side of the screen, if the offer was on the right side of the screen, and a constant term representing the neuron's average level of activity. No term for sequential order was included because the model was fit separately for responses to Offer 1 and Offer 2. For human data, this was one binary attribute for if the offer was on the right side of the screen. Third, in certain cases we used a fitted behavioral model to derive an estimate of the subjective values that an individual assigned to the offers. To do this, we simply plugged the fitted weights $< \beta_{i,1}, \beta_{i,2}, \dots, \beta_{i,n} >$ and that offer's attributes $< x_{i,1}, x_{i,2}, \dots, x_{i,n} >$ into the equation for $V(\text{offer } i)$ above. This produced an estimate of the subjective value that the individual assigned to each offer presented on each trial, in units of the offer's log odds of influence over the choice.

To compare a set of models and judge which fit best, we compared their log likelihoods. For human population data, the log likelihood was summed over individuals; for neuronal population data, it was

summed over neurons. We used a shuffling correction to control for the possibility that models could have higher likelihood simply due to having more parameters (or otherwise having more power to explain data by chance). Specifically, we fit each GLM on at least 10 shuffled versions of the dataset in which the variable to be predicted was shuffled randomly across trials, and then defined the shuffle-corrected log likelihood as the log likelihood from the fit to the real data minus the mean log likelihood of the fits to the shuffled datasets. To test whether a model had higher shuffle-corrected log likelihood than any other model, we repeated the above procedure 2000 times on 2000 bootstrap datasets generated by randomly resampling the original dataset's individuals (for human population data), trials (for single monkey data), or neurons (for monkey neuronal data) with replacement. We used this to calculate the bootstrap standard error of each model's shuffle-corrected log likelihood, and the 95% bootstrap confidence interval of the difference between the shuffle-corrected log likelihoods for each pair of models. One model was deemed better than another model if the confidence interval excluded 0. Model interpretations were also aided by tests of whether key parameters were significant or were significantly different from each other (using the linhyptest function in matlab).

*Model fits for the first version of the tasks (Figs 2-4).* The first set of analyses focused on behavior during the first version of multi-attribute information task for humans and monkeys. The standard, main model for this analysis (Model 1; Table S2) had attributes for expected reward (E[r]), reward uncertainty (Unc[r]), Informativeness (Info), and the interactions between Info and the other variables (Info x E[r] and Info x Unc[r]). In this set of analyses the Info attribute was centered at 0 by setting it to +0.5 for Info offers and -0.5 for Noinfo offers. We fit separate versions of the model using different hypothesized forms of uncertainty: standard deviation, range, and entropy[50], as follows:

$$SD[r] = \left(\sum_r p(r)(r - E[r])^2\right)^{0.5}$$

$$Range[r] = \max(r) - \min(r)$$

$$Entropy[r] = -\sum_r p(r)log_2(p(r))$$

As a control, we also fit a model without any uncertainty-related attributes. We then used the model comparison procedure described above to compare these models. The procedure consistently selected SD[r] as the best measure for both humans and monkeys, so we used SD[r] as the default uncertainty measure for all further models unless otherwise noted.

The second set of analyses was a more detailed characterization of the type of uncertainty that motivates information seeking. We fit models that replaced each uncertainty-related attribute (i.e. Unc[r] and Info x Unc[r]) with a set of separate attributes corresponding to the separate types of reward distributions used in the task. For example, in datasets with two uncertain reward distribution types, 25/50/25 and 50/50, the single Unc[r] attribute was replaced by two binary attributes indicating whether the offer was 25/50/25 and whether it was 50/50; similarly, the Info x Unc[r] attribute was replaced by two attributes that were the former two attributes multiplied by Info (e.g. the "Distrib UncType" model in Table S2). We also fit models that further subdivided each reward distribution type into offers of that type that were relatively high vs. low uncertainty (Fig S12, "Distrib UncType Detailed" model in Table S2).

*Model fits for the second version of the tasks (Figs 3-8).* The remaining analyses focused on tasks that manipulated event timing (the second version of the tasks) and on relating neuronal activity to behavior. For the human timing task, the behavioral model had two attributes, E[r] and EarlyInfo, where EarlyInfo indicated whether the offer yielded early information vs. late information. For the monkey timing task there were a

much larger number of possible variables to consider, since it included manipulations of both Tcue and Trew in addition to all of the other task variables described above. Therefore, we used a model selection procedure to identify the core set of attributes that most strongly governed the subjective value of offers across monkeys. We then used this as the standard, main model for the second version of the monkey task (Model 8; Table S2). The goal was to identify attributes that explained a large fraction of the variance in behavior in the very large behavioral datasets collected for each monkey, while still having a limited number of attributes so that the same model could be fit practically to the smaller datasets collected when recording each individual neuron. Importantly, these attributes were selected purely based on fits to behavioral data, independent of neuronal data. This selection procedure was performed on a preliminary behavioral dataset that included all three animals that provided data for the second version of the monkey task. In the procedure, we first started with a large set of possible attributes to include in the model (n=18), consisting of: E[r], SD[r], and Info; all pairwise interactions between those three variables; Tout and its pairwise interactions with those three variables; Tadvance and its pairwise interactions with those three variables; and all three-way interactions between Info, either Tout or Tadvance, and either E[r] or SD[r]. Then for each animal, we ranked the attributes by their importance in explaining behavior using an iterative procedure. We started by fitting the behavioral data with an empty model without any of those attributes (i.e. only including the standard bias terms), and measured its cross-validated log likelihood (10-fold cross-validation). We then iteratively added individual attributes to the model, at each step adding the single attribute that improved the cross-validated log likelihood the most, and stopping when no attributes produced improvements. Thus, for each animal, this produced a sequence of attributes ranked by their marginal importance in explaining choice behavior. This also placed lower and upper bounds on the ability of these models to explain the data, defined by the cross-validated log likelihood of the initial empty model vs. the final model. Finally, based on these results, we selected the subset of 10 attributes that best explained behavior across all animals. This corresponded to raising the cross-validated log likelihood from the lower bound up to ~99% of the way to the upper bound across all animals. Selecting a relatively small subset of impactful attributes was necessary in order to use the same model for both single neurons and behavior, to allow a direct comparison between behavioral and neuronal correlates of attributes and subjective value. This is because, while each animal's full behavioral dataset had thousands of trials, each single neuron's dataset only had approximately 80-400 trials, so having a small number of impactful attributes in the model was necessary for single neuron fits to be accurate and avoid overfitting. This produced the following 10 core attributes: E[r], Tout, SD[r], E[r] x Tout, E[r] x SD[r], Info, Info x Tout, Info x SD[r], Info x Tadvance, Info x Tadvance x SD[r]. We then defined a model with these attributes as the main model for the second version of the monkey task (the "Clock SD" model in Table S2), and used it as the basis for our analysis of both monkey behavior and neuronal activity in this task.

Specifically, just as we had done for the behavioral models of the first version of the task, we used the main model with the above attributes to fit data from the second version of the task. As before, we compared the model fit between the main model described above, which uses SD[r], versus alternate models using either Range[r], Entropy[r], or no uncertainty-related terms. Similarly, as before, we compared the fitted weights of the Info x SD[r] and Info x E[r] attributes to judge their relative effects; note that for this analysis we used an alternate version of the model that included Info x E[r] as an additional term, since the Info x E[r] interaction term was not identified as a core attribute by the procedure above (consistent with its relatively small effect on behavior in this task; Fig 2). Next, to investigate how information value is computed using uncertainty and time, we did a model comparison between the full model, a model that removed attributes with interactions between Info and Unc[r], a model that removed attributes with interactions between Info and time-related variables (Tout and Tadvance), and a model that removed attributes with both types of interactions.

Next, for a more detailed characterization of how information value depends on the timing of cues and outcomes, we fit a version of the model where all terms with interactions between Info and time-related

variables were removed, and instead, three attributes were included representing the interaction between info and the presence of specific timing properties in the offer (the "Clock Simple Timing" model in Table S2). Specifically, we defined cues as 'early' if Tcue was within the $0^{th}$-$33^{rd}$ percentiles of possible cue times, and 'late' if Tcue was within the $66^{th}$-$100^{th}$ percentiles of possible cue times. We also defined outcomes as 'early' and 'late' with an analogous rule. We then included three attributes to the model: Info x (early cue, early outcome), Info x (early cue, late outcome), and Info x (late cue, late outcome); where (x,y) was 1 for offers that met both the specified cue and outcome timing conditions, and 0 otherwise.

Finally, to compare across species, we computed the effect of Early vs Late Info on subjective value in animals and humans, in units of log odds of choice. To quantify this for each human, we used the fitted GLM weight of EarlyInfo. To quantify this for each monkey, we used the main model's GLM fit to derive the subjective value of each offer, and computed the mean difference in offer subjective value that resulted from info cues being presented early vs. late. To produce a measure a closely analogous as possible to the human data, this analysis only used the subset of offers that were most closely analogous to those used in human task (i.e. offers with high reward uncertainty and late outcome delivery, as defined above). We then defined the effect of interest as the difference between the mean derived value of Info offers vs. Noinfo offers with early cue delivery, minus the difference between the mean derived value of Info vs Noinfo offers with late cue delivery. Error bars and confidence intervals were computed with bootstrapping (n=2000 bootstraps).

*Analysis of peri-stimulus activity: Info x Uncertainty effect (Fig 3)*. To plot the cross-validated timecourse of normalized activity related to the interaction between Info and Uncertainty, we used the following procedure. First, separately for each attribute-responsive neuron and separately for Offer 1 and Offer 2, we classified all offers into the 2x2 categories of (Info, Noinfo) x (Uncertain, Safe). For offer *i*, we computed the raw Info x Uncertainty effect, $InfoXUnc_{i,raw}$ as:

$$InfoXUnc_{i,raw} = \big(Rate_i(Info, Uncertain) - Rate_i(Noinfo, Uncertain)\big) - (Rate_i(Info, Safe) - Rate_i(Noinfo, Safe))$$

…where $Rate_i$(*offer type*) is the neuron's mean normalized firing rate in the standard analysis window for responses to offer *i*, in response to the offers of the specified type. We then subjected these two offer-specific measures to two stages of cross validation to compute a the total Info x Uncertainty effect considering both offers. To do this, we first use cross-validation to sign-normalize the effect, so that positive values indicate any change in Info signals with Uncertainty, while zero values are expected under the null hypothesis of no Info x Uncertainty interaction. We did this by multiplying the sign of each offer's effect by the sign of the *other* offer's effect, using the equation:

$$InfoXUnc_i = InfoXUnc_{i,raw} \times \mathrm{sign}\big(InfoXUnc_{j,raw}\big)$$

…where *i* is the currently considered offer and *j* is the other offer. Thus, under the null hypothesis that the neuron does not have a true effect, $InfoXUnc_i$ has an expected value of 0 (because the two offer responses would have random effects and signs). Whereas if the neuron has a true Info x Uncertainty effect with a consistent positive sign, $InfoXUnc_i$ will be positive; and if it has a true effect with a consistent negative sign, $InfoXUnc_i$ will again be positive (because the negative effect for offer *i* would be multiplied by the negative sign of the effect for offer *j*, thus producing a positive result).

We then used a second cross-validation procedure to compute InfoXUnc, a measure of the total Info x Uncertainty effect for each neuron. This measure pools both offer responses, and selects only neural responses with significant Info x Unc[r] effects while using cross-validation to correct for selection bias, using the equation:

$$InfoXUnc = ((InfoXUnc_1 \times sig_2) + (InfoXUnc_2 \times sig_1))/\max(1, (sig_1 + sig_2)$$

…where $sig_i = 1$ if the neuron had a significant Info x Unc[r] effect in the model fits to its response to offer *i* ($p < 0.05$), and 0 otherwise. Neurons were only included in this analysis if $sig_1 = 1$ or $sig_2 = 1$. This cross-validation corrected for selection bias by ensuring that a neuron's response to offer *i* was selected for inclusion solely based on whether that neuron's response to offer *j* was significant, and vice versa. Crucially, we set a neuron's $sig_i = 1$ if it had a significant Info x Unc[r] effect in *any* of the three models fit to its offer response, that used distinct forms of uncertainty (defining Unc[r] as SD[r], Range[r], or Entropy[r], respectively (Fig 4, Models 8-10 ). This ensured that there was no bias favoring any one of the three hypothesized uncertainty measures. We then tested whether the median InfoXUnc effect across these neurons was different from 0 (signed-rank test). The above analysis was restricted to the mean normalized firing rates in the standard offer response window. We also plotted the full timecourse of the InfoXUnc effect by computing it using the full spike density functions from each neuron in each task condition (smoothed with a Gaussian kernel, σ = 40 ms), and while holding $sig_i$ and sign($InfoXUnc_{i,raw}$) fixed at the values used in the analysis above (so that the full timecourse of each neuron's activity was selected from a consistent subpopulation of neurons and normalized using a consistent sign).

*Analysis of peri-stimulus activity: Info x Uncertainty Type effect (Fig 4)*. To quantify the Info x Uncertainty Type interaction – that is, to test whether this Info x Uncertainty effect was stronger for uncertain offers with 50/50 or 25/50/25 reward distributions – we computed InfoXUncType in an analogous manner using the same neurons and neuronal responses. That is, we again used cross-validation to select the same subset of neurons with significant Info x Unc[r] effects, and to sign-normalize each neuron's activity in the direction of its Info x Uncertainty effect. This ensured that the InfoxUnc and InfoxUncType effects are directly comparable, in the sense that they represent changes in neuronal activity that occur in the same 'direction' across the population. That is, an increase in InfoXUnc and an increase in InfoXUncType represent the same pattern of excitation/inhibition across neurons). However, instead of computing how the Info effect differed between Safe and Uncertain offers, we computed how it differed between 50/50 and 25/50/25 offer types:

$$\begin{aligned} InfoXUncType_{i,raw} &= \big(Rate_i(Info, 50/50) - Rate_i(Info, 25/50/25)\big) \\ &\quad - \big(Rate_i(Noinfo, 50/50) - Rate_i(Noinfo, 25/50/25)\big) \\ InfoXUncType_i &= InfoXUncType_{i,raw} \times sign(InfoXUnc_j) \\ InfoXUncType &= ((InfoXUncType_1 \times sig_2) + (InfoXUncType_2 \times sig_1))/\max(1, (sig_1 + sig_2)) \end{aligned}$$

Thus, InfoXUncType should be positive if neural information signals are enhanced by an SD-like form of uncertainty (because the changes in activity are stronger for 50/50 offers), should be zero if they are enhanced by a Range-like form of uncertainty (because the changes are the same for 50/50 and 25/50/25 offers), and should be negative if they are enhanced by an Entropy-like form of uncertainty (because the changes are greater for 25/50/25 offers than 50/50 offers).

*Analysis of peri-stimulus activity: Info x Time effects (Fig 5)*. To plot the cross-validated timecourse of normalized activity related to the interactions between Info and time-related variables we used an analogous

procedure, to accommodate the fact that there were three different Info x Time attributes to measure (Info x Tout, Info x Tadvance, Info x Tadvance x Unc[r]), and that individual neurons might only have activity related to a subset of these effects (e.g. in Pal). Specifically, separately for each neuron and for Offer 1 and Offer 2, we fit the main model for the second version of the monkey task (Model 8) to the normalized activity in response to that offer, using the standard analysis time window for offer responses. We then used these fits to calculate a cross-validated measure of the summed strength of the fitted weights for subset of the Info x Time attributes that the neuron encoded, by performing cross-validation across the two offers to select which subset of attributes the neuron encoded and to select their coding signs. Specifically, we will denote the three attributes as attributes 1, 2, and 3; their fitted weights in response to each offer *o* as $w_{o1}$, $w_{o2}$, $w_{o3}$; and their p-values as $p_{o1}$, $p_{o2}$, $p_{o3}$. We then defined the InfoXTime effect for attribute *a* as the attribute's mean fitted weight during offers for which that attribute was encoded. Just like the previous analysis, this effect was cross-validated using the same two-step procedure: first to sign-normalize the effect for offer *i* based on the sign of the effect for offer *j*, and then to select whether to include the effect for offer *i* based on the significance of the effect for offer *j*. Specifically, we defined the neuron's

$$InfoXTime_a = (w_{1a} sign(w_{2a}) sig_{2a} + w_{2a} sign(w_{1a}) sig_{1a}) / \max(1, sig_{1a} + sig_{2a})$$

…where the significance indicator $sig_{ia}$ is 1 if the response to offer *i* has a significant effect of attribute *a* ($p < 0.05$) and 0 otherwise. As in the previous analyses, if there is a true effect that is consistently measurable as significant with a consistent sign then $InfoXTime_a$ would be positive, while under the null hypothesis of no true coding $InfoXTime_a$ would have an expected value of 0. Finally, we computed the total InfoXTime effect by summing the InfoXTime effects for the three attributes:

$$InfoXTime = \sum_a InfoXTime_a$$

As in the previous analysis, we then tested whether the median InfoXTime effect across the population of neurons was different from 0 (signed-rank test). The above analysis was restricted to the standard offer response window. We also plotted the full timecourse of the InfoXTime effect using a sliding analysis in which separate fits were done in 4 ms increments on the set of single trial spike density functions (converted to normalized firing rate and smoothed with a Gaussian kernel, σ = 40 ms), then the results were further slightly smoothed (Gaussian kernel, σ = 8 ms).

*Value coding index (Fig 6)*. We defined a value coding index to quantify the extent to which the attribute-related offer responses of each individual neuron aligned with our behaviorally-derived estimate of how animals weighted those attributes to compute the subjective value of those offers. To do this, we first fit each neuron using a version of main model described above, which we will refer to here as the "attribute model" because it allows the neuron to have separate weights for each of the 10 separate attributes described above that influenced the animal's choices ("Attribute Model", Table S3). We then fit each neuron with a new model called the "value model", which only had a single attribute, "Value", equal to the estimated subjective value of the offer derived from the main model (using the procedure described above; "Value Model", Table S3). Thus, the value model represents the hypothesis that the neuron's attribute-related activity is fully explained by encoding of a single variable, the offer's fully integrated subjective value; or equivalently, that the neuron's weighting of the offer's many attributes to govern its response, is perfectly correlated with the animal's weighting of the offer's many attributes to compute subjective value and guide its decisions.

Finally, we quantified what fraction of the above-chance variance in the neuron's activity that was explained by the attribute model could also be explained by the value model. To do this, we used a measure based on R-

squared ($R^2$) to quantify the fraction of the response variance that each model explained. Specifically, to correct for the fact that the attribute model had more parameters than the value model, we computed the shuffle-corrected R-squared ($R^2$corr), defined as the $R^2$ computed on the neuron's real data minus the mean of the $R^2$ computed on 100 shuffled datasets in which the neural responses to the offers were shuffled across trials. Thus, $R^2$corr quantified the fraction of variance of neural responses that the model explained, above and beyond the chance level expected under the null hypothesis that attributes and values had no influence on the neuron's activity. We then calculated mean$R^2$corr for each model as the average of the $R^2$corr that were computed from separate fits of that model to the neuron's Offer 1 and Offer 2 responses. Finally, we defined the value coding index with the equation:

$$ValueCodingIndex = c\left(\frac{meanR^2corr_{value\ model}}{meanR^2corr_{attribute\ model}}\right)$$

…where the function $c(x) = \max(0,\min(1,x))$ clamps the index between 0 and 1 (for rare cases where the index was slightly < 0 or > 1 such as due to small variations in the shuffling used for the shuffle correction; n=8 LHb, n=8 Pal, n=5 STN). Importantly, because the value coding index is expressed as a ratio relative to the corrected variance explained by the attribute model, it is only meaningful and reliably estimated for neurons for which a meaningful fraction of the variance was explained by the attribute model. Therefore, we only computed this index for neurons that had strong attribute effects, which we defined as neurons for which the mean $R^2$corr for attribute model was greater than or equal to 0.1 and that were significantly attribute responsive.

*Comparison of real vs simulated data under different hypotheses (Fig 6).* We used the following procedure to generate simulated datasets representing the hypothesis that all attribute-responsive neurons purely encoded subjective value ("full integration"). First, for each real attribute-responsive neuron's response to each of the two offers, we generated the parameters for a corresponding simulated neuron representing how that neuron would have responded to that offer if it had purely encoded subjective value. There were three parameters for each neuron: it's level of spiking noise, $\sigma_{activity}$; its weight for how strongly it signals value with its activity, $\beta_{value}$; and the vector of weights for how the animal constructed subjective value while the neuron was recorded, $\beta_{attribute}$. Then for each offer on each trial in the real dataset, we generated simulated firing rates representing how the simulated neuron would respond to that offer, $r_{sim,value}$, drawn from the distribution:

$$r_{sim,value} \sim N(\,(x_{attribute} \times \beta_{attribute})\beta_{value}, \sigma_{activity})$$

…where N(μ, σ) is a normal distribution with mean μ and standard deviation σ, and $x_{attribute}$ is a vector of the attributes of the offer (e.g. < E[r], Unc[r], Info, … >). We set the parameters as follows. To estimate $\sigma_{activity}$, we used the standard deviation of the residuals from the Attribute model's fit to the real neuron's offer response (i.e. representing the trial-to-trial variation in activity that was not explained by attribute-related coding). To estimate $\beta_{value}$, we used the fitted weight of the value term in the Value model's fit to the real neuron's offer response (i.e. the change in the neuron's activity in Hz per log odds of choosing the offer). To model $\beta_{attribute}$, we started with the fitted weights of the attributes in the behavioral model fit to the monkey's full behavioral dataset $\beta_{attribute,full}$. Note that using $\beta_{attribute,full}$ directly for all neurons would produce an unrealistic overestimate of the Value Coding Index, because it would represent the hypothesis that the monkey's 'true' value function was perfectly identical across all trials in all sessions. In practice, we found that the monkeys' value functions had modest variation over time and sessions. For example, if we fit the behavioral model separately for each session, there were modest variations in the fitted attribute weights beyond what would be expected by chance due to sampling error. To account for this, we estimated the degree to which each attribute's 'true' weight varied across sessions, defined as the difference between the variance

across sessions of its fitted weight, and the variance of the fitted weight that would be expected by chance due to sampling error. Specifically, we used the equation Var[$\beta_{true}$] = Var[$\beta_{sess}$] – Var[$\beta_{sess,chance}$], where $\beta_{sess}$ was a vector with the fitted beta weight for that attribute from each session of data from that monkey, and Var[$\beta_{sess,chance}$] was approximated as the square of the mean of the standard errors of the $\beta_{sess}$. We then set $\beta_{attribute}$ for each simulated neuron by starting with $\beta_{attribute,full}$ and then adding normally distributed variation to each attribute's weight based on the estimated 'true' variation of that weight across sessions, i.e. $\beta_{attribute}$ = N( $\beta_{attribute,full}$ , sqrt(Var[$\beta_{true}$])). We obtained similar results by simply adding a fixed level of variation to all attribute weights across neurons, as long as it was in the vicinity of what was observed in the real data.

We used an analogous procedure to simulate datasets representing other hypotheses about neurons coding different attribute-related signals. We used the model:

$$r_{sim,i} \sim N(\,(x_{attribute} \times \beta_{attribute,i})\beta_{signal}, \sigma_{activity})$$

Where each simulation $i$ used a unique $\beta_{attribute,i}$ for each neuron to represent its hypothesis about what attributes it encoded, and used a common $\beta_{signal}$ to represent how strongly it encoded them (analogous to for the $\beta_{value}$ value coding model above). Simulation 1 ("single attributes") represented the hypothesis that each neuron encoded a single attribute. To do this, we pseudorandomly assigned each neuron to encode one single attribute in the model, by setting its $\beta_{attribute,1}$ to max(|$\beta_{attribute}$|) for the encoded attribute and 0 for all other attributes. Simulation 2 ("random mixtures") represented the hypothesis that each neuron encoded a random mixture of attributes with no systematic relationship to value. To do this, we set each neuron's $\beta_{attribute,1}$ equal to $\beta_{attribute}$, then randomly shuffled the vector of weights and randomly flipped their signs (i.e. multiplied each attribute's sign by -1 with 0.5 probability). Simulation 3 ("partial integration") represented the hypothesis that each neuron encoded half of the attributes randomly but integrated the other half of the attributes in a manner consistent with subjective value. To do this, we repeated the procedure for simulation 2, but kept the weights from $\beta_{attribute}$ intact for a randomly selected half of the attributes for each neuron; only the other half of the attributes had their weights shuffled and sign-flipped. Finally, for each neuron we defined $\beta_{signal}$ to capture each neuron's coding strength in an analogous way to $\beta_{attribute}$, but to also accommodate neurons that encode attributes in manners other than subjective value. Specifically, we defined $\beta_{signal}$ = $\sigma_{attributeresponse}$ / $\sigma_{subjectivevalue}$, where $\sigma_{attributeresponse}$ is the standard deviation of the attribute-related component of its responses across trials (according to the Attribute Model's fit to neural activity), and $\sigma_{subjectivevalue}$ is the standard deviation of the subjective values of the offers across the same trials (according to the standard model fit to the animal's behavior). Thus, $\beta_{signal}$ was approximately equal to $\beta_{value}$ for neurons that encoded attributes similar to subjective value (e.g. many LHb neurons), because $\beta_{value}$ described how neurons approximately converted variations in subjective value (in units of log odds) to variations in neural responses (Hz). For neurons that encoded attributes in other manners, $\beta_{signal}$ still represented an estimate of how strongly they encoded those attributes, normalized as if they varied across trials in a manner similar to subjective value.

*Analysis of choice-predictive activity (Fig 7-8)*. We computed a choice predictive index as a measure of how variations in LHb neuronal activity above and beyond those accounted for by our neuronal model, were predictive of variations in choice behavior above and beyond those accounted for by our behavioral model. To do this, we first fit the behavioral choice data with the main model. To ensure that the behavioral fit reflected the animal's behavioral policy as closely as possible during recording of the neuron, we used a fit that was restricted to the subset of the behavioral data that was collected during the recording of that neuron (we obtained similar results when using the behavioral fits to the full behavioral dataset). Next, we fit the neuronal offer responses with a version of the main model. We did separate fits for the neuronal responses to Offer 1

and to Offer 2. To allow comparison across neurons, rather than fitting raw firing rates, we fit “normalized value signals”, defined as the normalized firing rate that has been sign-flipped based on the that neuron’s fitted sign of value coding (from the value coding index analysis described above). Thus, on average, more positive normalized value signals correspond to activity reflecting higher offer values. In addition, to control for the possibility that variations in the neuronal response to the currently fitted offer might be influenced by the properties of the other offer, this was done based on a fit of a versions of the model that, in addition to the terms representing how the response to the currently fitted offer response depended on each attribute of that offer, also included an analogous set of terms for how the response to the currently fitted offer depended on each attribute of the other offer. Thus, for each trial, these fits provided us with a predicted choice probability and predicted normalized firing rates for Offer 1 and Offer 2. Using these, for each offer *i*, we computed the behavioral residual $\Delta behavioral_i$ and the neural residual $\Delta neural_i$ , using the following equations:

$$\Delta behavioral_i = Chose(i) - E_{behavmodel}[Chose(i)]$$
$$\Delta neural_i = NVS(i) - E_{neuralmodel}[NVS(i)]$$

Where Chose(*i*) = 1 if offer *i* was chosen and 0 otherwise, NVS(i) is the normalized value signal in response to offer *i*, and $E_{model}[x]$ is the expected value of x under the specified model. Finally, to improve its interpretability as measure relating behavioral variations to neural variations, we excluded a small subset of trials that had very low dynamic range of behavioral residuals to relate to activity, due to large offer value differences inducing very deterministic choices. Specifically, we excluded trials in which the behavioral model predicted that the animal would choose a specific offer with > 95% probability and that prediction was correct (i.e. trials in which $|\Delta behavioral_i|$ was bounded to be <= 0.05). This did not affect the pattern of results or conclusions.

We then defined the choice predictive index for each Offer *i* as the Spearman’s rank correlation between the behavioral and neural residuals:

$$ChoicePredictiveIndex_i = corr(\Delta behavioral_i, \Delta neural_i)$$

…where the behavioral residual $\Delta behavioral_i$ = (Chose Offer *i*?) – (predicted p(choose Offer *i*)), and the neural residual $\Delta neural_i$ = (normalized value signal for Offer *i*) – (predicted normalized value signal for Offer *i*). In addition, to pool neural responses over the two offers to establish a total prediction about each trial’s choice, we computed a total choice predictive index for each neuron as the correlation between the residual choice of Offer 2 and the difference in neural residuals reflecting the net difference in normalized residual value signal favoring Offer 2:

$$ChoicePredictiveIndex = corr(\Delta behavioral_2, \Delta neural_2 - \Delta neural_1)$$

To examine the relation between behavioral and neural residuals at the population level, we calculated a population average relationship between residual value signals and residual choice, separately for Offer 1 and Offer 2. Specifically, for each neuron, we normalized its residual value signals by dividing the residual value signal on each trial by the SD of its residual value signals over all trials, and then clamping them between -4 and +4. Then we plotted the relationship between, and calculated the correlation between, the normalized residual value signals and residual choices, pooling over all neurons and all trials. To test for a difference between Offer 1 and Offer 2, we performed the above procedure on bootstrapped datasets (n=200 bootstraps over neurons) and tested whether the bootstrap confidence interval of the difference between the correlations for Offer 1 and Offer 2 excluded 0.

As a further test of whether LHb residual value signals could be used to predict choice behavior, we asked whether they could be used to improve the performance of our behavioral models. First, for each neuron, we used the fit of the main behavioral model described above to derive an estimate of the subjective values of Offer 1 and Offer 2 on each trial ("V1" and "V2", respectively), as described above. Then, we fit a new behavioral model (Table S3) that had two attributes: (1) "V2-V1", equal to the difference in subjective value between the two offers, and (2) "LHbResid2-LHbResid1", equal to the difference in normalized residual value signals ($\Delta neural_2 - \Delta neural_1$). To further separate the influences of Offer 1 vs Offer 2, we also fit alternate models in which LHbResid1 and LHbResid2 were included as separate attributes, to allow them to be fitted with separate weights for predicting behavioral choices (Table S3).

*Full formulation of models.*

In this section we provide a full description for each model, following the general format described in Methods. Briefly, each model was fit as a GLM, which can be described by its regressors, distribution, and link function. The behavioral version of each model was fit to behavioral binary choice data using a binomial distribution and a logistic link function. The neural version of each model was fit to neural firing rate data using a normal distribution and an identity link function.

We will first lay out the types of models we used, and then then specify each individual model by specifying which type of model it is and listing its regressors. Each model could come in up to three versions, depending on how it was used to fit behavioral or neural data.

Version 1, the *behavioral version*. This attempted to fit behavioral choices of offer 2 vs offer 1 as a function of the difference between the attributes of the two offers:

$$\log\left(\frac{p(\text{choose offer 2})}{p(\text{choose offer 1})}\right) = \beta_1(x_{2,1} - x_{1,1}) + \beta_2(x_{2,2} - x_{1,2}) + \cdots + \beta_n(x_{2,n} - x_{1,n})$$

Version 2, the *neural single-offer version*. This attempted to fit neuronal firing rates in response to a specific offer (e.g. offer 1) as a function of the attributes of that offer, and the normally distributed error term ε:

$$Rate(\text{offer } i) = \beta_{i,0} + \beta_{i,1}x_{i,1} + \beta_{i,2}x_{i,2} + \cdots + \beta_{i,n}x_{i,n} + \varepsilon$$

Version 3, the *neural both-offers version*. This attempted to fit neuronal firing rates in response to a specific offer (e.g. offer 1) as a function of the attributes of *both* offers, allowing each offer's attributes to have different weights (i.e. weights $\beta_i$ for the attributes $x_i$ of offer *i* and weights $\beta_j$ for the attributes $x_j$ of the other offer *j*):

$$Rate(\text{offer } i) = \beta_{i,0} + \beta_{i,1}x_{i,1} + \beta_{i,2}x_{i,2} + \cdots + \beta_{i,n}x_{i,n} + \beta_{j,1}x_{j,1} + \beta_{j,2}x_{j,2} + \cdots + \beta_{j,n}x_{j,n} + \varepsilon$$

For all models, the regressors were divided into two sets, $x_{attrib}$ representing motivational attributes of the offer and $x_{bias}$ representing a small number of neural/behavioral bias terms (e.g. a constant factor, the spatial location of the offer on the screen, etc.). The models differed in $x_{attrib}$, while $x_{bias}$ was consistent across models. Specifically, the bias terms for offer *i* in behavioral models were:

$$x_{bias,i} = < x_{left,i}, x_{right,i}, x_{second,i} >$$

And in neural models were:

$$x_{bias,i} = < x_{left,i}, x_{right,i} >$$

Where:

$x_{left,i}$ = 1 if offer *i* was on the left side of the screen and 0 otherwise
$x_{right,i}$ = 1 if offer *i* was on the right side of the screen and 0 otherwise
$x_{second,i}$ = 1 if offer *i* was offer 2 and 0 otherwise.

*List of model motivational attribute regressors and versions*

Model 1 ("Distrib SD"), behavioral version.
$x_{attrib}$ = < E[r], Unc[r], Info, Info x E[r], Info x Unc[r] >
where Unc[r] = SD[r]

Model 2 ("Distrib Range"), behavioral version. Same as model 1 but Unc[r] = Range[r].

Model 3 ("Distrib Entropy"), behavioral version. Same as model 1 but Unc[r] = Entropy[r].

Model 4 ("Distrib None"), behavioral version.
$x_{attrib}$ = < E[r], Info, Info x E[r] >
where Unc[r] = Entropy[r]

Model 5 ("Distrib UncType"), behavioral version.
$x_{attrib}$ = < E[r], $Unc_{25/50/25}$, $Unc_{50/50}$, Info, Info x E[r], Info x $Unc_{25/50/25}$, Info x $Unc_{50/50}$ >
$Unc_{25/50/25}$ = 1 if the offer was a 25/50/25 offer, and 0 otherwise
$Unc_{50/50}$ = 1 if the offer was a 50/50 offer, and 0 otherwise

Model 6 ("Distrib UncTypeDetailed"), behavioral version.
$x_{attrib}$ = < E[r], $Unc_{25/75,lo}$, $Unc_{25/75,hi}$, $Unc_{25/50/25,lo}$, $Unc_{25/50/25,hi}$, $Unc_{50/50,lo}$, $Unc_{50/50,hi}$, Info, Info x E[r], Info x $Unc_{25/75,lo}$, Info x $Unc_{25/75,hi}$, Info x $Unc_{25/50/25,lo}$, Info x $Unc_{25/50/25,hi}$, Info x $Unc_{50/50,lo}$, Info x $Unc_{50/50,hi}$ >
$Unc_{25/75,lo}$ = 1 if the offer was a 25/75 offer with a relatively low SD[r], and 0 otherwise
$Unc_{25/75,hi}$ = 1 if the offer was a 25/75 offer with a relatively high SD[r], and 0 otherwise
$Unc_{25/50/25,lo}$ = 1 if the offer was a 25/50/25 offer with a relatively low SD[r], and 0 otherwise
$Unc_{25/50/25,hi}$ = 1 if the offer was a 25/50/25 offer with a relatively high SD[r], and 0 otherwise
$Unc_{50/50,lo}$ = 1 if the offer was a 50/50 offer with a relatively low SD[r], and 0 otherwise
$Unc_{50/50,hi}$ = 1 if the offer was a 50/50 offer with a relatively high SD[r], and 0 otherwise

Model 7 ("Human Info Timing"), behavioral version.
$x_{attrib}$ = < E[r], EarlyInfo >

Model 8 ("Clock SD"), behavioral and neural versions.
$x_{attrib}$ = < E[r], Tout, Unc[r], E[r] x Tout, E[r] x Unc[r], Info, Info x Tout, Info x Tadv, Info x Unc[r], Info x Tadv x Unc[r] >
Tadv = Tout - Tcue

Unc[r] = SD[r]

Model 9 (“Clock Range”), behavioral and neural versions. Same as model 7 but Unc[r] = Range[r].

Model 10 (“Clock Entropy”), behavioral and neural versions. Same as model 7 but Unc[r] = Entropy[r].

Model 11 (“Clock None”), behavioral and neural versions.

$x_{attrib}$ = < E[r], Tout, E[r] x Tout, Info, Info x Tout, Info x Tadv >

Model 12 (“Clock UncType”), behavioral and neural versions.

$x_{attrib}$ = < E[r], Tout, $Unc_{25/50/25}$, $Unc_{50/50}$, E[r] x Tout, E[r] x $Unc_{25/50/25}$, E[r] x $Unc_{50/50}$, Info, Info x Tout, Info x Tadv, Info x $Unc_{25/50/25}$, Info x $Unc_{50/50}$, Info x Tadv x $Unc_{25/50/25}$, Info x Tadv x $Unc_{50/50}$>

Tadv = Tout - Tcue

$Unc_{25/50/25}$ = 1 if the offer was a 25/50/25 offer, and 0 otherwise

$Unc_{50/50}$ = 1 if the offer was a 50/50 offer, and 0 otherwise

Model 13 (“Clock SD with Info x E[r]”), behavioral and neural versions.

$x_{attrib}$ = < E[r], Tout, Unc[r], E[r] x Tout, E[r] x Unc[r], Info, Info x E[r], Info x Tout, Info x Tadv, Info x Unc[r], Info x Tadv x Unc[r] >

Unc[r] = SD[r]

Model 14 (“Clock Info x Time only”), behavioral and neural versions.

$x_{attrib}$ = < E[r], Tout, Unc[r], E[r] x Tout, E[r] x Unc[r], Info, Info x Tout, Info x Tadv, >

Unc[r] = SD[r]

Model 15 (“Clock Info x Unc only”), behavioral and neural versions.

$x_{attrib}$ = < E[r], Tout, Unc[r], E[r] x Tout, E[r] x Unc[r], Info, Info x Unc >

Unc[r] = SD[r]

Model 16 (“Clock Info main effect only”), behavioral and neural versions.

$x_{attrib}$ = < E[r], Tout, Unc[r], E[r] x Tout, E[r] x Unc[r], Info >

Unc[r] = SD[r]

Model 17 (“Clock Simple Timing”), behavioral version.

$x_{attrib}$ = < E[r], Tout, Unc[r], E[r] x Unc[r], Info, Info x Unc[r], Info x $T_{EE}$, Info x $T_{EL}$, Info x $T_{LL}$ >

Unc[r] = SD[r]

$T_{EE}$ = 1 if Tcue is in the earliest 1/3 of Tcues and Tout is in the earliest 1/3 of Touts

$T_{EL}$ = 1 if Tcue is in the earliest 1/3 of Tcues and Tout is in the latest 1/3 of Touts

$T_{LL}$ = 1 if Tcue is in the latest 1/3 of Tcues and Tout is in the latest 1/3 of Touts

Model 18 (“Attribute model”), neural versions. This is the same as the Model 8 (“Clock SD”), but specifically using version 3 of the model (neural both-offers version) to permit the model as much freedom as possible to fit any relationship between the neural response to each offer and the attributes of both of the offers.

$x_{attrib}$ = < E[r], Tout, Unc[r], E[r] x Tout, E[r] x Unc[r], Info, Info x Tout, Info x Tadv, Info x Unc[r], Info x Tadv x Unc[r] >

Tadv = Tout - Tcue

Unc[r] = SD[r]

Model 19 ("Value model"), neural versions. In the following models, the "Value" for each offer during the neuronal recording (in units of log odds of choice) was estimated based on the fitted model weights for Model 8 ("Clock SD") fit to each animal's behavioral dataset. Value1 and Value2 correspond to the estimated values of offers 1 and 2, respectively.

$x_{attrib}$ = < $Value_i$, $Value_j$>

In the following models, the residual neural activity in response to each offer ($\Delta neural_1$ and $\Delta neural_2$) was computed using the residuals from Model 18, as described in Methods.

Model 20 ("ChoicePred"), behavioral version.

$x_{attrib}$ = < $Value_2$ – $Value_1$, $\Delta neural_2$ - $\Delta neural_1$ >

Model 21 ("ChoicePred by Offer"), behavioral version.

$x_{attrib}$ = < $Value_2$ – $Value_1$, $\Delta neural_1$ , $\Delta neural_2$ >

In the following models used for stimulation sessions, $Value_1$ and $Value_2$ were estimated based on the fitted model weights for Model 8 ("Clock SD") during stimulation sessions using only data from trials where no stimulation was performed. $Stim_1$ and $Stim_2$ were indicator variables indicating whether stimulation was performed during offer 1 or offer 2, respectively.

Model 22 ("StimPred by Offer"), behavioral version.

$x_{attrib}$ = < $Value_2$ – $Value_1$, $Stim_1$, $Stim_2$ >

Model 23 ("StimPred None"), behavioral version.

$x_{attrib}$ = < $Value_2$ – $Value_1$>

Model 24 ("StimPred Stim2"), behavioral version.

$x_{attrib}$ = < $Value_2$ – $Value_1$, $Stim_2$ >

Model 25 ("StimPred Stim2xV2-1"), behavioral version.

$x_{attrib}$ = < $Value_2$ – $Value_1$, $Stim_2$, $Stim_2$ x ($Value_2$ – $Value_1$) >

Model 26 ("StimPred Stim2xV2"), behavioral version.

$x_{attrib}$ = < $Value_2$ – $Value_1$, $Stim_2$, $Stim_2$ x $Value_2$ >

Finally, Model 27 ("StimPred Stim2xAttrib") was fit for each individual by starting with Model 24 ("StimPred Stim2") and then augmenting it with additional parameters with an iterative procedure. We first tested whether the fit was significantly improved by adding a $Stim_2$ x Attribute interaction using any of the 10 attributes from Model 8 ("Clock SD"), specifically: E[r], Tout, Unc[r], E[r] x Tout, E[r] x Unc[r], Info, Info x Tout, Info x Tadv, Info x Unc[r], Info x Tadv x Unc[r]. If it was not, we terminated the procedure. If it was, we added the significant attribute with the lowest p-value and then repeated this procedure using the remaining attributes.

## Supplemental-only References


104 Fiorillo, C. D., Tobler, P. N. & Schultz, W. Discrete coding of reward probability and uncertainty by dopamine neurons. *Science* **299**, 1898-1902 (2003).

105 Monosov, I. E. & Hikosaka, O. Selective and graded coding of reward uncertainty by neurons in the primate anterodorsal septal region. *Nat Neurosci* **16**, 756-762 (2013).

106 Schultz, W. *et al.* Explicit neural signals reflecting reward uncertainty. *Philos Trans R Soc Lond B Biol Sci* **363**, 3801-3811 (2008).

107 O'Neill, M. & Schultz, W. Coding of reward risk by orbitofrontal neurons is mostly distinct from coding of reward value. *Neuron* **68**, 789-800 (2010).

108 Tobler, P. N., Fiorillo, C. D. & Schultz, W. Adaptive coding of reward value by dopamine neurons. *Science* **307**, 1642-1645 (2005).

109 Robinson, D. A. Eye movements evoked by collicular stimulation in the alert monkey. *Vision Res* **12**, 1795-1808 (1972).

110 Bruce, C. J., Goldberg, M. E., Bushnell, M. C. & Stanton, G. B. Primate frontal eye fields. II. Physiological and anatomical correlates of electrically evoked eye movements. *J Neurophysiol* **54**, 714-734 (1985).

111 Best, D. J., ; Roberts, D.E. Algorithm AS 89: The Upper Tail Probabilities of Spearman's rho. *Applied Statistics* **24**, 377-379 (1975).
