# Supplementary material for: A neural mechanism for conserved value computations integrating information and rewards
Source: Nat Neurosci. 2024 Jan 4;27(1):159–75. doi: 10.1038/s41593-023-01511-4 (PMC10774124; doi:10.1038/s41593-023-01511-4)
Supplement: Supplementary file 2 — Reporting Summary [file 41593_2023_1511_MOESM2_ESM.pdf]

nature portfolio

Corresponding author(s): Ilya Monosov

Last updated by author(s): Oct 18, 2023

Reporting Summary

Nature Portfolio wishes to improve the reproducibility of the work that we publish. This form provides structure for consistency and transparency in reporting. For further information on Nature Portfolio policies, see our Editorial Policies and the Editorial Policy Checklist.

Statistics

For all statistical analyses, confirm that the following items are present in the figure legend, table legend, main text, or Methods section.

n/a	Confirmed	
☐	☒	The exact sample size (*n*) for each experimental group/condition, given as a discrete number and unit of measurement
☐	☒	A statement on whether measurements were taken from distinct samples or whether the same sample was measured repeatedly
☐	☒	The statistical test(s) used AND whether they are one- or two-sided<br>*Only common tests should be described solely by name; describe more complex techniques in the Methods section.*
☐	☒	A description of all covariates tested
☐	☒	A description of any assumptions or corrections, such as tests of normality and adjustment for multiple comparisons
☐	☒	A full description of the statistical parameters including central tendency (e.g. means) or other basic estimates (e.g. regression coefficient) AND variation (e.g. standard deviation) or associated estimates of uncertainty (e.g. confidence intervals)
☐	☒	For null hypothesis testing, the test statistic (e.g. *F*, *t*, *r*) with confidence intervals, effect sizes, degrees of freedom and *P* value noted<br>*Give P values as exact values whenever suitable.*
☒	☐	For Bayesian analysis, information on the choice of priors and Markov chain Monte Carlo settings
☒	☐	For hierarchical and complex designs, identification of the appropriate level for tests and full reporting of outcomes
☐	☒	Estimates of effect sizes (e.g. Cohen's *d*, Pearson's *r*), indicating how they were calculated

Our web collection on statistics for biologists contains articles on many of the points above.

Software and code

Policy information about availability of computer code

Data collection	MATLAB r2015b and r2022a, psiturk v3.1.0, jspsych v6.0.4, PyElectrode v.0.3.0
Data analysis	MATLAB r2022a

For manuscripts utilizing custom algorithms or software that are central to the research but not yet described in published literature, software must be made available to editors and reviewers. We strongly encourage code deposition in a community repository (e.g. GitHub). See the Nature Portfolio guidelines for submitting code & software for further information.

Data

Policy information about availability of data

All manuscripts must include a data availability statement. This statement should provide the following information, where applicable:

- Accession codes, unique identifiers, or web links for publicly available datasets
- A description of any restrictions on data availability
- For clinical datasets or third party data, please ensure that the statement adheres to our policy

Data from this study is available upon request.

Research involving human participants, their data, or biological material

Policy information about studies with human participants or human data. See also policy information about sex, gender (identity/presentation), and sexual orientation and race, ethnicity and racism.

Reporting on sex and gender	Participants self-reported gender (task 1, 294 female, 282 male, 20 other, 2 no response; task 2, 116 females, 124 males, 2 other, 2 no response) which is noted in the dataset with consent. The results were not specific to one gender and preliminary analysis indicated that the major results were similar between genders so the data was pooled for further analysis.
Reporting on race, ethnicity, or other socially relevant groupings	Participants were not asked to disclose race, ethnicity, or other social groupings.
Population characteristics	Participants self-reported age (mean 26.06 years, SD 11.15 years). The inclusion criteria as described below included no history of neurological or psychiatric illness (self-reported). Our study did not aim to determine relationships between behavior and genotypes and/or medical diagnoses and hence those population characteristics were not collected.
Recruitment	Participants were recruited on Amazon MTurk. The ad for our Human Intelligence Task (HIT) was posted in the standard mTurk listings. Those interested in performing the HIT accepted the assignment and completed the task. Participants were recruited with the restrictions of being healthy adults aged 18 to 55 years old, with no history of neurological or psychiatric illness, located in the USA and reading English (the language used to administer the study), and normal or corrected to normal visual acuity (to ensure they could participate in the task). They performed the task for monetary compensation. Hence the results may be most applicable to those demographic groups and to individuals who are able and motivated to engage in online tasks for monetary compensation
Ethics oversight	Washington University IRB

Note that full information on the approval of the study protocol must also be provided in the manuscript.

Field-specific reporting

Please select the one below that is the best fit for your research. If you are not sure, read the appropriate sections before making your selection.

☒ Life sciences ☐ Behavioural & social sciences ☐ Ecological, evolutionary & environmental sciences

For a reference copy of the document with all sections, see nature.com/documents/nr-reporting-summary-flat.pdf

Life sciences study design

All studies must disclose on these points even when the disclosure is negative.

Sample size	We collected data from n=2 to n=4 macaque monkeys for each component of the animal part of the study (task 1 behavior, n=4; task 2 behavior, n=3; task 2 neuronal activity and stimulation, n=2) with large numbers of trials and neurons for each animal (n>1000 trials for each animal and task, n>100 neurons for each animal in each major brain area studied). No statistical methods were used to pre-determine sample sizes. These sample sizes were chosen with the rationale that they are greater than or equal to the standard number of animals (n=2), trials, and neurons used in the majority of non-human primate neurophysiology research studies to produce reproducible results.
Data exclusions	Several analyses focused on task-related activity of neurons and hence excluded neurons with no significant effect of any task-related variable (as is standard in neurophysiology research where a fraction of neurons is commonly non-responsive to the task)
Replication	Animal behavior main results were successfully replicated in n=4 animals (task 1) and n=3 animals (task 2). Animal neural recording and stimulation main results were successfully replicated in n=2 animals. Human main results were each tested in one sample of n=565 (task 1) and n=210 (task 2)
Randomization	Experiments were performed in within-subject designs (each subject tested in each condition, on different random subsets of trials)
Blinding	There was no group allocation to be blinded to as experiments were performed in within-subjects designs. Data collection and analysis were not performed blind to the conditions of the experiments.

Reporting for specific materials, systems and methods

We require information from authors about some types of materials, experimental systems and methods used in many studies. Here, indicate whether each material, system or method listed is relevant to your study. If you are not sure if a list item applies to your research, read the appropriate section before selecting a response.

Materials & experimental systems

n/a	Involved in the study
☒	☐ Antibodies
☒	☐ Eukaryotic cell lines
☒	☐ Palaeontology and archaeology
☐	☒ Animals and other organisms
☒	☐ Clinical data
☒	☐ Dual use research of concern
☒	☐ Plants

Methods

n/a	Involved in the study
☒	☐ ChIP-seq
☒	☐ Flow cytometry
☒	☐ MRI-based neuroimaging

Animals and other research organisms

Policy information about studies involving animals; ARRIVE guidelines recommended for reporting animal research, and Sex and Gender in Research

Laboratory animals	Four adult male macaque monkeys (Macaca mulatta, age 7-9 years)
Wild animals	None
Reporting on sex	n=4 male animals; sample size was not sufficient for sex-based analysis
Field-collected samples	None
Ethics oversight	Washington University Institutional Animal Care and Use Committee

Note that full information on the approval of the study protocol must also be provided in the manuscript.
